# The Influence of Peptidases in Intestinal Brush Border Membranes on the Absorption of Oligopeptides from Whey Protein Hydrolysate: An Ex Vivo Study Using an Ussing Chamber

**DOI:** 10.3390/foods9101415

**Published:** 2020-10-07

**Authors:** Luísa Ozorio, Caroline Mellinger-Silva, Lourdes M. C. Cabral, Julien Jardin, Gaelle Boudry, Didier Dupont

**Affiliations:** 1 Instituto de Química, Universidade Federal do Rio de Janeiro, Rio de Janeiro 21044020, Brazil; luisa.ozorio@gmail.com; 2 EMBRAPA Agroindústria de Alimentos, Rio de Janeiro 23020470, Brazil; caroline.mellinger@embrapa.br (C.M.-S.); lourdes.cabral@embrapa.br (L.M.C.C.); 3 Science and Technology of Milk and Eggs (STLO), INRAE, Institut Agro, 35042 Rennes, France; julien.jardin@inrae.fr; 4 Institut Numecan, INRAE, INSERM, University Rennes, 35590 Saint-Gilles, France; gaelle.boudry@inrae.fr

**Keywords:** brush border peptidases, intestinal permeation, oligopeptides absorption, Ussing chamber, whey protein hydrolysate

## Abstract

For many years, it was believed that only amino acids, dipeptides, and tripeptides could be absorbed and thus reach the bloodstream. Nowadays, the bioavailability of oligopeptides is also considered possible, leading to new research. This pilot study investigates the activity of brush border enzymes on undigested whey protein hydrolysate (WPH) and on simulated intestinal digested (ID) whey hydrolysate and the subsequent absorption of resultant peptides through the proximal jejunum of a 7-week old piglet setup in an Ussing chamber model. Amongst all samples taken, 884 oligopeptides were identified. The brush border peptidase activity was intense in the first 10 min of the experiment, producing several new peptides in the apical compartment. With respect to the ID substrate, 286 peptides were detected in the basolateral compartment after 120 min of enzyme activity, originating from β-lactoglobulin (60%) and β-casein (20%). Nevertheless, only 0.6 to 3.35% of any specific peptide could pass through the epithelial barrier and thus reach the basolateral compartment. This study demonstrates transepithelial jejunum absorption of whey oligopeptides in an ex vivo model. It also confirmed the proteolytic activity of brush border enzymes on these oligopeptides, giving birth to a myriad of new bioactive peptides available for absorption.

## 1. Introduction

Whey proteins have a high nutritional value containing bioactive peptides within their structures which may be released either via food processing or during digestion. In the gastrointestinal tract, numerous enzymes can release these peptides from source proteins: the process begins in the stomach with the activity of pepsin [1,2]. However, pepsin is only able to hydrolyze 10–15% of dietary proteins. It is the pancreatic proteases, trypsin, chymotrypsin, elastase, and carboxypeptidases that continue the digestion process in the intestine, breaking up large molecules into free amino acids (approximately 30%) and oligopeptides (approximately 70%) [3]. 

In the small intestine, peptidases in the brush border membrane (BBM) (including aminopeptidases, carboxypeptidases, endopeptidases, and dipeptidases) are responsible for the final stage of peptide digestion (prior to their absorption into the enterocytes) by reducing most poly- and oligopeptides to their monomer constituents [4]. Some dairy peptides are known to affect the intestinal lumen by stimulating mucus production, modulating mineral absorption, increasing satiety, limiting inflammation, and changing the incretin response [5]. 

Before being released in the bloodstream, peptides may undergo further intracellular digestion by cytosolic, lysosomal, and microsomal enzymes: those not absorbed are usually excreted in the urine or bile [4,6]. Those peptides reaching the bloodstream are yet susceptible to action from plasma enzymes with only the most resistant reaching target organs to play out roles as antioxidant, antihypertensive, opioid, anticancer, or antidiabetic molecules [7,8,9,10].

With respect to in vitro food digestion models, the international network INFOGEST has set out a protocol that was recently updated [11] and which has since been used in numerous studies. However, such a protocol for the in vitro simulation of intestinal absorption is not yet available. 

Caco-2 cells derive from a colon carcinoma but are able to differentiate into similar to mature human enterocytes and form monolayers. Several studies have used this physical model to simulate the absorption of different food materials, including milk-derived peptides [12,13,14]. Nevertheless, there remain concerns over their capability to mimic human intestinal permeation. Whereas the Caco-2 cell monolayer model is simply constructed from enterocytes, the human intestinal epithelium comprises the interaction of enterocytes, goblet cells, endocrine cells, and M cells [15]. An overexpression of the tight junction proteins makes the monolayer resemble more the colon tissue than the small intestine, resulting in a poor permeability for hydrophilic molecules and a low carrier-mediated absorption of compounds. Furthermore, there is no mucus layer on the luminal side of Caco-2 cells, which also limits the relevance of the model, since mucus secreting goblet cells represent the second most frequent cell type in the human intestinal epithelium [15,16,17]. 

In this sense, other physical absorption models have been considered, including the Ussing chamber (UC), which uses animal tissue to simulate the permeation of food substrates through the intestinal wall. This technique has been extensively used to study drug absorption, and several reviews have shown a satisfactory correlation with the absorption rates measured on human volunteers, particularly with respect to passively transported compounds [18,19,20]. UC thus may allow investigation into the absorption of dietary peptides that cannot easily be done in vivo. Indeed, the presence of plasma proteases that further hydrolyze peptides reaching the bloodstream and of blood proteins make the untargeted identification of peptides difficult. Tissue from various animals may be used depending on the availability and purpose of the research. For human nutrition, pigs are considered the best non-primate model, since they have a large single-stomach and a comparable gut physiology to humans [21]. However, a literature search revealed only one study [22] using the UC model to investigate the permeation of amino acids from whey proteins, and no such research on the dietary absorption of whey oligopeptides was evident. 

Different pathways may be involved in the absorption of amino acids and peptides. The uptake of amino acids and di- and tripeptides generally occur via either passive diffusion, tight junctions, endocytosis, or transcellular carrier-mediated transport. There are also some transmembrane co-transporters such as the proton-dependent H+/peptide PepT1 and the sodium-dependent oligopeptide transporters SOPT1 and SOPT2. For many years, it was believed that only free amino acids (along with di- and tripeptides) could be transported through the brush border membrane (BBM) [2], and there is little evidence of the absorption of oligopeptides and of the possible mechanisms implied for such a process. Clarification on this matter remains the subject of further investigation. 

We have previously produced a whey protein hydrolysate that exhibits interesting vasodilatation properties [23]. This work demonstrated that the vasodilatory potency of this hydrolysate was even enhanced in the gastrointestinal tract during digestion through the release of bioactive peptides by digestive proteases. Likewise, this study investigates the activity of peptidases in the intestinal BBM on undigested (WPH) and digested (ID) whey hydrolysates and the subsequent absorption of these peptides by piglet proximal jejunum. The study was carried out using a UC apparatus to model ex vivo permeation across a transepithelial intestinal membrane. The approach represents another step towards understanding oligopeptides absorption, their bioavailability, and possible expression of their biological activity. 

## 2. Materials and Methods 

### 2.1. Substrate and Reagents

Bovine whey protein concentrate containing 88% of protein (WPC88) and commercial pepsin from porcine gastric mucosa were acquired from Alibra Ingredientes Ltda (Campinas, SP, Brazil) and Bela Vista Produtos Enzimáticos Ind. e Com. Ltda. (Bela Vista, SC, Brazil), respectively. Other reagents were purchased from Sigma-Aldrich (St. Louis, MO, USA). 

### 2.2. Whey Enzymatic Hydrolysis 

One hundred-liter batches of WPC88 were hydrolyzed in a pilot plant as described previously [23]. In summary, a 1.25% (*w*/*v*) solution of WPC88 was prepared and the pH was adjusted to 2 (using 1 M HCl). Pepsin was added (5.34 µU·g^−1^ of protein), and the hydrolysis was maintained for 3 h at 37 °C with constant stirring. The pH and temperature were held constant during the whole experiment. Pepsin was inactivated at the end by raising the temperature to 80 °C for 5 min. The hydrolyzed whey solution was then spray dried (Niro atomizer, Copenhagen, Denmark. Inlet temperature: 170 °C; outlet temperature: 90 °C), and the resulting powder maintained at −20 °C until required.

### 2.3. In Vitro Digestion of Whey Hydrolysate

In vitro gastrointestinal digestion of WPH powder followed the harmonized protocol for in vitro digestion set out by INFOGEST [24]. The compositions of simulated gastric fluid (SGF) and simulated intestinal fluid (SIF) are given in Table 1 based on Brodkorb et al. [11]. Noting that WPH was rehydrated as a solution, the oral phase of the in vitro digestion was not performed. WPH powder was dispersed (8% *w*/*v*) in distilled water and mixed with simulated gastric fluid containing pepsin from porcine gastric mucosa (2000 U·mL^−1^) at a ratio of 50:50 (*v*/*v*). After 120 min, pepsin was inactivated by increasing the pH to 7 by the addition of 2 M NaOH. Simulated intestinal fluid was then added (50:50 *v*/*v*): this contained trypsin (100 U·mL^−1^ of final mixture), chymotrypsin (25 U·mL^−1^ of final mixture), and total bile acids (10 mmol·mL^−1^ of final mixture). The concentration of bile salts in the bile was tested using a commercial kit (bile acid kit, ref 1 2212 99 90 313, DiaSys Diagnostic System GmbH, Germany) following the supplier’s protocol as described by Minekus et al. 2014 [24]. This test allows a 95–107% recovery of glycocholic, glycochenodeoxycholic, taurochenodeoxycholic, taurocholic, chenodeoxycholic, cholic, deoxycholic, taurodeoxycholic, and lithocholic acids. The digestion was carried out in a water bath (Grant, OLS 200, Cambridgeshire, England) at 37 °C under constant orbital stirring at 100 rpm. The simulated intestinal digested sample was labelled ID. For comparison, a control was prepared from the same WPH substrate; this was undigested. This was labelled WPH. ID and WPH samples were kept frozen (−20 °C) until further use or analysis.

### 2.4. Setting Up and Use of the UC

The ex vivo UC assays of WPH and ID samples were conducted following procedures set out by the French Ministry of Agriculture for animal research. In this pilot study, a 7-week-old piglet (Large white × Landrace × Duroc) was euthanized with ketamine/T61. After laparotomy, a 30-cm segment of the proximal jejunum, beginning at the ligament of Treitz was removed and placed in ice-cold Krebs (6.2 g NaCl, 0.32 g KCl, 0.15 g KH_2_PO4, 0.54 g MgSO_4_, 1.86 g NaHCO_3_, and 0.01 g CaCl_2_). Tissue was dissected to remove the muscle layers, and it was cut in 12 sections to be used in the UC (Physiologic Instrument, San Diego, CA, USA). The 1-cm^2^ tissue sections were exposed to 2.5 mL Krebs-mannitol (10 mM) and 2.5 mL Krebs-glucose (10 mM) solutions on the apical and basolateral sides of the membrane, respectively. Solutions were maintained at 39 °C, constantly stirred, and oxygenated with a gas mix of 95% O_2_–5% CO_2_. Two hundred microliters of the apical compartments were aliquoted, and this volume was replaced by prepared WPH and ID solutions in each of the 5 chambers, the other two chambers being used as blank controls, that is without WPH or ID addition. Samples (200 µL) were withdrawn at 10, 60, and 120 min and were immediately frozen ahead of further analysis. Fresh buffer solution was added each time to maintain a constant volume.

### 2.5. Tandem Mass Spectrometry

A nano-RSLC Dionex U3000 system fitted to a Q-Exactive mass spectrometer (Thermo Scientific, San Jose, CA, USA) coupled to a nanoelectrospray ion source was used for analysis of the peptides produced. 

UC aliquoted solutions were monitored with respect to the level of release of the amino groups throughout the experiment (data not shown), and the 3 samples with the highest concentrations were selected for MS analysis. Samples were diluted in acidic buffer (0.1% Trifluoroacetic acid (TFA) and 2% Acetonitrile) and filtered (0.45-µm cutoff) prior to the analysis. They were then concentrated on a µ-precolumn pepMap100 (C18 column (300 µm i.d. × 5 mm length, 5 µm particle size, 100 Å pore size; Dionex, Amsterdam, The Netherlands) and separated on a PepMap RSLC column (C18 column, 75 µm i.d. × 250 mm length, 3 µm particle size, 100 Å pore size; Dionex). Runs occurred at a flow rate of 0.3 µL·min^−1^ using a mixture of two solvents labelled A and B: solvent A (2% (*v*/*v*) acetonitrile, 0.08% (*v*/*v*) formic acid, and 0.01% (*v*/*v*) TFA) and solvent B (95% (*v*/*v*) acetonitrile, 0.08% (*v*/*v*) formic acid, and 0.01% (*v*/*v*) TFA). Peptides were eluted as follows: 5–35% B over 40 min, 85% B for 3 min, followed by column re-equilibration. The mass spectra were recorded in positive mode using the *m/z* range 250–2000. The resolution of the mass analyzer for an *m/z* value of 200 amu (atomic mass unit) was set with an acquisition of 70,000 for MS and 17,500 for MS/MS. For each MS scan, the ten most intense ions were selected for MS/MS fragmentation and were excluded from fragmentation for 20 s. 

X!TandemPipeline software [25] was used to identify the peptides present in the MS/MS spectra by reference to a prepared database composed of the major milk proteins plus values from the common Repository of Adventitious Proteins (http://thegpm.org/crap). The possible posttranslational modifications were serine or threonine phosphorylation, methionine oxidation, as well as the cyclic pyrolidone derivatives formed from N-terminal glutamine or glutamic acid. All peptides identified with an *e*-value below 0.05 were automatically validated. The peptide false discovery rate was below 0.6%. 

Each identified peptide was quantified using the MassChroQ software [26]. A **m/z** width of 10 ppm was used to perform extracted ion chromatograms (XIC) of peptides in time-aligned chromatograms: the area under the curve (AUC) was then quantified. When a peptide was found with several charge states, all of the component ion intensities were summed. Differences between samples were evaluated by the student’s *t*-test with a significance level of 5% (*p* < 0.05). Dairy peptides found in the control chambers were considered a background value, and their measured AUCs were subtracted from the other samples. 

The data and methods are available for open access at https://doi.org/10.15454/AIYGXO.

## 3. Results

**Figure 1:**
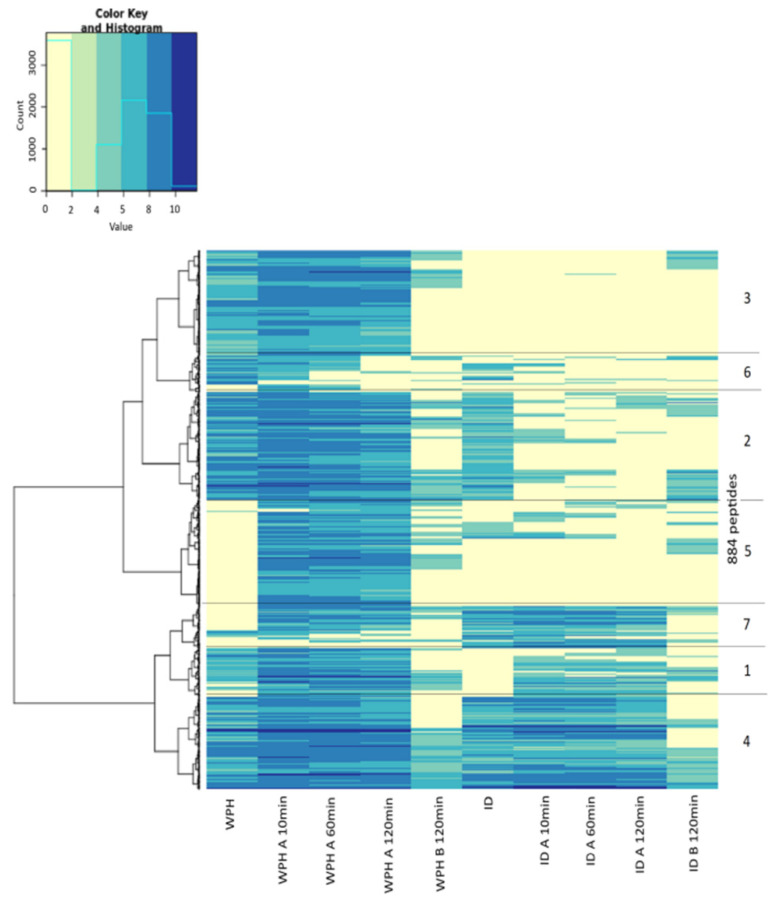
NanoLC–MS/MS heatmap showing all the peptides identified as a function of time in the Ussing chamber (UC) resulting from undigested (WPH) and previously digested (ID) whey protein hydrolysates: these are assembled into one of 7 groups based on similarity which reflects the protein source. Each horizontal line represents a peptide sequenced. Samples are distinguished by the letter A: apical compartment or B: basolateral compartment and sampling time (10 to 120 min).

The list of all the peptides identified during the experiment is given in the supplementary data. A total of 884 dairy peptides were identified from the studies with WPH and ID solutions: these were classified into 7 clusters based on similarity and are presented in Figure 1. This heatmap combines all the information collected by peptide analysis, and it will be discussed in parallel with further results. Peptides from 6 to 50 amino acids originated from 7 different proteins: three whey proteins (475 peptides from β-lactoglobulin, 51 from α-lactalbumin, and 48 from bovine serum albumin), three casein proteins (191 from β-casein, 22 from αs1-casein, and 57 from κ-casein), and glycosylation-dependent cell adhesion molecules (the remaining peptides). The commercial whey protein concentrate used to generate the hydrolysate had been previously reported to contain caseins [23]: therefore, it was not surprising to also find peptides arising from casein.

### 3.1. Gastrointestinal Digestion Causes a Decrease in the Number and MW of the Peptides

**Figure 2:**
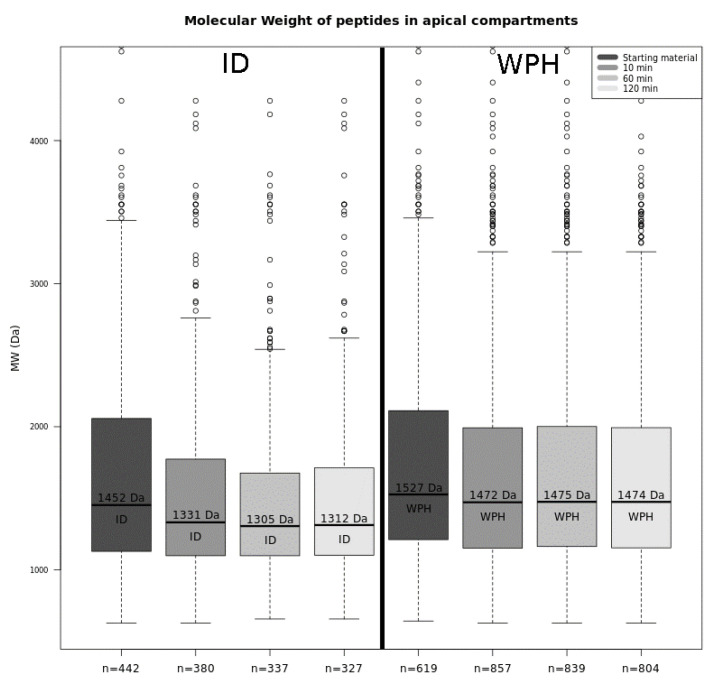
Median molecular weight and total number of peptides found in digested (ID) and undigested (WPH) whey hydrolysate following UC absorption in apical compartments progressing from 0 to 120 min.

Among all peptides found, 619 were related to the WPH solution and presented a median molecular weight (MW) of 1527 Da (Figure 2). The number of dairy peptides relating to the ID solution was fewer at 442. Their median MW was 1452 Da. Amongst these 442 ID peptides, 360 were also sequenced with WPH, whereas the other 82 were generated by the proteolytic activity of gastric and pancreatic enzymes during the simulated digestion, as they were not present in the WPH starting material. 

### 3.2. The Enzymatic Action of the Brush Border Membrane Modifies the Peptide Pattern in the Gut Lumen

The WPH solution was then exposed in the UC to segments of a piglet jejunum using the setup to simulate BBM hydrolysis and apical absorption of peptides. In the first 10 min of this experiment, an increase in the number of peptides from the WPH solution could be discerned, indicating an endopeptidase activity (Figure 2). 

The majority of the peptides released correspond to molecules that were more charged, with more basic and polar amino acids than the global peptide distribution in the source proteins (*p* < 0.05). During the latter stages of the experiment, the decrease in the number of milk-derived peptides identified was attributed to the fact that, when an aliquot is taken in the chamber for analysis, it is replaced by an identical volume of Krebs-mannitol and Krebs-glucose solutions in order to keep the volume at the chamber constant. This causes a progressive dilution of the sample in the UC. Although the MW of the peptides released seemed to decrease with time, no statistical significance was found (*p* > 0.05) to support this assertion.

In contrast to the results for the WPH, there was a progressive decrease in the median MW of dairy peptides in the apical compartment in the experiment with the ID substrate: values ranged from 1452 Da to 1331 Da (*p* < 0.05). This implies a BBM peptidase activity in the first 10 min of the test (Figure 2). Moreover, the number of dairy peptides identified fell from 442 to 380. Among the 380 peptides found, 91 were not found in the original ID sample, meaning they were probably produced by hydrolysis brought about by the peptidase in the BBM, while the other 289 peptides were present in the original ID. Eighty-one percent of all peptides found in the 10-min fraction were classified as cluster 1 (Figure 1), which contains molecules with significantly more aromatic amino acids than the proportions that exist in the source proteins (*p* < 0.05). As the MS analysis used only identifies peptides with more than 6 amino acid residues, the activity of the exopeptidases on the ID solution may have generated smaller peptide molecules and free amino acids, none of which were detected, thus explaining the observed apparent decrease in the number of peptides found for this sample. 

### 3.3. A Large Number of Peptides Has Been Identified in the Basolateral Side

**Figure 3:**
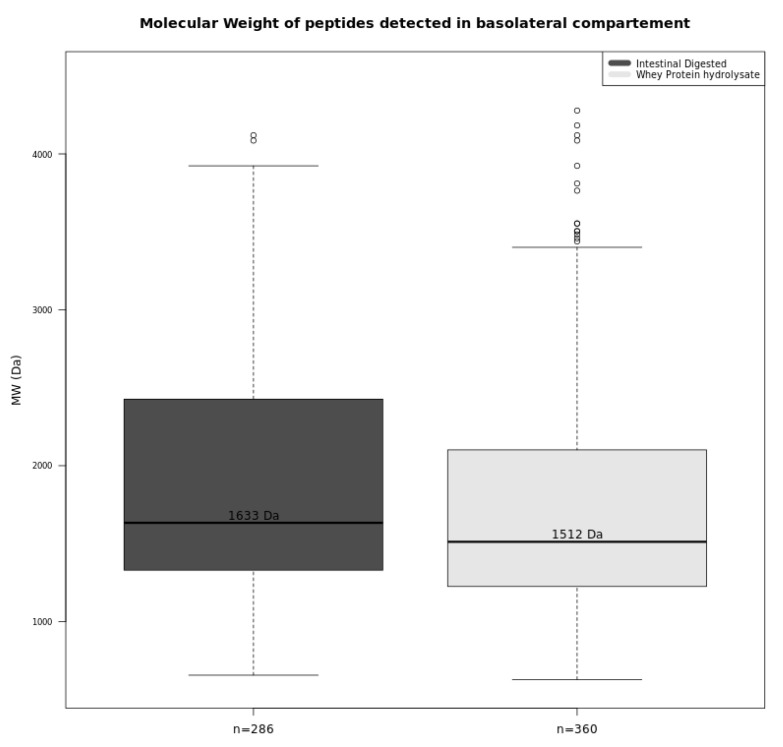
Median molecular weight and total number of peptides identified for undigested (WPH) and intestinal digested (ID) whey hydrolysate during the UC absorption study on basolateral compartments.

In total, 360 and 286 dairy peptides from the WPH and ID samples, respectively, were found in the basolateral compartment of the UC after 120 min (Figure 3). Interestingly, the median MW of these peptides was higher than that found on the apical side (*p* < 0.05), suggesting that oligopeptides can also be absorbed. However, it must be emphasized that the median MW only corresponds to the peptides that were detected with the mass spectrometry technique applied and that peptides shorter than 6 amino acids were not detected and not considered in the calculation of the median MW. The absorption of the smaller peptides (tri- and dipeptides) and free amino acids could not be quantified by the technique used. 

In general, peptides absorbed from WPH and ID samples were mostly those classified as clusters 4 and 2 (Figure 1). Peptides classified as cluster 2 exhibited significantly more aliphatic, hydrophobic, and nonpolar amino acids in their sequences (*p* < 0.05) when compared to the peptide distribution in the source protein, while small and acidic peptides were mostly associated with cluster 4. 

**Figure 4:**
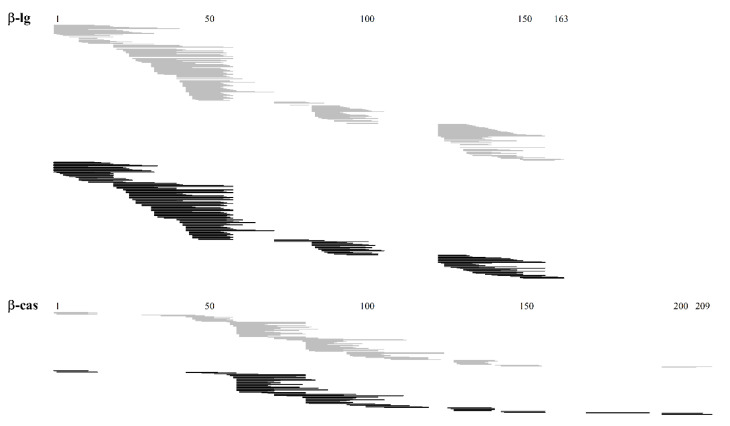
Peptides released from undigested (WPH) and previously intestinal digested (ID) whey protein hydrolysate identified by NanoLC-MS/MS in the basolateral compartment of the UC. Peptides found in the WPH and ID samples are represented by grey and black lines, respectively. Numbers represent the amino acid chain of each protein.

Among the absorbed peptides present in both WPH and ID samples, most were from β-lactoglobulin and β-casein. They displayed almost all sequences of such proteins and presented similar profiles, as shown in Figure 4.

### 3.4. Less than 3.35% of a Specific Peptide Can Pass through the Epithelial Barrier

**Figure 5:**
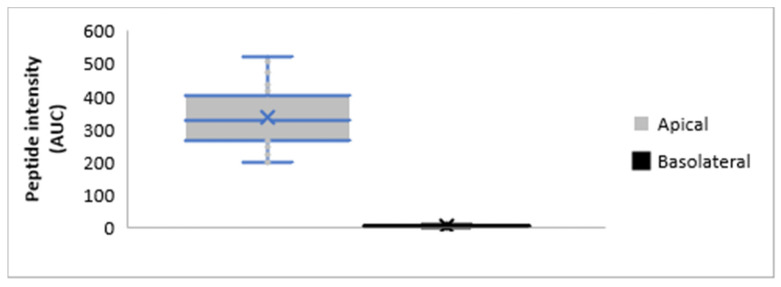
Median intensity (determined by the Area Under the Curve (AUC)) of the 147 peptides present in both the apical and basolateral compartments of the UC as determined by mass spectrometry.

Although there were 147 peptides common to both the apical and basolateral compartments after in vitro digestion, the concentration of these peptides in the basolateral side ranged from 0.60 to 3.35% (mean 1.70% ± 0.52) of that present in the apical compartment. This data confirms the selectivity of the intestinal barrier, and it can be observed in Figure 5. 

### 3.5. Biological Activity Relating to Absorbed Peptides

Amongst the absorbed peptides found, 10 β-casein fragments have been previously cited in relation to different bioactivities. Twenty-six peptides derived from β-lactoglobulin that were absorbed may have antioxidant and antimicrobial benefits as well as direct positive effects with respect to hypertension and diabetes and the improvement of memory function, as reported by many authors (Table 2). 

## 4. Discussion

These results based on simulations with a UC suggest that dietary peptides with a wide range of molecular weights can pass through the epithelial barrier and be absorbed via the small intestine: 360 and 286 dairy peptides from the WPH and ID samples were detected in the basolateral compartment of a UC setup with a piglet proximal jejunum segment to simulate human gut. A majority of these peptides derive from the hydrolysis of β-lactoglobulin and β-casein, and their molecular weights ranged from 656 Da to 4086 Da. Coupling an in vitro digestion model to an absorption study on UC represents a novel approach. Although still a simulation, this strategy creates new possibilities for investigating and understanding the mechanisms of nutrient absorption. 

Little is currently known about the absorption of peptides. While small peptide molecules can be absorbed by specific transporters such as PepT1, oligopeptides may only be absorbed via transcytosis and paracellular pathways, the latter being the main mechanism for the transport of unmodified peptides [39]. Molecular weight distribution and other characteristics such as hydrophobicity and charge distribution are all key factors that determine the likely transport routes for peptide absorption [40,41]. Previously, Chabance et al. [42] found two casein peptides in the blood of volunteers and suggested that their presence was the result of common transport pathways through the intestinal tissue, as they result from two major chymosin cleavage sites that are highly hydrophilic and located on the surface of milk micelles [42]. Another study [43] showed that the absorption of the peptide YQEPVLGPVRGPFPIIV (residue from β-casein 17-amino acid) through Caco-2 cells occurred mainly through transcytosis via internalized vesicles, although the paracellular transport via tight junctions could not be excluded. Finally, the bioavailability of 15 casein-derived oligopeptides in human plasma was confirmed after supplementation with Parmigiano Reggiano cheese for one week. However, the authors did not give detail of the possible mechanisms involved in the absorption of these peptides [44]. The mechanisms involved in the absorption of the Angiotensin-Converting Enzyme (ACE) inhibitory peptide VLPVPQK were investigated by Vij, Reddi, Kapila, and Kapila, who suggested [45] that it may have occurred via a SOPT2 or PepT1-like transporter. This peptide was also found in the present study amongst those present in the apical side of the WPH sample studies, but it was probably hydrolyzed by the BBM or intracellular peptidases, as it could no longer be found on the basolateral side of the chamber. However, even if many peptides have been shown to cross the epithelial barrier, the data obtained in the present study indicates that only a small proportion of a specific peptide will be thus absorbed. Indeed, no more than 3.35% of the any peptide present in the apical compartment of the chamber was found in the basolateral compartment, confirming the selectivity of the intestinal epithelium. This data confirms the bioavailability values of pharmaceutical peptide drugs that have been described recently that ranged between 1 and 2% [39,40]. 

Moreover, the present study provides evidence of the further hydrolysis by BBM peptidases of dietary peptides when they come in contact with the epithelial barrier. BBM peptidases are classified as either exo- or endopeptidases. The first shows affinity to either the C- or the N-terminal of an oligopeptide, generally producing single amino acids or dipeptides, whereas the latter cleaves nonterminal amino acids in oligopeptides of 30 or fewer amino acid residues [6]. In the present study, BBM peptidase activity occurred with both WPH and ID solutions, a notion supported by variation in the number of peptides sequenced as well as by the reduction of MW of these same peptides (Figure 2). BBM peptidase activity was also discussed by Picariello et al. [46], who reported that milk protein substrates were intensely hydrolyzed after incubation with these enzymes. The authors observed that milk-derived peptides resistant to gastric and intestinal enzymes could nonetheless be further hydrolyzed by BBM peptidases, representing a final step of degradation.

Amongst the peptides that were found either in the apical or in the basolateral compartments of the UC, some are known to stimulate certain biological activities. Milk proteins are particularly well-known for being a source of such peptides, which can exert their influence either in the gut lumen or in different specific organs. These protein fragments may play an important role in gut health promotion, stimulating mucus formation; preventing gastrointestinal barrier-related disorders; and strengthening the barrier function of the intestine against toxins, bacteria, self-digestion, and physical abrasions [47,48]. Previous studies [47,49] also showed that β-lactoglobulin and β-casein hydrolysates were able to stimulate mucins’ secretion which was demonstrated in vitro and in vivo. Some of these peptides were also found in the apical compartment of the UC containing WPH or ID samples. Hence, it might be suggested that there is a possible indirect influence of WPH and ID dairy peptides in the formation of the mucus gel layer cover, which needs to be further investigated. Despite the lack of broad scientific agreement with respect to the bioavailability and bioactivity of certain peptides relating to their susceptibility to plasma soluble peptidases and the high concentrations required to produce these effects [50,51], some in vivo studies do support the idea of a biological impact of dairy peptides on the intestinal barrier and in different parts of the body after being absorbed [52,53,54]. In any case, the concentration needed to obtain biological effects and the mechanisms involved in the transepithelial permeation process needs to be further investigated. Therefore, despite the limited number of replicates, this pilot study may be considered a new evidence with respect to the absorption of oligopeptides: it may also be a starting point for better understanding of dairy peptides and their related bioavailability and bioactivity.

This present study reveals that whey protein hydrolysate is relatively stable under conditions of digestion and is barely degraded by the digestive enzymes. Although 82 peptides were released during gastrointestinal digestion, most of the peptides found in the ID solution were also present in the WPH. These peptides could have either resisted digestion or been the products of new enzymatic cleavages occurring during the simulated digestion. Picariello et al. [46] and Sanchon et al. [55] both previously discussed the resistance of some milk-derived peptides to gastrointestinal digestion and associated this to possible biological benefits after absorption.

The present study also served to determine if UC, when coupled to an in vitro digestion model, could be a useful tool to investigate dietary peptide digestion and absorption by the epithelial barrier and if it can deliver a useful and relevant simulation. This is a model approach that has some limits but also many possibilities for refinement. The in vitro digestion model used (i.e. based on the international INFOGEST consensus) follows all the dilution steps that the food will experience in the different compartments of the gut. Therefore, the peptide concentration found in the UC may be expected to simulate what occurs in vivo. However, it has to be admitted that the conditions for coupling an in vitro digestion with absorption models are currently being reviewed by the INFOGEST international network on food digestion with the objective of reaching a consensus and thus proposing guidelines to perform new experiments in the near future. In this respect, the present study, which couples an in vitro digestion model with a UC, will therefore provide relevant data that will be useful for establishing the best conditions of experiment.

The focus of this work was to use only samples collected at the end of in vitro gastrointestinal digestion and to study their absorption. However, it is realized that the digestive process is dynamic, and it would have been interesting to study the kinetics of peptide absorption throughout the whole process rather than the absorption of a sample taken at the end of gastrointestinal digestion. The pattern of peptides crossing the epithelial barrier after only a few minutes of digestion might well be different to that obtained after several hours of digestion. A logical development of this work will be to combine the dynamic in vitro digestion of foods with absorption studies based on UC to bring the simulation closer to the physiological reality.

## 5. Conclusions

There is a fast action of brush border exo- and endopeptidases on large peptide fragments from WPH and ID whey protein hydrolysates. Some of the peptides found in the apical portion of the chambers may also offer potential beneficial bioactivity with respect to the intestinal epithelium, although this claim requires further investigation. Several oligopeptides were absorbed through the intestinal epithelium, most of them relating to hydrophobic, aromatic, and acidic amino acids, indicating a preference for absorption of this type of molecule. Such results challenge the common viewpoint relating to the exclusive absorption of amino acids, dipeptides, and tripeptides. It reinforces new evidence relating to the bioavailability of dairy oligopeptides and suggests their probable beneficial bioactivity. Inevitably, further research is required to deepen the understanding on the presented data and its implications and to investigate the likely mechanisms involved in the intestinal transepithelial passage of such peptides. 

## Figures and Tables

**Table 1:** Composition of the simulated gastric (SGF) and simulated intestinal (SIF) fluids.

Reagent	SGF (mM)	SIF (mM)
KCl	6.9	6.8
KH_2_PO_4_	0.9	0.8
NaHCO_3_	25	85
NaCl	47.2	38.4
MgCl_2_(H_2_O)_6_	0.12	0.33
(NH_4_)_2_CO_3_	0.5	-
HCl	15.6	8.4
CaCl_2_(H_2_O)_2_	0.15	0.6

**Table 2:** Health benefits reported in the literature for peptides released from undigested (WPH) and intestinal digested (ID) whey protein hydrolysates following simulated absorption studies in a UC: the proteins considered are β-lactoglobulin (β-lg), α-lactalbumin (α-la), β-casein (β-cas), κ-casein (κ-cas), and α_S1_-casein (α-cas_S1_)_._

Protein	Peptide	WPH	ID	Bioactivity	Reference
**β-lg**	KGLDIQKVAGTW	X	X	ACE Inhibitory	[27]
	GLDIQK	X	X		[28]
	VLDTDYK		X		
	IVTQTMKG		X		[29]
	VLDTDYKK		X		
	KTKIPAV	X	X		
	VEELKPTPEGDLE	X			
	LEKFDK	X			
	LDIQKVAGTW	X			[30]
	RELKDLKGYGG	X	X	Anti-diabetic	[31]
	LIVTQTMKGLD		X		
	IVTQTMKGLD	X			
	IVTQTMKGLDIQ		X		
	LKPTPEGDL	X	X	DPP-IV inhibitory	[30]
	TPEVDDEALEK	X	X		
	VLVLDTDYK	X	X		
	LVLDTDYK		X	Antimicrobial	[32]
	ALKALPMHI	X			[33]
	GLDIQKVAGT	X	X		[30]
	TPEVDDEALEK	X	X		
	VLVLDTDYK	X	X		
	IDALNENK	X	X		
	SLAMAASDISLL	X	X		
	KPTPEGDLEI	X		Memory function	[34]
	KTKIPAVF	X		Antioxidant	[35]
	NENKVLVLDTDYKKY	X			[36]
**α-la**	GGVSLPEW	X		ACE Inhibitory	[37]
	DKVGINYW	X			[30]
	LKGYGGVSLPEW		X		
**β-cas**	LVYPFPGPIPN	X	X		[5]
	VYPFPGPIPN	X	X		[30]
	YPFPGPIPN	X	X		
	YPFPGPIHNSLPQ	X			
	VENLHLPLPL	X		Anticancer	[38]
	VYPFPGPIPN	X	X	Antioxidant	[30]
	YPFPGPIPN	X	X	DPP-IV inhibitory	
	VYPFPGPIP	X	X	Prolyl endopeptidase-inhibitory	
	PVVVPPFLQPE	X	X	Antimicrobial	
**κ-cas**	VQVTSTAV	X		Antimicrobial	
**α-cas_S1_**	SDIPNPIGSENSEK	X	X		

## Supplementary Materials

The following are available online at https://www.mdpi.com/2304-8158/9/10/1415/s1.
