# Supplementary material for: The Influence of Peptidases in Intestinal Brush Border Membranes on the Absorption of Oligopeptides from Whey Protein Hydrolysate: An Ex Vivo Study Using an Ussing Chamber
Source: Foods. 2020 Oct 7;9(10):1415. doi: 10.3390/foods9101415 (PMC7601095; doi:10.3390/foods9101415)
Supplement: Supplementary file 1 [file foods-09-01415-s001.pdf]

Supplementary data

List of Peptides found in WPH, WPH A 10-120 min, WPH B 120 min, ID, ID A 10-120 min and ID B 120 min

WPH

AASDISLLDAQ
AASDISLLDAQSAPL
AASDISLLDAQSAPLRV
AASDISLLDAQSAPLRVY
AASDISLLDAQSAPLRVYVE
AASDISLLDAQSAPLRVYVEE
AASDISLLDAQSAPLRVYVEELKPTPEGD
AASDISLLDAQSAPLRVYVEELKPTPEGDL
AASDISLLDAQSAPLRVYVEELKPTPEGDLE
AASDISLLDAQSAPLRVYVEELKPTPEGDLEI
AASDISLLDAQSAPLRVYVEELKPTPEGDLEIL
AASDISLLDAQSAPLRVYVEQLKPTPEGDL
AASDISLLDAQSAPLRVYVEQLKPTPEGDLEIL
AIPENLPPLTAD
AIPPKKNQD
AIPPKKNQDKTEIPTINT
ALEKFDKA
ALEKFDKALK
ALEKFDKALKA
ALEKFDKALKALPM
ALEKFDKALKALPMH
ALEKFDKALKALPMHI
ALEKFDKALKALPMHIR
ALEKFDKALKALPMHIRL
ALEKFDKALKALPMHIRLSFNPTQL
ALKALPMHI
ALKALPMHIRL
ALNENKVLVL
ALNENKVLVLDTDYKKYL
ALNENKVLVLDTDYKKYLL
ALNENKVLVLDTDYKKYLLF
ALPMHI
ALPMHIRL
AMAASDISLL
AMAASDISLLDAQSAPL
AMAASDISLLDAQSAPLRV
AMAASDISLLDAQSAPLRVYVEELKPTPEGDL
AMAASDISLLDAQSAPLRVYVEELKPTPEGDLEIL
AQSAPLRVYVEELKPTPEGDL
AQSAPLRVYVEELKPTPEGDLE

AQSAPLRVYVEELKPTPEGDLEIL
ASDISLLDAQSAPLR
ASDISLLDAQSAPLRV
ASDISLLDAQSAPLRVYVEELKPTPEGD
ASDISLLDAQSAPLRVYVEELKPTPEGDLEIL
ASPEVIESPPEINT
DAFLGSFLYEYSR
DALNENKVLVLDTDY
DAQSAPLRV
DAQSAPLRVY
DAQSAPLRVYVE
DAQSAPLRVYVEE
DAQSAPLRVYVEELKPTPEGD
DAQSAPLRVYVEELKPTPEGDL
DAQSAPLRVYVEELKPTPEGDLE
DAQSAPLRVYVEELKPTPEGDLEI
DAQSAPLRVYVEELKPTPEGDLEIL
DAQSAPLRVYVEQLKPTPEGD
DAQSAPLRVYVEQLKPTPEGDL
DDEALEKFDKALKALPMHIRL
DEALEKFDK
DEALEKFDKALKALPMHIRL
DEALEKFDKALKALPMHIRLSFNPTQL
DELQDKIHPFA
DELQDKIHPFAQT
DELQDKIHPFAQTQS
DELQDKIHPFAQTQSL
DISLLDAQSAPLRV
DKIHPFAQT
DKIHPFAQTQS
DKIHPFAQTQSL
DKLKHLVDEPQNL
DKTEIPTINT
DKVGINY
DKVGINYW
DLKGYGGVSL
DLKGYGGVSLPEWV
DLSKEPSISRE
DLSKEPSISREDL
DSPEVIESPPEINT
DTDYKKYL
DTDYKKYLL
DTDYKKYLLF
DTHKSEIAHRFKDLGEEHFKG
DTHKSEIAHRFKDLGEEHFKGLVL
DVENLHLPLP

EALEKFDKA
EALEKFDKALK
EALEKFDKALKALPM
EALEKFDKALKALPMHI
EALEKFDKALKALPMHIR
EALEKFDKALKALPMHIRL
EALEKFDKALKALPMHIRLSFNPTQL
EAMAPKHKEMPFPKYPVEPFTESQS
EAQPTDASAQF
EASPEVIESPPEIN
EDSPEVIEGPPEINT
EDSPEVIESPPEINT
EELKPTPEGDL
EELKPTPEGDLE
EELKPTPEGDLEI
EELKPTPEGDLEIL
EKFDKAL
EKFDKALKA
EKFDKALKALP
EKFDKALKALPM
EKFDKALKALPMH
EKFDKALKALPMHI
EKFDKALKALPMHIR
EKFDKALKALPMHIRL
EKTKIPAVF
ELKDLKGYGGVS
ELKDLKGYGGVSL
ELKDLKGYGGVSLPEWV
ELKPTPEGD
ELKPTPEGDL
ELKPTPEGDLE
ELKPTPEGDLEIL
ELQDKIHPF
ELQDKIHPFAQTQSL
EMPFPKYPVE
EMPFPKYPVEPFTESQS
ENSAEPEQSLV
EPVLGPVRGPFPIIV
EQIVIR
ESPPEINT
EVIESPPEINT
FDKALKALPMHI
FDKLKHLVDEPQNL
FEVVKT
FFVAPFPEVF
FKDLGEEHFKGLV

FKIDALNE
FMAIPPKKNQDKTEIPTINT
FPGPIPNSLPQNIPPLTQT
FPPQSVL
FQINNKIW
FQINNKIWCKDDQNPHSSNICNISCDKFLDDDLTDDIMCVKKILDKVGIN
FVAPFPEV
FVAPFPEVFG
FVAPFPEVFGK
FVAPFPEVFGKE
FVAPFPEVFGKEKVNE
FVAPFPEVFGKEKVNEL
GGVSLPEW
GLDIQKVAGTW
GLDIQKVAGTWY
GLDIQKVAGTWYSLA
GLDIQKVAGTWYSLAMA
GVSKVKEAM
GVSKVKEAMAP
GVSKVKEAMAPK
GVSKVKEAMAPKH
GVSKVKEAMAPKHK
GVSKVKEAMAPKHKEMPFPK
GVSKVKEAMAPKHKEMPFPKYPVE
GVSKVKEAMAPKHKEMPFPKYPVEPF
GVSKVKEAMAPKHKEMPFPKYPVEPFT
GVSKVKEAMAPKHKEMPFPKYPVEPFTES
GVSKVKEAMAPKHKEMPFPKYPVEPFTESQ
GVSKVKEAMAPKHKEMPFPKYPVEPFTESQS
GVSKVKEAMAPKHKEMPFPKYPVEPFTESQSL
GVSKVKEAMAPKHKEMPFPKYPVEPFTESQSLT
GVSKVKEAMAPKHKEMPFPKYPVQPFTESQS
HIQKEDVPSE
HIQKEDVPSERYLG
HIRLSFNPT
HIRLSFNPTQ
HIRLSFNPTQL
HKEMPFPKYPVE
HKEMPFPKYPVEPF
HKEMPFPKYPVEPFTES
HKEMPFPKYPVEPFTESQS
HLVDEPQNL
HQGLPQPFPEV
HQPHQPLPPTVM
HVKLVNE
IASGEPTSTPITE

IASGEPTSTPTIE
IASGEPTSTPTIEA
IASGEPTSTPTTE
IASGEPTSTPTTEA
ICNISCDKFLDDDLTDDIMCVKKILDKVGINY
IDALNENKVLV
IDALNENKVLVLDTDYK
IDALNENKVLVLDTDYKK
IESPPEINT
IHPFAQTQS
IHPFAQTQSL
IIAEKTKIPA
IIAEKTKIPAVF
IIVTQTMKG
IIVTQTMKGL
IIVTQTMKGLDI
IIVTQTMKGLDIQ
IIVTQTMKGLDIQKVA
IIVTQTMKGLDIQKVAGT
IIVTQTMKGLDIQKVAGTW
IIVTQTMKGLDIQKVAGTWYSL
IIVTQTMKGLDIQKVAGTWYSLAM
IIVTQTMKGLDIQKVAGTWYSLAMAA
IIVTQTMKGLDIQKVAGTWYSLAMAASD
IIVTQTMKGLDIQKVAGTWYSLAMAASDISLLD
IIVTQTMKGLDIQKVAGTWYSLAMAASDISLLDAQSAPLR
ILDKVGINY
ILNKPEDET
ILNKPEDETHL
IMCVKKILDKVGINY
IPPKKNQDKTEIPTINT
IQKVAGTW
IQKVAGTWYSL
IQKVAGTWYSLA
IQKVAGTWYSLAM
IRNLQISNE
IRNLQISNEDL
ISKEQIVIR
ISKEQIVIRSS
ISLLDAQSAPLR
ISLLDAQSAPLRV
ISLLDAQSAPLRVY
ISLLDAQSAPLRVYVE
ISLLDAQSAPLRVYVEELKPTPEGD
ISLLDAQSAPLRVYVEELKPTPEGDL
ISLLDAQSAPLRVYVEELKPTPEGDLE

ISLLDAQSAPLRVYVEELKPTPEGDLEIL
IVQNNDSTEY
IVQNNDSTEYGL
IVRYTRKVPQVSTPT
IVRYTRKVPQVSTPTL
IVTQTMKG
IVTQTMKGL
IVTQTMKGLD
IVTQTMKGLDIQ
IVTQTMKGLDIQKVAGT
IVTQTMKGLDIQKVAGTW
IVTQTMKGLDIQKVAGTWYSL
IVTQTMKGLDIQKVAGTWYSLA
KADEKKFWG
KADEKKFWGKY
KALKALPM
KALKALPMHIRL
KALKALPMHIRLSFNPTQL
KALPMHIRL
KDLKGYGGVSL
KFDKALKALPMHI
KFDKALKALPMHIRL
KFLDDDL
KFLDDDLTDD
KGLDIQKVAGTW
KIDALNE
KIDALNEN
KIDALNENK
KIDALNENKV
KIDALNENKVL
KIDALNENKVLV
KIDALNENKVLVL
KIDALNENKVLVLD
KIDALNENKVLVLDT
KIDALNENKVLVLDTD
KIDALNENKVLVLDTDY
KIDALNENKVLVLDTDYK
KIDALNENKVLVLDTDYKK
KIDALNENKVLVLDTDYKKY
KIDALNENKVLVLDTDYKKYL
KIDALNENKVLVLDTDYKKYLL
KIDALNENKVLVLDTDYKKYLLF
KIDALNENKVLVLDTDYKKYLLFCMENSAEPEQSLVCQ
KIDALNENKVLVLDTDYKKYLLFCMENSAEPEQSLVCQCL
KIEKFQSEEQQQTEDEL
KIEKFQSEEQQQTEDELQDKIHPFAQTQSL

KIHPFAQT
KIHPFAQTQ
KIHPFAQTQS
KIHPFAQTQSL
KILDKVGINY
KNQDKTEIPTINT
KPTPEGDLE
KPTPEGDLEI
KPTPEGDLEIL
KPTPEGDLEILL
KTEIPTINT
KTKIPAV
KTKIPAVF
LDAQSAPLR
LDAQSAPLRV
LDAQSAPLRVY
LDAQSAPLRVYVE
LDAQSAPLRVYVEE
LDAQSAPLRVYVEELKPTPEGD
LDAQSAPLRVYVEELKPTPEGDL
LDAQSAPLRVYVEELKPTPEGDLE
LDAQSAPLRVYVEELKPTPEGDLEIL
LDAQSAPLRVYVEQLKPTPEGDLEIL
LDIQKVAGT
LDIQKVAGTW
LEDSPEVIESPPEINT
LEKFDKA
LEKFDKALKALPM
LEKFDKALKALPMH
LEKFDKALKALPMHI
LEKFDKALKALPMHIRL
LEKFDKALKALPMHIRLSFNPTQL
LIVTQTMK
LIVTQTMKGL
LIVTQTMKGLD
LIVTQTMKGLDI
LIVTQTMKGLDIQK
LIVTQTMKGLDIQKVAG
LIVTQTMKGLDIQKVAGTWY
LIVTQTMKGLDIQKVAGTWYS
LIVTQTMKGLDIQKVAGTWYSL
LIVTQTMKGLDIQKVAGTWYSLA
LIVTQTMKGLDIQKVAGTWYSLAM
LIVTQTMKGLDIQKVAGTWYSLAMA
LIVTQTMKGLDIQKVAGTWYSLAMAASD
LIVTQTMKGLDIQKVAGTWYSLAMAASDISL

LIVTQTMKGLDIQKVAGTWYSLAMAASDISLL
LKALPMHIRL
LKDLKGY
LKDLKGYGGVS
LKDLKGYGGVSL
LKGYGGVSLPEW
LKPTPEGDL
LKPTPEGDLE
LKPTPEGDLEIL
LLDAQSAPLRV
LNENKVLVLDTDYKKYLL
LNKPEDETHL
LPKLKPD
LPMHIRL
LPQNIPPLTQT
LQDKIHPF
LQDKIHPFAQ
LQDKIHPFAQT
LQDKIHPFAQTQ
LQDKIHPFAQTQS
LQDKIHPFAQTQSL
LRLAKEYEAT
LRLKKYKVPQL
LSFNPTQLEEQCHI
LSQKFPKAEF
LTDVENLHLPLPLL
LVLDTDYKKYL
LVLDTDYKKYLL
LVNELTEFAK
LVRTPEVDDEALEKFDKALKALPMHI
LVYPFPGPIHNSLPQNIPPLTQT
LVYPFPGPIPN
LVYPFPGPIPNSL
LVYPFPGPIPNSLPQNIPPLTQT
LYQEPVLGPVRGPFPIIV
LYYANKYNGVF
MAASDISLL
MAASDISLLDAQSAPL
MAASDISLLDAQSAPLRV
MAASDISLLDAQSAPLRVY
MAASDISLLDAQSAPLRVYVE
MAASDISLLDAQSAPLRVYVEELKPTPEGDL
MAASDISLLDAQSAPLRVYVEELKPTPEGDLE
MAASDISLLDAQSAPLRVYVEELKPTPEGDLEIL
MAIPPKKNQD
MAIPPKKNQDKTEIPT

MAIPPKKNQDKTEIPTIN
MAIPPKKNQDKTEIPTINT
MELGHKIM
MELGHKIMRNLEN
MELGHKIMRNLENT
MELGHKIMRNLENTVKE
MELGHKIMRNLENTVKETIK
MELGHKIMRNLENTVKETIKY
MELGHKIMRNLENTVKETIKYLKS
MELGHKIMRNLENTVKETIKYLKSL
MENSAEPEQSL
MGVSKVKEAMAPK
MHQPHQPLPPT
MHQPHQPLPPTVM
NENKVLVL
NENKVLVLD
NENKVLVLDTDYKKY
NENKVLVLDTDYKKYL
NENKVLVLDTDYKKYLL
NKPEDETHL
NKVLVLDTDY
NKVLVLDTDYKKYL
NKVLVLDTDYKKYLL
NLENTVKET
NQDKTEIPTINT
NSAEPEQSL
NSAEPEQSLA
PEVIESPPEINT
PMHIRL
PPKKNQDKTEIPTINT
PPKKNQDKTEIPTINTI
PPKKNQDKTEIPTINTIA
PPLTQTPVVVPPFLQPEVMGVSKVKEAMAPKHKEMPFP
PQNIPPLTQT
PQNIPPLTQTPVVVPPFIQPEV
PVLGPVRGPFPIIV
PVVVPPF
PVVVPPFIQPEVMG
PVVVPPFIQPEVMGV
PVVVPPFIQPEVMGVSK
PVVVPPFIQPEVMGVSKVK
PVVVPPFIQPEVMGVSKVKEAMAPKHK
PVVVPPFL
PVVVPPFLQ
PVVVPPFLQP
PVVVPPFLQPE

PVVVPPFLQPEV
PVVVPPFLQPEVM
PVVVPPFLQPEVMG
PVVVPPFLQPEVMGV
PVVVPPFLQPEVMGVS
PVVVPPFLQPEVMGVSKV
PVVVPPFLQPEVMGVSKVKE
PVVVPPFLQPEVMGVSKVKEAMAPK
PVVVPPFLQPEVMGVSKVKEAMAPKH
QDKIHPF
QDKIHPFA
QDKIHPFAQT
QDKIHPFAQTQ
QDKIHPFAQTQS
QDKIHPFAQTQSL
QDKTEIPTINT
QEPVLGPVRGPFPIIV
QEPVLGPVRGPFPILV
QINNKIWCKDDQNPHSSNICNISCDKFLDDDLTDDIMCVKKILDKVGINY
QLKPTPEGDL
QPQSQNPKLPL
QPQSQNPKLPLSIL
QSAPLRV
QSLVYPFPGPIHNSLPQNIPPLTQT
QSLVYPFPGPIPNSLPQNIPPLTQT
QSWMHQPHQPLPPT
QSWMHQPHQPLPPTVM
QTMKGLDIQKVAGTW
RELEELNVPG
RELEELNVPGE
RELEELNVPGEI
RELEELNVPGEIVE
RELKDLKG
RELKDLKGY
RELKDLKGYGG
RELKDLKGYGGVS
RELKDLKGYGGVSL
RELKDLKGYGGVSLPE
RELKDLKGYGGVSLPEW
RPKHPIKHQGLPQEVL
RPKHPIKHQGLPQEVLNE
RPKHPIKHQGLPQPFPEV
RTPEVDDEALEKFDK
RTPEVDDEALEKFDKA
RVYVEELKPTPEGD
RVYVEELKPTPEGDL

RVYVEELKPTPEGDLE
RVYVEELKPTPEGDLEI
RVYVEELKPTPEGDLEIL
RVYVEELKPTPEGDLEILLQK
SAEPEQSL
SAPLRV
SAPLRVYVEELKPTPEGDL
SAPLRVYVEELKPTPEGDLEIL
SDIPNPIGSENSEKTTM
SDIPNPIGSENSEKTTMPL
SDISLLDAQSAPL
SDISLLDAQSAPLRV
SDISLLDAQSAPLRVY
SDISLLDAQSAPLRVYVEELKPTPEGD
SDISLLDAQSAPLRVYVEELKPTPEGDL
SDISLLDAQSAPLRVYVEELKPTPEGDLE
SEEQQQTEDELQDK
SFNPTQL
SFNPTQLEEQCHI
SGEPTSTPTIE
SGEPTSTPTIEA
SKEPSISREDL
SKVKEAMAPK
SLAMAASDISL
SLAMAASDISLL
SLAMAASDISLLDAQSAPLRV
SLAMAASDISLLDAQSAPLRVY
SLLDAQSAPLRV
SLPQNIPPLTQT
SLSQSKVLPVPEKAVPYPQ
SLSQSKVLPVPEKAVPYPQRD
SLSQSKVLPVPEKAVPYPQRDMPIQ
SLSQSKVLPVPEKAVPYPQRDMPIQA
SLSQSKVLPVPQ
SLSQSKVLPVPQK
SLSQSKVLPVPQKAVPYPQ
SLSQSKVLPVPQKAVPYPQRD
SLSQSKVLPVPQKAVPYPQRDMPIQ
SLSQSKVLPVPQKAVPYPQRDMPIQA
SLSQSKVLPVPQKAVPYPQRDMPIQAF
SLVYPFPGPIHNSLPQNIPPLTQT
SLVYPFPGPIPNSLPQNIPPLTQT
SPEVIEGPPEINT
SPEVIESPPEINT
SPPEINT
SQKFPKAEF

SRQPQSQNPKLPLSIL
SSRQPQSQNPKLPL
SSRQPQSQNPKLPLSI
SSRQPQSQNPKLPLSIL
TDVENLHLPLP
TDVENLHLPLPL
TDVENLHLPLPLL
TDVENLHLPLPLLQ
TDVENLHLPPL
TDYKKYL
TDYKKYLL
TEDELQDKIHPF
TEIPTIN
TEIPTINT
TFHTSGYDTQA
TIASGEPTSTPTIE
TIASGEPTSTPTTE
TIKYLKSL
TIKYLKSLFSHA
TLEDSPEVIESPPEINT
TMKGLDIQKVAGTW
TPEVDDEALEK
TPEVDDEALEKF
TPEVDDEALEKFDKALKALPMHI
TQTMKGLDIQKVAGTW
TVKETIKYLKS
TVKETIKYLKSL
TVKETIKYLKSLFSHA
VATLEDSPEVIESPPEINT
VDDEALEKFDK
VEELKPTPEG
VEELKPTPEGD
VEELKPTPEGDL
VEELKPTPEGDLE
VEELKPTPEGDLEIL
VEELKPTPEGDLEILL
VEELKPTPEGDLEILLQKWEND
VEELKPTPEGDLEILLQKWENDECAQKK
VEKDAIPENLPPLTADFAEDKD
VELLKHKPKATEEQ
VELLKHKPKATEEQL
VELLKHKPKATEEQLKT
VELLKHKPKATEEQLKTVM
VENLHLPLPLL
VLDTDYKKYL
VLDTDYKKYLL

VLDTDYKKYLLF
VPPFLQPEVM
VQVTSTAV
VRTPEVDD
VRTPEVDDE
VRTPEVDDEA
VRTPEVDDEAL
VRTPEVDDEALE
VRTPEVDDEALEKF
VRTPEVDDEALEKFD
VRTPEVDDEALEKFDK
VRTPEVDDEALEKFDKA
VRTPEVDDEALEKFDKAL
VRTPEVDDEALEKFDKALK
VRTPEVDDEALEKFDKALKA
VRTPEVDDEALEKFDKALKALP
VRTPEVDDEALEKFDKALKALPM
VRTPEVDDEALEKFDKALKALPMH
VRTPEVDDEALEKFDKALKALPMHI
VRTPEVDDEALEKFDKALKALPMHIR
VRTPEVDDEALEKFDKALKALPMHIRL
VRTPEVDDEALEKFDKALKALPMHIRLS
VRTPEVDDEALEKFDKALKALPMHIRLSFNPT
VRTPEVDDEALEKFDKALKALPMHIRLSFNPTQ
VRTPEVDDEALEKFDKALKALPMHIRLSFNPTQL
VRTPEVDDEALEKFDKALKALPMHIRLSFNPTQLEEQCHI
VSKVKEAMAPK
VSKVKEAMAPKH
VSKVKEAMAPKHK
VTQTMKGLDIQKVAGTW
VYPFPGPIHN
VYPFPGPIHNSL
VYPFPGPIHNSLPQ
VYPFPGPIHNSLPQNIPPLT
VYPFPGPIHNSLPQNIPPLTQT
VYPFPGPIP
VYPFPGPIPN
VYPFPGPIPNSL
VYPFPGPIPNSLPQ
VYPFPGPIPNSLPQNIPPLT
VYPFPGPIPNSLPQNIPPLTQ
VYPFPGPIPNSLPQNIPPLTQT
VYPFPGPIPNSLPQNIPPLTQTPV
VYVEELKPTPEGDLEI
WLAHKALCSEKLDQ
WMHQPHQPLPPTVM

WSVARLSQKFPKAEF
YKKYLLF
YPFPGPIHNSLPQ
YPFPGPIPN
YPFPGPIPNSLPQNIPPLTQT
YQEPVLGPVRGPFPI
YQEPVLGPVRGPFPILV
YSLAMAASDISL
YSLAMAASDISLL
YSLAMAASDISLLDAQSAPL
YSLAMAASDISLLDAQSAPLR
YSLAMAASDISLLDAQSAPLRV
YSLAMAASDISLLDAQSAPLRVYVEELKPTPEGDL
YSLAMAASDISLLDAQSAPLRVYVEELKPTPEGDLEIL
YVEELKPTPE
YVEELKPTPEG
YVEELKPTPEGD
YVEELKPTPEGDL
YVEELKPTPEGDLE
YVEELKPTPEGDLEI
YVEELKPTPEGDLEIL
YVEELKPTPEGDLEILL
YVEELKPTPEGDLEILLQK
YVEQLKPTPEGDL
YVPKAFDEKL
YYANKYNGV
YYANKYNGVF

WPH.A.10min
AASDISLLDAQ
AASDISLLDAQSAPL
AASDISLLDAQSAPLRV
AASDISLLDAQSAPLRVY
AASDISLLDAQSAPLRVYVE
AASDISLLDAQSAPLRVYVEE
AASDISLLDAQSAPLRVYVEELKPTPEGD
AASDISLLDAQSAPLRVYVEELKPTPEGDL
AASDISLLDAQSAPLRVYVEELKPTPEGDLE
AASDISLLDAQSAPLRVYVEELKPTPEGDLEI
AASDISLLDAQSAPLRVYVEELKPTPEGDLEIL
AASDISLLDAQSAPLRVYVEQLKPTPEGDL
AASDISLLDAQSAPLRVYVEQLKPTPEGDLEIL
ADEKKFWG
AEKTKIPAV
AIPENLPPLTAD

AIPPKKNQD
AIPPKKNQDKTEIPTINT
AIVQNNDSTEYG
ALEKFDK
ALEKFDKA
ALEKFDKAL
ALEKFDKALK
ALEKFDKALKA
ALEKFDKALKALPM
ALEKFDKALKALPMH
ALEKFDKALKALPMHI
ALEKFDKALKALPMHIR
ALEKFDKALKALPMHIRL
ALEKFDKALKALPMHIRLSFNPTQL
ALKALPMHI
ALKALPMHIR
ALKALPMHIRL
ALNENKVLVL
ALNENKVLVLDTDY
ALNENKVLVLDTDYK
ALNENKVLVLDTDYKK
ALNENKVLVLDTDYKKYL
ALNENKVLVLDTDYKKYLL
ALPMHI
ALPMHIRL
ALPMHIRLSFNPT
ALPMHIRLSFNPTQL
AMAASDISLL
AMAASDISLLDAQSAPL
AMAASDISLLDAQSAPLRV
AMAASDISLLDAQSAPLRVYVEELKPTPEGDL
AMAASDISLLDAQSAPLRVYVEELKPTPEGDLEIL
AQALIVTQTMK
AQSAPLRVYVEELKPTPEGDL
AQSAPLRVYVEELKPTPEGDLE
AQSAPLRVYVEELKPTPEGDLEI
AQSAPLRVYVEELKPTPEGDLEIL
ASDISLLDAQ
ASDISLLDAQSAPL
ASDISLLDAQSAPLR
ASDISLLDAQSAPLRV
ASDISLLDAQSAPLRVYVEELKPTPEGD
ASDISLLDAQSAPLRVYVEELKPTPEGDL
ASDISLLDAQSAPLRVYVEELKPTPEGDLEI
ASDISLLDAQSAPLRVYVEELKPTPEGDLEIL
ASGEPTSTPTIE

ASGEPTSTPTIEA
ASPEVIESPPEINT
DAFLGSFLYEYSR
DAIPENLPPLTAD
DALNENKVLV
DALNENKVLVLDTDY
DALNENKVLVLDTDYKK
DAQSAPLRV
DAQSAPLRVY
DAQSAPLRVYVE
DAQSAPLRVYVEE
DAQSAPLRVYVEELKPTPEGD
DAQSAPLRVYVEELKPTPEGDL
DAQSAPLRVYVEELKPTPEGDLE
DAQSAPLRVYVEELKPTPEGDLEI
DAQSAPLRVYVEELKPTPEGDLEIL
DAQSAPLRVYVEQLKPTPEGD
DAQSAPLRVYVEQLKPTPEGDL
DDEALEKFDK
DDEALEKFDKA
DDEALEKFDKAL
DDEALEKFDKALKALPMHIRL
DEALEKFDK
DEALEKFDKA
DEALEKFDKAL
DEALEKFDKALK
DEALEKFDKALKALPM
DEALEKFDKALKALPMHI
DEALEKFDKALKALPMHIRL
DEALEKFDKALKALPMHIRLSFNPTQL
DELQDKIHP
DELQDKIHPF
DELQDKIHPFA
DELQDKIHPFAQT
DELQDKIHPFAQTQS
DELQDKIHPFAQTQSL
DISLLDAQSAPLRV
DKALKALPMHI
DKIHPFAQT
DKIHPFAQTQS
DKIHPFAQTQSL
DKLKHLVDEPQN
DKLKHLVDEPQNL
DKTEIPTINT
DKVGINY
DKVGINYW

DLKGYGGVS
DLKGYGGVSL
DLKGYGGVSLPE
DLKGYGGVSLPEW
DLKGYGGVSLPEWV
DLSKEPSISRE
DLSKEPSISREDL
DSPEVIESPPEINT
DTDYKKYL
DTDYKKYLL
DTDYKKYLLF
DTHKSEIAHRFKDLGEEHFKG
DTHKSEIAHRFKDLGEEHFKGLVL
DVENLHLPLP
DVENLHLPLPL
EALEKFDK
EALEKFDKA
EALEKFDKAL
EALEKFDKALK
EALEKFDKALKALPM
EALEKFDKALKALPMHI
EALEKFDKALKALPMHIR
EALEKFDKALKALPMHIRL
EALEKFDKALKALPMHIRLSFNPTQL
EAMAPKHKEMPFPK
EAMAPKHKEMPFPKYPVE
EAMAPKHKEMPFPKYPVEPF
EAMAPKHKEMPFPKYPVEPFTESQS
EAQPTDASAQF
EASPEVIESPPEIN
EDSPEVIEGPPEINT
EDSPEVIESPPEINT
EELKPTPEGD
EELKPTPEGDL
EELKPTPEGDLE
EELKPTPEGDLEI
EELKPTPEGDLEIL
EKFDKAL
EKFDKALK
EKFDKALKA
EKFDKALKALP
EKFDKALKALPM
EKFDKALKALPMH
EKFDKALKALPMHI
EKFDKALKALPMHIR
EKFDKALKALPMHIRL

EKTKIPAVF
ELEELNVPGE
ELEELNVPGEIVE
ELGHKIM
ELKDLKG
ELKDLKGYGG
ELKDLKGYGGVS
ELKDLKGYGGVSL
ELKDLKGYGGVSLPEWV
ELKPTPEGD
ELKPTPEGDL
ELKPTPEGDLE
ELKPTPEGDLEI
ELKPTPEGDLEIL
ELQDKIHPF
ELQDKIHPFAQTQ
ELQDKIHPFAQTQSL
EMPFPKYPVE
EMPFPKYPVEPF
EMPFPKYPVEPFTESQ
EMPFPKYPVEPFTESQS
ENSAEPEQSL
ENSAEPEQSLA
EPVLGPVRGPFPIIV
ESPPEINT
EVDDEALEKFDKA
EVIESPPEINT
FAQTQSLVYPFPGP
FDKALKALPM
FDKALKALPMHI
FDKLKHLVDEPQNL
FEVVKT
FHTSGYDTQA
FKDLGEEH
FKDLGEEHFK
FKDLGEEHFKGLV
FKIDALNE
FKIDALNENKVLV
FMAIPPKKNQDKTEIPTINT
FPPQSVL
FQINNKIW
FQINNKIWCKDDQNPHSSNICNISCDKFLDDDLTDDIMCVKKILDKVGIN
FQSEEQQQTEDELQDKIHP
FVAPFPEV
FVAPFPEVFG
FVAPFPEVFGK

FVAPFPEVFGKE
FVAPFPEVFGKEK
FVAPFPEVFGKEKVNE
FVAPFPEVFGKEKVNEL
GEPTSTPTIE
GGVSLPEW
GGVSLPEWV
GLDIQK
GLDIQKV
GLDIQKVA
GLDIQKVAGT
GLDIQKVAGTW
GLDIQKVAGTWY
GLDIQKVAGTWYSLA
GLDIQKVAGTWYSLAM
GLDIQKVAGTWYSLAMA
GPIHNSLPQNIPPLTQT
GVSKVKEAM
GVSKVKEAMAP
GVSKVKEAMAPK
GVSKVKEAMAPKH
GVSKVKEAMAPKHK
GVSKVKEAMAPKHKEMPFPK
GVSKVKEAMAPKHKEMPFPKYPVE
GVSKVKEAMAPKHKEMPFPKYPVEPF
GVSKVKEAMAPKHKEMPFPKYPVEPFT
GVSKVKEAMAPKHKEMPFPKYPVEPFTES
GVSKVKEAMAPKHKEMPFPKYPVEPFTESQ
GVSKVKEAMAPKHKEMPFPKYPVEPFTESQS
GVSKVKEAMAPKHKEMPFPKYPVEPFTESQSL
GVSKVKEAMAPKHKEMPFPKYPVEPFTESQSLT
GVSKVKEAMAPKHKEMPFPKYPVQPFTESQS
GYGGVSLPEWV
HIQKEDVPSE
HIQKEDVPSER
HIQKEDVPSERYLG
HIRLSFNPT
HIRLSFNPTQ
HIRLSFNPTQL
HKEMPFPK
HKEMPFPKYPVE
HKEMPFPKYPVEP
HKEMPFPKYPVEPF
HKEMPFPKYPVEPFTES
HKEMPFPKYPVEPFTESQS
HLPLPLL

HLVDEPQN
HLVDEPQNL
HNSLPQNIPPLTQT
HPYFYAPEL
HQGLPQPFPEV
HQPHQPLPPT
HQPHQPLPPTVM
HVKLVNE
IAEKTKIPA
IAEKTKIPAV
IASGEPTSTPITE
IASGEPTSTPTIE
IASGEPTSTPTIEA
IASGEPTSTPTTE
IASGEPTSTPTTEA
ICNISCDKFLDDDLTDDIMCVKKILDKVGIN
ICNISCDKFLDDDLTDDIMCVKKILDKVGINY
IDALNENK
IDALNENKV
IDALNENKVL
IDALNENKVLV
IDALNENKVLVLDTDY
IDALNENKVLVLDTDYK
IDALNENKVLVLDTDYKK
IESPPEINT
IHPFAQTQS
IHPFAQTQSL
IIAEKTKIPA
IIAEKTKIPAV
IIAEKTKIPAVF
IIAEKTKIPAVFKID
IIAEKTKIPAVFKIDA
IIAEKTKIPAVFKIDALNENKVLV
IIAEKTKIPAVFKIDALNENKVLVLDTDYK
IIVTQTM
IIVTQTMKG
IIVTQTMKGL
IIVTQTMKGLDI
IIVTQTMKGLDIQ
IIVTQTMKGLDIQK
IIVTQTMKGLDIQKV
IIVTQTMKGLDIQKVA
IIVTQTMKGLDIQKVAGT
IIVTQTMKGLDIQKVAGTW
IIVTQTMKGLDIQKVAGTWYSL
IIVTQTMKGLDIQKVAGTWYSLAM

IIVTQTMKGLDIQKVAGTWYSLAMAA
IIVTQTMKGLDIQKVAGTWYSLAMAASD
IIVTQTMKGLDIQKVAGTWYSLAMAASDISLLD
IIVTQTMKGLDIQKVAGTWYSLAMAASDISLLDAQSAPLR
ILDKVGIN
ILDKVGINY
ILNKPEDET
ILNKPEDETH
ILNKPEDETHL
IMCVKKILDKVGIN
IMCVKKILDKVGINY
IPAVFKIDAL
IPAVFKIDALNENKVLVLDTDYK
IPPKKNQDKTEIPT
IPPKKNQDKTEIPTINT
IQKEDVPSER
IQKVAGTW
IQKVAGTWYSLA
IQKVAGTWYSLAM
IRNLQISNE
IRNLQISNEDL
ISKEQIVI
ISKEQIVIR
ISKEQIVIRSS
ISLLDAQSAPLR
ISLLDAQSAPLRV
ISLLDAQSAPLRVY
ISLLDAQSAPLRVYVE
ISLLDAQSAPLRVYVEELKPTPEGD
ISLLDAQSAPLRVYVEELKPTPEGDL
ISLLDAQSAPLRVYVEELKPTPEGDLE
ISLLDAQSAPLRVYVEELKPTPEGDLEIL
IVQNNDSTEY
IVQNNDSTEYG
IVQNNDSTEYGL
IVRYTRKVPQVSTPT
IVRYTRKVPQVSTPTL
IVTQTMKG
IVTQTMKGL
IVTQTMKGLD
IVTQTMKGLDIQ
IVTQTMKGLDIQK
IVTQTMKGLDIQKVAGT
IVTQTMKGLDIQKVAGTW
IVTQTMKGLDIQKVAGTWYSL
IVTQTMKGLDIQKVAGTWYSLA

KADEKKFWG
KADEKKFWGKY
KALKALPM
KALKALPMHI
KALKALPMHIRL
KALKALPMHIRLSFNPTQL
KALPMHI
KALPMHIRL
KDLGEEHF
KDLKGYGGVSL
KFDKALKALPM
KFDKALKALPMHI
KFDKALKALPMHIRL
KFLDDDL
KFLDDDLTDD
KGLDIQK
KGLDIQKVAGT
KGLDIQKVAGTW
KIDALNE
KIDALNEN
KIDALNENK
KIDALNENKV
KIDALNENKVL
KIDALNENKVLV
KIDALNENKVLVL
KIDALNENKVLVLD
KIDALNENKVLVLDT
KIDALNENKVLVLDTD
KIDALNENKVLVLDTDY
KIDALNENKVLVLDTDYK
KIDALNENKVLVLDTDYKK
KIDALNENKVLVLDTDYKKY
KIDALNENKVLVLDTDYKKYL
KIDALNENKVLVLDTDYKKYLL
KIDALNENKVLVLDTDYKKYLLF
KIDALNENKVLVLDTDYKKYLLFCMENSAEPEQSLVCQ
KIDALNENKVLVLDTDYKKYLLFCMENSAEPEQSLVCQCL
KIEKFQSEEQQQTEDEL
KIEKFQSEEQQQTEDELQDKIHP
KIEKFQSEEQQQTEDELQDKIHPF
KIEKFQSEEQQQTEDELQDKIHPFAQTQSL
KIHPFAQT
KIHPFAQTQ
KIHPFAQTQS
KIHPFAQTQSL
KIIAEKTKIPA

KIIAEKTKIPAV
KILDKVGIN
KILDKVGINY
KNQDKTEIPT
KNQDKTEIPTI
KNQDKTEIPTINT
KPTPEGDLE
KPTPEGDLEI
KPTPEGDLEIL
KPTPEGDLEILL
KTEIPTINT
KTKIPAV
KVPQVSTPT
LDAQSAPLR
LDAQSAPLRV
LDAQSAPLRVY
LDAQSAPLRVYVE
LDAQSAPLRVYVEE
LDAQSAPLRVYVEELKPTPEGD
LDAQSAPLRVYVEELKPTPEGDL
LDAQSAPLRVYVEELKPTPEGDLE
LDAQSAPLRVYVEELKPTPEGDLEI
LDAQSAPLRVYVEELKPTPEGDLEIL
LDAQSAPLRVYVEQLKPTPEGDLEIL
LDIQKVAGT
LDIQKVAGTW
LDKVGIN
LEDSPEVIESPPEINT
LEKFDK
LEKFDKA
LEKFDKAL
LEKFDKALK
LEKFDKALKALPM
LEKFDKALKALPMH
LEKFDKALKALPMHI
LEKFDKALKALPMHIR
LEKFDKALKALPMHIRL
LEKFDKALKALPMHIRLSFNPTQL
LIVTQTMK
LIVTQTMKGL
LIVTQTMKGLD
LIVTQTMKGLDI
LIVTQTMKGLDIQK
LIVTQTMKGLDIQKVAG
LIVTQTMKGLDIQKVAGTWY
LIVTQTMKGLDIQKVAGTWYS

LIVTQTMKGLDIQKVAGTWYSL
LIVTQTMKGLDIQKVAGTWYSLA
LIVTQTMKGLDIQKVAGTWYSLAM
LIVTQTMKGLDIQKVAGTWYSLAMA
LIVTQTMKGLDIQKVAGTWYSLAMAASD
LIVTQTMKGLDIQKVAGTWYSLAMAASDISL
LIVTQTMKGLDIQKVAGTWYSLAMAASDISLL
LKALPMHIRL
LKDLKGY
LKDLKGYGGVS
LKDLKGYGGVSL
LKGYGGVSLPEW
LKPTPEGDL
LKPTPEGDLE
LKPTPEGDLEIL
LLDAQSAPLRV
LNENKVLV
LNENKVLVLDTDYKKYL
LNENKVLVLDTDYKKYLL
LNKPEDETHL
LPKLKPD
LPMHIRL
LPQNIPPLTQT
LQDKIHP
LQDKIHPF
LQDKIHPFAQ
LQDKIHPFAQT
LQDKIHPFAQTQ
LQDKIHPFAQTQS
LQDKIHPFAQTQSL
LQKWENGECAQKKIIAEKTKIPAV
LRLAKEYEAT
LRLKKYKVPQL
LSFNPTQL
LSFNPTQLEEQ
LSFNPTQLEEQC
LSFNPTQLEEQCH
LSFNPTQLEEQCHI
LSQKFPKAEF
LTDVENLHLPLPL
LTDVENLHLPLPLL
LVLDTDYK
LVLDTDYKK
LVLDTDYKKYL
LVLDTDYKKYLL
LVRTPEVDDEALEKFDKALKALPMHI

LVYPFPGPIHN
LVYPFPGPIHNSLPQNIPPLTQT
LVYPFPGPIPN
LVYPFPGPIPNS
LVYPFPGPIPNSL
LVYPFPGPIPNSLPQNIPPLTQT
LYQEPVLGPVRGPFPIIV
LYYANKYNGV
MAASDISLL
MAASDISLLDAQ
MAASDISLLDAQSAPL
MAASDISLLDAQSAPLRV
MAASDISLLDAQSAPLRVY
MAASDISLLDAQSAPLRVYVE
MAASDISLLDAQSAPLRVYVEELKPTPEGDL
MAASDISLLDAQSAPLRVYVEELKPTPEGDLE
MAASDISLLDAQSAPLRVYVEELKPTPEGDLEIL
MAIPPK
MAIPPKK
MAIPPKKNQD
MAIPPKKNQDK
MAIPPKKNQDKTEIPT
MAIPPKKNQDKTEIPTI
MAIPPKKNQDKTEIPTIN
MAIPPKKNQDKTEIPTINT
MELGHKIM
MELGHKIMRNLEN
MELGHKIMRNLENT
MELGHKIMRNLENTVKE
MELGHKIMRNLENTVKETIK
MELGHKIMRNLENTVKETIKY
MELGHKIMRNLENTVKETIKYLKS
MELGHKIMRNLENTVKETIKYLKSL
MENSAEPEQSLA
MGVSKVKEAMAPK
MHQPHQPLPPT
MHQPHQPLPPTVM
NENKVLV
NENKVLVL
NENKVLVLD
NENKVLVLDTDY
NENKVLVLDTDYK
NENKVLVLDTDYKK
NENKVLVLDTDYKKY
NENKVLVLDTDYKKYL
NENKVLVLDTDYKKYLL

NIPPLTQT
NKPEDETHL
NKVLVLDTDY
NKVLVLDTDYK
NKVLVLDTDYKK
NKVLVLDTDYKKY
NKVLVLDTDYKKYL
NKVLVLDTDYKKYLL
NLENTVK
NLENTVKET
NLENTVKETIK
NLENTVKETIKYLKS
NLENTVKETIKYLKSL
NPKLPLSIL
NQDKTEIPTINT
NSAEPEQSL
NSAEPEQSLA
PEVIESPPEINT
PMHIRL
PPKKNQDKTEIPTI
PPKKNQDKTEIPTINT
PPKKNQDKTEIPTINTI
PPKKNQDKTEIPTINTIA
PPLTQTPVVVPPFLQPEVMGVSKVKEAMAPKHKEMPF
PPLTQTPVVVPPFLQPEVMGVSKVKEAMAPKHKEMPFP
PQNIPPLTQT
PQNIPPLTQTPVVVPPFIQPEV
PTPEGDLEIL
PVLGPVRGPFPIIV
PVVVPPF
PVVVPPFIQPEVMG
PVVVPPFIQPEVMGV
PVVVPPFIQPEVMGVSK
PVVVPPFIQPEVMGVSKVK
PVVVPPFIQPEVMGVSKVKEAMAPKHK
PVVVPPFL
PVVVPPFLQ
PVVVPPFLQP
PVVVPPFLQPE
PVVVPPFLQPEV
PVVVPPFLQPEVM
PVVVPPFLQPEVMG
PVVVPPFLQPEVMGV
PVVVPPFLQPEVMGVS
PVVVPPFLQPEVMGVSKV
PVVVPPFLQPEVMGVSKVKE

PVVVPPFLQPEVMGVSKVKEAMAPK
PVVVPPFLQPEVMGVSKVKEAMAPKH
QDKIHPF
QDKIHPFA
QDKIHPFAQT
QDKIHPFAQTQ
QDKIHPFAQTQS
QDKIHPFAQTQSL
QDKTEIPTINT
QEPVLGPVRGPFPIIV
QEPVLGPVRGPFPILV
QINNKIWCKDDQNPHSSNICNISCDKFLDDDLTDDIMCVKKILDKVGIN
QINNKIWCKDDQNPHSSNICNISCDKFLDDDLTDDIMCVKKILDKVGINY
QLKPTPEGDL
QPQSQNPKLPL
QPQSQNPKLPLS
QPQSQNPKLPLSIL
QSAPLRV
QSLVYPFPGPIHNSLPQNIPPLTQT
QSLVYPFPGPIPN
QSLVYPFPGPIPNSLPQNIPPLTQT
QSWMHQPHQPLPPT
QSWMHQPHQPLPPTV
QSWMHQPHQPLPPTVM
QTMKGLDIQKVAGT
QTMKGLDIQKVAGTW
RELEELNVPG
RELEELNVPGE
RELEELNVPGEI
RELEELNVPGEIVE
RELKDLKG
RELKDLKGY
RELKDLKGYGG
RELKDLKGYGGVS
RELKDLKGYGGVSL
RELKDLKGYGGVSLPE
RELKDLKGYGGVSLPEW
RELKDLKGYGGVSLPEWVCTTFHTSGYDTQAIVQNN
RHPEYAV
RHPYFYAPEL
RLSFNPT
RLSFNPTQ
RLSFNPTQL
RPKHPIKHQGLPQEV
RPKHPIKHQGLPQEVL
RPKHPIKHQGLPQEVLNE

RPKHPIKHQGLPQPFPEV
RTPEVDDEA
RTPEVDDEALEKFDK
RTPEVDDEALEKFDKA
RVYVEELKPTPEGD
RVYVEELKPTPEGDL
RVYVEELKPTPEGDLE
RVYVEELKPTPEGDLEI
RVYVEELKPTPEGDLEIL
RVYVEELKPTPEGDLEILL
RVYVEELKPTPEGDLEILLQ
RVYVEELKPTPEGDLEILLQK
RVYVEELKPTPEGDLEILLQKWEND
SAEPEQSL
SAEPEQSLA
SAPLRV
SAPLRVYVEELKPTPEGDL
SAPLRVYVEELKPTPEGDLEIL
SDIPNPIGSENSEK
SDIPNPIGSENSEKT
SDIPNPIGSENSEKTTM
SDIPNPIGSENSEKTTMPL
SDISLLDAQ
SDISLLDAQSAPL
SDISLLDAQSAPLRV
SDISLLDAQSAPLRVY
SDISLLDAQSAPLRVYVEELKPTPEGD
SDISLLDAQSAPLRVYVEELKPTPEGDL
SDISLLDAQSAPLRVYVEELKPTPEGDLE
SDISLLDAQSAPLRVYVEELKPTPEGDLEIL
SEEQQQTEDELQDK
SFNPTQL
SFNPTQLEEQ
SFNPTQLEEQCHI
SGEPTSTPTIE
SGEPTSTPTIEA
SKEPSISREDL
SKVKEAMAPK
SLAMAASDISL
SLAMAASDISLL
SLAMAASDISLLD
SLAMAASDISLLDAQ
SLAMAASDISLLDAQSAPL
SLAMAASDISLLDAQSAPLR
SLAMAASDISLLDAQSAPLRV
SLAMAASDISLLDAQSAPLRVY

SLAMAASDISLLDAQSAPLRVYVEELKPTPEGD
SLLDAQSAPLR
SLLDAQSAPLRV
SLPQNIPPLTQT
SLSQSKVLPVPEKAVPYPQ
SLSQSKVLPVPEKAVPYPQRD
SLSQSKVLPVPEKAVPYPQRDMPIQ
SLSQSKVLPVPEKAVPYPQRDMPIQA
SLSQSKVLPVPQ
SLSQSKVLPVPQK
SLSQSKVLPVPQKAVPYPQ
SLSQSKVLPVPQKAVPYPQRD
SLSQSKVLPVPQKAVPYPQRDMPIQ
SLSQSKVLPVPQKAVPYPQRDMPIQA
SLVYPFPGPIHNSLPQNIPPLTQT
SLVYPFPGPIPNSLPQNIPPLTQT
SPEVIEGPPEINT
SPEVIESPPEINT
SPPEINT
SQKFPKAEF
SRQPQSQNPKLPLSIL
SSRQPQSQNPKLPL
SSRQPQSQNPKLPLS
SSRQPQSQNPKLPLSI
SSRQPQSQNPKLPLSIL
TDVENLHLPLP
TDVENLHLPLPL
TDVENLHLPLPLL
TDVENLHLPLPLLQ
TDVENLHLPPL
TDYKKYL
TDYKKYLL
TEDELQDKIHPF
TEIPTIN
TEIPTINT
TFHTSGYDTQA
TIASGEPTSTPTIE
TIASGEPTSTPTTE
TIKYLKSL
TIKYLKSLFSHA
TKIPAV
TKIPAVFKID
TLEDSPEVIESPPEINT
TLTDVENLHLPLPL
TMKGLDIQK
TMKGLDIQKVAGT

TMKGLDIQKVAGTW
TPEVDDEAL
TPEVDDEALEK
TPEVDDEALEKF
TPEVDDEALEKFD
TPEVDDEALEKFDKA
TPEVDDEALEKFDKAL
TPEVDDEALEKFDKALK
TPEVDDEALEKFDKALKALPM
TPEVDDEALEKFDKALKALPMHI
TQTMKGLDIQKVAGT
TQTMKGLDIQKVAGTW
TRKVPQVSTPT
TVKETIKYL
TVKETIKYLKS
TVKETIKYLKSL
TVKETIKYLKSLFSHA
TVMENFVAFVDK
VATLEDSPEVIESPPEINT
VDDEALEKFDK
VDDEALEKFDKA
VEELKPTPE
VEELKPTPEG
VEELKPTPEGD
VEELKPTPEGDL
VEELKPTPEGDLE
VEELKPTPEGDLEI
VEELKPTPEGDLEIL
VEELKPTPEGDLEILL
VEELKPTPEGDLEILLQ
VEELKPTPEGDLEILLQK
VEELKPTPEGDLEILLQKWEND
VEELKPTPEGDLEILLQKWENDECAQKK
VEKDAIPENLPPLTADFAEDKD
VELLKHKPK
VELLKHKPKATEEQ
VELLKHKPKATEEQL
VELLKHKPKATEEQLKT
VELLKHKPKATEEQLKTVM
VENLHLPLPL
VENLHLPLPLL
VKKILDKVGIN
VLDTDYK
VLDTDYKK
VLDTDYKKYL
VLDTDYKKYLL

VLDTDYKKYLLF
VLPVPQK
VLPVPQKAVPYPQRDMPIQA
VLVLDTDYK
VLVLDTDYKK
VLVLDTDYKKY
VLVLDTDYKKYL
VLVLDTDYKKYLL
VPPFLQPEV
VPPFLQPEVM
VQVTSTAV
VRTPEVDD
VRTPEVDDE
VRTPEVDDEA
VRTPEVDDEAL
VRTPEVDDEALE
VRTPEVDDEALEK
VRTPEVDDEALEKF
VRTPEVDDEALEKFD
VRTPEVDDEALEKFDK
VRTPEVDDEALEKFDKA
VRTPEVDDEALEKFDKAL
VRTPEVDDEALEKFDKALK
VRTPEVDDEALEKFDKALKA
VRTPEVDDEALEKFDKALKALP
VRTPEVDDEALEKFDKALKALPM
VRTPEVDDEALEKFDKALKALPMH
VRTPEVDDEALEKFDKALKALPMHI
VRTPEVDDEALEKFDKALKALPMHIR
VRTPEVDDEALEKFDKALKALPMHIRL
VRTPEVDDEALEKFDKALKALPMHIRLS
VRTPEVDDEALEKFDKALKALPMHIRLSFNPT
VRTPEVDDEALEKFDKALKALPMHIRLSFNPTQ
VRTPEVDDEALEKFDKALKALPMHIRLSFNPTQL
VRTPEVDDEALEKFDKALKALPMHIRLSFNPTQLEEQCHI
VSKVKEAMAPK
VSKVKEAMAPKH
VSKVKEAMAPKHK
VTQTMKGLD
VTQTMKGLDIQKVAGT
VTQTMKGLDIQKVAGTW
VTQTMKGLDIQKVAGTWYSL
VVVPPFIQPEV
VYPFPGPIH
VYPFPGPIHN
VYPFPGPIHNS

VYPFPGPIHNSL
VYPFPGPIHNSLPQ
VYPFPGPIHNSLPQNIPPLT
VYPFPGPIHNSLPQNIPPLTQT
VYPFPGPIHNSLPQNIPPLTQTPVVVPPF
VYPFPGPIP
VYPFPGPIPN
VYPFPGPIPNS
VYPFPGPIPNSL
VYPFPGPIPNSLPQ
VYPFPGPIPNSLPQNIPPLT
VYPFPGPIPNSLPQNIPPLTQ
VYPFPGPIPNSLPQNIPPLTQT
VYPFPGPIPNSLPQNIPPLTQTPV
VYVEELKPTPEGDL
VYVEELKPTPEGDLE
VYVEELKPTPEGDLEI
VYVEELKPTPEGDLEIL
VYVEELKPTPEGDLEILL
VYVEELKPTPEGDLEILLQ
VYVEELKPTPEGDLEILLQK
VYVEELKPTPEGDLEILLQKWEND
WLAHKALCSEKLDQ
WMHQPHQPLPPTV
WMHQPHQPLPPTVM
WSVARLSQKFPKAEF
YPFPGPIHN
YPFPGPIHNSLPQ
YPFPGPIHNSLPQNIPPLTQT
YPFPGPIPN
YPFPGPIPNS
YPFPGPIPNSLPQNIPPLTQT
YQEPVLGPVRGPFPI
YQEPVLGPVRGPFPILV
YSLAMAASDI
YSLAMAASDIS
YSLAMAASDISL
YSLAMAASDISLL
YSLAMAASDISLLDAQ
YSLAMAASDISLLDAQSAPL
YSLAMAASDISLLDAQSAPLR
YSLAMAASDISLLDAQSAPLRV
YSLAMAASDISLLDAQSAPLRVYVEELKPTPEGDL
YSLAMAASDISLLDAQSAPLRVYVEELKPTPEGDLEIL
YVEELKPTPE
YVEELKPTPEG

YVEELKPTPEGD
YVEELKPTPEGDL
YVEELKPTPEGDLE
YVEELKPTPEGDLEI
YVEELKPTPEGDLEIL
YVEELKPTPEGDLEILL
YVEELKPTPEGDLEILLQ
YVEELKPTPEGDLEILLQK
YVEQLKPTPEGDL
YVPKAFDEK
YVPKAFDEKL
YYANKYNGV
YYANKYNGVF

WPH.A.60min
AASDISLLDAQ
AASDISLLDAQSAPL
AASDISLLDAQSAPLRV
AASDISLLDAQSAPLRVY
AASDISLLDAQSAPLRVYVE
AASDISLLDAQSAPLRVYVEE
AASDISLLDAQSAPLRVYVEELKPTPEGD
AASDISLLDAQSAPLRVYVEELKPTPEGDL
AASDISLLDAQSAPLRVYVEELKPTPEGDLE
AASDISLLDAQSAPLRVYVEELKPTPEGDLEI
AASDISLLDAQSAPLRVYVEELKPTPEGDLEIL
AASDISLLDAQSAPLRVYVEQLKPTPEGDL
AASDISLLDAQSAPLRVYVEQLKPTPEGDLEIL
ADEKKFWG
AEKTKIPAV
AIPENLPPLTAD
AIPPKKNQD
AIPPKKNQDKTEIPTINT
AIVQNNDSTEYG
ALEKFDK
ALEKFDKA
ALEKFDKAL
ALEKFDKALK
ALEKFDKALKA
ALEKFDKALKALPM
ALEKFDKALKALPMH
ALEKFDKALKALPMHI
ALEKFDKALKALPMHIR

ALEKFDKALKALPMHIRL
ALEKFDKALKALPMHIRLSFNPTQL
ALKALPMHI
ALKALPMHIRL
ALNENKVLVLDTDY
ALNENKVLVLDTDYK
ALNENKVLVLDTDYKK
ALNENKVLVLDTDYKKYL
ALNENKVLVLDTDYKKYLL
ALPMHI
ALPMHIRL
ALPMHIRLSFNPT
ALPMHIRLSFNPTQL
AMAASDISLLDAQSAPL
AMAASDISLLDAQSAPLRV
AMAASDISLLDAQSAPLRVYVEELKPTPEGDL
AMAASDISLLDAQSAPLRVYVEELKPTPEGDLEIL
AQALIVTQTMK
AQSAPLRVYVEELKPTPEGDL
AQSAPLRVYVEELKPTPEGDLE
AQSAPLRVYVEELKPTPEGDLEI
AQSAPLRVYVEELKPTPEGDLEIL
ASDISLLDAQ
ASDISLLDAQSAPL
ASDISLLDAQSAPLR
ASDISLLDAQSAPLRV
ASDISLLDAQSAPLRVYVEELKPTPEGD
ASDISLLDAQSAPLRVYVEELKPTPEGDL
ASDISLLDAQSAPLRVYVEELKPTPEGDLEI
ASDISLLDAQSAPLRVYVEELKPTPEGDLEIL
ASGEPTSTPTIE
ASGEPTSTPTIEA
ASPEVIESPPEINT
DAIPENLPPLTAD
DALNENKVLV
DALNENKVLVLDTDY
DAQSAPLRV
DAQSAPLRVY
DAQSAPLRVYVE
DAQSAPLRVYVEE
DAQSAPLRVYVEELKPTPEGD
DAQSAPLRVYVEELKPTPEGDL
DAQSAPLRVYVEELKPTPEGDLE
DAQSAPLRVYVEELKPTPEGDLEI
DAQSAPLRVYVEELKPTPEGDLEIL
DAQSAPLRVYVEQLKPTPEGD

DAQSAPLRVYVEQLKPTPEGDL
DDEALEKFDK
DDEALEKFDKA
DDEALEKFDKAL
DDEALEKFDKALKALPMHIRL
DEALEKFDK
DEALEKFDKA
DEALEKFDKAL
DEALEKFDKALK
DEALEKFDKALKALPM
DEALEKFDKALKALPMHI
DEALEKFDKALKALPMHIRL
DEALEKFDKALKALPMHIRLSFNPTQL
DELQDKIHP
DELQDKIHPF
DELQDKIHPFA
DELQDKIHPFAQT
DELQDKIHPFAQTQS
DELQDKIHPFAQTQSL
DISLLDAQSAPLRV
DKALKALPMHI
DKIHPFAQT
DKIHPFAQTQS
DKIHPFAQTQSL
DKLKHLVDEPQN
DKLKHLVDEPQNL
DKTEIPTINT
DKVGINY
DKVGINYW
DLKGYGGVS
DLKGYGGVSL
DLKGYGGVSLPE
DLKGYGGVSLPEW
DLKGYGGVSLPEWV
DLSKEPSISRE
DLSKEPSISREDL
DSPEVIEGPPEINT
DSPEVIESPPEINT
DTDYKKYL
DTDYKKYLL
DTHKSEIAHRFKDLGEEHFKG
DVENLHLPLP
DVENLHLPLPL
EALEKFDK
EALEKFDKA
EALEKFDKAL

EALEKFDKALK
EALEKFDKALKALPM
EALEKFDKALKALPMHI
EALEKFDKALKALPMHIR
EALEKFDKALKALPMHIRL
EALEKFDKALKALPMHIRLSFNPTQL
EAMAPKHKEMPFPK
EAMAPKHKEMPFPKYPVE
EAMAPKHKEMPFPKYPVEPF
EAMAPKHKEMPFPKYPVEPFTESQS
EAQPTDASAQF
EASPEVIESPPEIN
EDSPEVIEGPPEINT
EDSPEVIESPPEINT
EELKPTPEGD
EELKPTPEGDL
EELKPTPEGDLE
EELKPTPEGDLEI
EELKPTPEGDLEIL
EKFDKAL
EKFDKALK
EKFDKALKA
EKFDKALKALP
EKFDKALKALPM
EKFDKALKALPMH
EKFDKALKALPMHI
EKFDKALKALPMHIR
EKFDKALKALPMHIRL
ELEELNVPGE
ELEELNVPGEIVE
ELGHKIM
ELKDLKG
ELKDLKGYGG
ELKDLKGYGGVS
ELKDLKGYGGVSL
ELKDLKGYGGVSLPEWV
ELKPTPEGD
ELKPTPEGDL
ELKPTPEGDLE
ELKPTPEGDLEI
ELKPTPEGDLEIL
ELQDKIHPF
ELQDKIHPFAQTQ
ELQDKIHPFAQTQSL
EMPFPKYPVE
EMPFPKYPVEPF

EMPFPKYPVEPFTESQ
EMPFPKYPVEPFTESQS
ENSAEPEQSLA
EPVLGPVRGPFPIIV
ESPPEINT
EVDDEALEKFDK
EVDDEALEKFDKA
EVIESPPEINT
FAQTQSLVYPFPGP
FDKALKALPM
FDKALKALPMHI
FDKLKHLVDEPQNL
FEVVKT
FHTSGYDTQA
FKDLGEEH
FKDLGEEHFK
FKDLGEEHFKGLV
FKIDALNE
FKIDALNENKVLV
FMAIPPKKNQDKTEIPTINT
FPGPIPNSLPQNIPPLTQT
FPPQSVL
FQINNKIW
FQINNKIWCKDDQNPHSSNICNISCDKFLDDDLTDDIMCVKKILDKVGIN
FQSEEQQQTEDELQDKIHP
FVAPFPEV
FVAPFPEVFG
FVAPFPEVFGK
FVAPFPEVFGKE
FVAPFPEVFGKEK
FVAPFPEVFGKEKVNE
FVAPFPEVFGKEKVNEL
GEPTSTPTIE
GGVSLPEW
GGVSLPEWV
GLDIQK
GLDIQKV
GLDIQKVA
GLDIQKVAGT
GLDIQKVAGTW
GLDIQKVAGTWY
GLDIQKVAGTWYSLA
GLDIQKVAGTWYSLAM
GLDIQKVAGTWYSLAMA
GPIHNSLPQNIPPLTQT
GVSKVKEAM

GVSKVKEAMAP
GVSKVKEAMAPK
GVSKVKEAMAPKH
GVSKVKEAMAPKHK
GVSKVKEAMAPKHKEMPFPK
GVSKVKEAMAPKHKEMPFPKYPVE
GVSKVKEAMAPKHKEMPFPKYPVEPF
GVSKVKEAMAPKHKEMPFPKYPVEPFT
GVSKVKEAMAPKHKEMPFPKYPVEPFTES
GVSKVKEAMAPKHKEMPFPKYPVEPFTESQ
GVSKVKEAMAPKHKEMPFPKYPVEPFTESQS
GVSKVKEAMAPKHKEMPFPKYPVEPFTESQSL
GVSKVKEAMAPKHKEMPFPKYPVEPFTESQSLT
GVSKVKEAMAPKHKEMPFPKYPVQPFTESQS
GYGGVSLPEWV
HIQKEDVPSE
HIQKEDVPSER
HIQKEDVPSERYLG
HIRLSFNPT
HIRLSFNPTQ
HIRLSFNPTQL
HKEMPFPK
HKEMPFPKYPVE
HKEMPFPKYPVEP
HKEMPFPKYPVEPF
HKEMPFPKYPVEPFTES
HKEMPFPKYPVEPFTESQS
HLPLPLL
HLVDEPQN
HLVDEPQNL
HNSLPQNIPPLTQT
HPYFYAPEL
HQGLPQPFPEV
HQPHQPLPPT
HQPHQPLPPTVM
HVKLVNE
IAEKTKIPA
IAEKTKIPAV
IASGEPTSTPITE
IASGEPTSTPTIE
IASGEPTSTPTIEA
IASGEPTSTPTTE
IASGEPTSTPTTEA
ICNISCDKFLDDDLTDDIMCVKKILDKVGIN
IDALNENK
IDALNENKV

IDALNENKVL
IDALNENKVLV
IDALNENKVLVLDTDY
IDALNENKVLVLDTDYK
IDALNENKVLVLDTDYKK
IESPPEINT
IHPFAQTQS
IHPFAQTQSL
IIAEKTKIPA
IIAEKTKIPAV
IIAEKTKIPAVF
IIAEKTKIPAVFKID
IIAEKTKIPAVFKIDA
IIAEKTKIPAVFKIDALNENKVLV
IIAEKTKIPAVFKIDALNENKVLVLDTDYK
IIVTQTM
IIVTQTMKG
IIVTQTMKGL
IIVTQTMKGLDI
IIVTQTMKGLDIQ
IIVTQTMKGLDIQK
IIVTQTMKGLDIQKV
IIVTQTMKGLDIQKVA
IIVTQTMKGLDIQKVAGT
IIVTQTMKGLDIQKVAGTW
IIVTQTMKGLDIQKVAGTWYSL
IIVTQTMKGLDIQKVAGTWYSLAM
IIVTQTMKGLDIQKVAGTWYSLAMAA
IIVTQTMKGLDIQKVAGTWYSLAMAASD
IIVTQTMKGLDIQKVAGTWYSLAMAASDISLLD
IIVTQTMKGLDIQKVAGTWYSLAMAASDISLLDAQSAPLR
ILDKVGIN
ILDKVGINY
ILNKPEDET
ILNKPEDETH
ILNKPEDETHL
IMCVKKILDKVGIN
IMCVKKILDKVGINY
IPAVFKIDALNENKVLVLDTDYK
IPPKKNQDKTEIPT
IPPKKNQDKTEIPTINT
IQKEDVPSER
IQKVAGTW
IQKVAGTWYSL
IQKVAGTWYSLA
IQKVAGTWYSLAM

IRNLQISNE
IRNLQISNEDL
ISKEQIVI
ISKEQIVIRSS
ISLLDAQSAPLR
ISLLDAQSAPLRV
ISLLDAQSAPLRVY
ISLLDAQSAPLRVYVE
ISLLDAQSAPLRVYVEELKPTPEGD
ISLLDAQSAPLRVYVEELKPTPEGDL
ISLLDAQSAPLRVYVEELKPTPEGDLE
ISLLDAQSAPLRVYVEELKPTPEGDLEIL
IVQNNDSTEY
IVQNNDSTEYG
IVQNNDSTEYGL
IVRYTRKVPQVSTPT
IVTQTMKG
IVTQTMKGL
IVTQTMKGLD
IVTQTMKGLDIQ
IVTQTMKGLDIQK
IVTQTMKGLDIQKVA
IVTQTMKGLDIQKVAGT
IVTQTMKGLDIQKVAGTW
IVTQTMKGLDIQKVAGTWYSL
IVTQTMKGLDIQKVAGTWYSLA
KADEKKFWG
KALKALPM
KALKALPMHI
KALKALPMHIRL
KALKALPMHIRLSFNPTQL
KALPMHI
KALPMHIRL
KDLKGYGGVSL
KFDKALKALPM
KFDKALKALPMHI
KFDKALKALPMHIRL
KFLDDDL
KFLDDDLTDD
KGLDIQK
KGLDIQKVAGT
KGLDIQKVAGTW
KIDALNE
KIDALNEN
KIDALNENK
KIDALNENKV

KIDALNENKVL
KIDALNENKVLV
KIDALNENKVLVL
KIDALNENKVLVLD
KIDALNENKVLVLDT
KIDALNENKVLVLDTD
KIDALNENKVLVLDTDY
KIDALNENKVLVLDTDYK
KIDALNENKVLVLDTDYKK
KIDALNENKVLVLDTDYKKY
KIDALNENKVLVLDTDYKKYL
KIDALNENKVLVLDTDYKKYLL
KIDALNENKVLVLDTDYKKYLLF
KIDALNENKVLVLDTDYKKYLLFCMENSAEPEQSLVCQ
KIDALNENKVLVLDTDYKKYLLFCMENSAEPEQSLVCQCL
KIEKFQSEEQQQTEDEL
KIEKFQSEEQQQTEDELQDKIHP
KIEKFQSEEQQQTEDELQDKIHPF
KIEKFQSEEQQQTEDELQDKIHPFAQTQSL
KIHPFAQT
KIHPFAQTQ
KIHPFAQTQS
KIHPFAQTQSL
KIIAEKTKIPA
KIIAEKTKIPAV
KILDKVGIN
KILDKVGINY
KNQDKTEIPT
KNQDKTEIPTINT
KPTPEGDLE
KPTPEGDLEI
KPTPEGDLEIL
KPTPEGDLEILL
KTEIPTINT
KTKIPAV
KVPQVSTPT
LDAQSAPLR
LDAQSAPLRV
LDAQSAPLRVY
LDAQSAPLRVYVE
LDAQSAPLRVYVEE
LDAQSAPLRVYVEELKPTPEGD
LDAQSAPLRVYVEELKPTPEGDL
LDAQSAPLRVYVEELKPTPEGDLE
LDAQSAPLRVYVEELKPTPEGDLEI
LDAQSAPLRVYVEELKPTPEGDLEIL

LDAQSAPLRVYVEQLKPTPEGDLEIL
LDIQKVAGT
LDIQKVAGTW
LDKVGIN
LEDSPEVIESPPEINT
LEKFDK
LEKFDKA
LEKFDKAL
LEKFDKALK
LEKFDKALKALPM
LEKFDKALKALPMH
LEKFDKALKALPMHI
LEKFDKALKALPMHIR
LEKFDKALKALPMHIRL
LEKFDKALKALPMHIRLSFNPTQL
LIVTQTMK
LIVTQTMKGL
LIVTQTMKGLD
LIVTQTMKGLDI
LIVTQTMKGLDIQK
LIVTQTMKGLDIQKVAG
LIVTQTMKGLDIQKVAGTWY
LIVTQTMKGLDIQKVAGTWYS
LIVTQTMKGLDIQKVAGTWYSL
LIVTQTMKGLDIQKVAGTWYSLA
LIVTQTMKGLDIQKVAGTWYSLAM
LIVTQTMKGLDIQKVAGTWYSLAMA
LIVTQTMKGLDIQKVAGTWYSLAMAASD
LIVTQTMKGLDIQKVAGTWYSLAMAASDISL
LIVTQTMKGLDIQKVAGTWYSLAMAASDISLL
LKALPMHIRL
LKDLKGYGGVS
LKDLKGYGGVSL
LKGYGGVSLPEW
LKPTPEGDL
LKPTPEGDLE
LKPTPEGDLEIL
LLDAQSAPLRV
LNENKVLV
LNENKVLVLDTDYKKYL
LNKPEDETHL
LPKLKPD
LPMHIRL
LPQNIPPLTQT
LQDKIHP
LQDKIHPF

LQDKIHPFAQ
LQDKIHPFAQT
LQDKIHPFAQTQ
LQDKIHPFAQTQS
LQDKIHPFAQTQSL
LQKWENGECAQKKIIAEKTKIPAV
LRLAKEYEAT
LSFNPTQL
LSFNPTQLEEQ
LSFNPTQLEEQC
LSFNPTQLEEQCH
LSFNPTQLEEQCHI
LSQKFPKAEF
LTDVENLHLPLPL
LTDVENLHLPLPLL
LVLDTDYK
LVLDTDYKK
LVLDTDYKKYL
LVLDTDYKKYLL
LVNELTEFAK
LVRTPEVDDEALEKFDKALKALPMHI
LVYPFPGPIHN
LVYPFPGPIHNSLPQNIPPLTQT
LVYPFPGPIPN
LVYPFPGPIPNS
LVYPFPGPIPNSL
LVYPFPGPIPNSLPQNIPPLTQT
LYQEPVLGPVRGPFPIIV
LYYANKYNGV
MAASDISLL
MAASDISLLDAQ
MAASDISLLDAQSAPL
MAASDISLLDAQSAPLRV
MAASDISLLDAQSAPLRVY
MAASDISLLDAQSAPLRVYVE
MAASDISLLDAQSAPLRVYVEELKPTPEGDL
MAASDISLLDAQSAPLRVYVEELKPTPEGDLE
MAASDISLLDAQSAPLRVYVEELKPTPEGDLEIL
MAIPPK
MAIPPKK
MAIPPKKNQD
MAIPPKKNQDK
MAIPPKKNQDKTEIPT
MAIPPKKNQDKTEIPTI
MAIPPKKNQDKTEIPTIN
MAIPPKKNQDKTEIPTINT

MELGHKIM
MELGHKIMRNLEN
MELGHKIMRNLENT
MELGHKIMRNLENTVKE
MELGHKIMRNLENTVKETIK
MELGHKIMRNLENTVKETIKY
MELGHKIMRNLENTVKETIKYLKS
MELGHKIMRNLENTVKETIKYLKSL
MENSAEPEQSLA
MGVSKVKEAMAPK
MHQPHQPLPPT
MHQPHQPLPPTVM
NENKVLV
NENKVLVL
NENKVLVLD
NENKVLVLDTDY
NENKVLVLDTDYK
NENKVLVLDTDYKK
NENKVLVLDTDYKKY
NENKVLVLDTDYKKYL
NENKVLVLDTDYKKYLL
NIPPLTQT
NKPEDETHL
NKVLVLDTDY
NKVLVLDTDYK
NKVLVLDTDYKK
NKVLVLDTDYKKY
NKVLVLDTDYKKYL
NKVLVLDTDYKKYLL
NLENTVK
NLENTVKET
NLENTVKETIK
NLENTVKETIKYLKS
NLENTVKETIKYLKSL
NPKLPLSIL
NQDKTEIPTINT
NSAEPEQSL
NSAEPEQSLA
PEVIESPPEINT
PMHIRL
PPKKNQDKTEIPTI
PPKKNQDKTEIPTINT
PPKKNQDKTEIPTINTI
PPKKNQDKTEIPTINTIA
PPLTQTPVVVPPFLQPEVMGVSKVKEAMAPKHKEMPF
PPLTQTPVVVPPFLQPEVMGVSKVKEAMAPKHKEMPFP

PQNIPPLTQT
PQNIPPLTQTPVVVPPFIQPEV
PVLGPVRGPFPIIV
PVVVPPF
PVVVPPFIQPEVMG
PVVVPPFIQPEVMGV
PVVVPPFIQPEVMGVSK
PVVVPPFIQPEVMGVSKVK
PVVVPPFIQPEVMGVSKVKEAMAPKHK
PVVVPPFL
PVVVPPFLQ
PVVVPPFLQP
PVVVPPFLQPE
PVVVPPFLQPEV
PVVVPPFLQPEVM
PVVVPPFLQPEVMG
PVVVPPFLQPEVMGV
PVVVPPFLQPEVMGVS
PVVVPPFLQPEVMGVSKV
PVVVPPFLQPEVMGVSKVKE
PVVVPPFLQPEVMGVSKVKEAMAPK
PVVVPPFLQPEVMGVSKVKEAMAPKH
QDKIHPF
QDKIHPFA
QDKIHPFAQT
QDKIHPFAQTQ
QDKIHPFAQTQS
QDKIHPFAQTQSL
QDKTEIPTINT
QEPVLGPVRGPFPIIV
QEPVLGPVRGPFPILV
QINNKIWCKDDQNPHSSNICNISCDKFLDDDLTDDIMCVKKILDKVGIN
QINNKIWCKDDQNPHSSNICNISCDKFLDDDLTDDIMCVKKILDKVGINY
QLKPTPEGDL
QPQSQNPKLPL
QPQSQNPKLPLS
QPQSQNPKLPLSIL
QSAPLRV
QSLVYPFPGPIHNSLPQNIPPLTQT
QSLVYPFPGPIPN
QSLVYPFPGPIPNSLPQNIPPLTQT
QSWMHQPHQPLPPT
QSWMHQPHQPLPPTV
QSWMHQPHQPLPPTVM
QTMKGLDIQKVAGT
QTMKGLDIQKVAGTW

RELEELNVPG
RELEELNVPGE
RELEELNVPGEI
RELEELNVPGEIVE
RELKDLKG
RELKDLKGY
RELKDLKGYGG
RELKDLKGYGGVS
RELKDLKGYGGVSL
RELKDLKGYGGVSLPE
RELKDLKGYGGVSLPEW
RELKDLKGYGGVSLPEWVCTTFHTSGYDTQAIVQNN
RHPEYAV
RHPYFYAPEL
RLSFNPT
RLSFNPTQ
RLSFNPTQL
RPKHPIKHQGLPQEV
RPKHPIKHQGLPQEVL
RPKHPIKHQGLPQEVLNE
RPKHPIKHQGLPQPFPEV
RTPEVDDEA
RTPEVDDEALEKFDK
RTPEVDDEALEKFDKA
RVYVEELKPTPEGD
RVYVEELKPTPEGDL
RVYVEELKPTPEGDLE
RVYVEELKPTPEGDLEI
RVYVEELKPTPEGDLEIL
RVYVEELKPTPEGDLEILL
RVYVEELKPTPEGDLEILLQ
RVYVEELKPTPEGDLEILLQK
RVYVEELKPTPEGDLEILLQKWEND
SAPLRV
SAPLRVYVEELKPTPEGDL
SAPLRVYVEELKPTPEGDLEIL
SDIPNPIGSENSEK
SDIPNPIGSENSEKT
SDIPNPIGSENSEKTTM
SDIPNPIGSENSEKTTMPL
SDISLLDAQ
SDISLLDAQSAPL
SDISLLDAQSAPLRV
SDISLLDAQSAPLRVY
SDISLLDAQSAPLRVYVEELKPTPEGD
SDISLLDAQSAPLRVYVEELKPTPEGDL

SDISLLDAQSAPLRVYVEELKPTPEGDLE
SDISLLDAQSAPLRVYVEELKPTPEGDLEIL
SEEQQQTEDELQDK
SFNPTQL
SFNPTQLEEQ
SFNPTQLEEQCHI
SGEPTSTPTIE
SGEPTSTPTIEA
SKEPSISREDL
SKVKEAMAPK
SLAMAASDISL
SLAMAASDISLL
SLAMAASDISLLD
SLAMAASDISLLDAQ
SLAMAASDISLLDAQSAPL
SLAMAASDISLLDAQSAPLRV
SLAMAASDISLLDAQSAPLRVY
SLAMAASDISLLDAQSAPLRVYVEELKPTPEGD
SLLDAQSAPLR
SLLDAQSAPLRV
SLPQNIPPLTQT
SLSQSKVLPVPEKAVPYPQ
SLSQSKVLPVPEKAVPYPQRD
SLSQSKVLPVPEKAVPYPQRDMPIQ
SLSQSKVLPVPEKAVPYPQRDMPIQA
SLSQSKVLPVPQ
SLSQSKVLPVPQK
SLSQSKVLPVPQKAVPYPQ
SLSQSKVLPVPQKAVPYPQRD
SLSQSKVLPVPQKAVPYPQRDMPIQ
SLSQSKVLPVPQKAVPYPQRDMPIQA
SLVYPFPGPIHNSLPQNIPPLTQT
SLVYPFPGPIPNSLPQNIPPLTQT
SPEVIEGPPEINT
SPEVIESPPEINT
SPPEINT
SQKFPKAEF
SRQPQSQNPKLPLSIL
SSRQPQSQNPKLPL
SSRQPQSQNPKLPLS
SSRQPQSQNPKLPLSI
SSRQPQSQNPKLPLSIL
TDVENLHLPLP
TDVENLHLPLPL
TDVENLHLPLPLL
TDVENLHLPLPLLQ

TDVENLHLPPL
TDYKKYL
TDYKKYLL
TEDELQDKIHPF
TEIPTIN
TEIPTINT
TFHTSGYDTQA
TIASGEPTSTPTIE
TIASGEPTSTPTTE
TIKYLKSLFSHA
TKIPAV
TKIPAVFKID
TLEDSPEVIESPPEINT
TLTDVENLHLPLPL
TMKGLDIQK
TMKGLDIQKVAGT
TMKGLDIQKVAGTW
TPEGDLEIL
TPEVDDEAL
TPEVDDEALE
TPEVDDEALEK
TPEVDDEALEKF
TPEVDDEALEKFD
TPEVDDEALEKFDKA
TPEVDDEALEKFDKAL
TPEVDDEALEKFDKALK
TPEVDDEALEKFDKALKALPM
TPEVDDEALEKFDKALKALPMHI
TQTMKGLDIQ
TQTMKGLDIQKVAGT
TQTMKGLDIQKVAGTW
TRKVPQVSTPT
TVKETIKYL
TVKETIKYLKS
TVKETIKYLKSL
TVKETIKYLKSLFSHA
TVMENFVAFVDK
VATLEDSPEVIESPPEINT
VDDEALEKFDK
VDDEALEKFDKA
VEELKPTPE
VEELKPTPEG
VEELKPTPEGD
VEELKPTPEGDL
VEELKPTPEGDLE
VEELKPTPEGDLEI

VEELKPTPEGDLEIL
VEELKPTPEGDLEILL
VEELKPTPEGDLEILLQ
VEELKPTPEGDLEILLQK
VEELKPTPEGDLEILLQKWEND
VEELKPTPEGDLEILLQKWENDECAQKK
VEKDAIPENLPPLTADFAEDKD
VELLKHKPK
VELLKHKPKATEEQ
VELLKHKPKATEEQL
VELLKHKPKATEEQLKT
VELLKHKPKATEEQLKTVM
VENLHLPLPL
VENLHLPLPLL
VKKILDKVGIN
VLDTDYK
VLDTDYKK
VLDTDYKKYL
VLDTDYKKYLL
VLPVPQK
VLPVPQKAVPYPQRDMPIQA
VLVLDTDYK
VLVLDTDYKK
VLVLDTDYKKY
VLVLDTDYKKYL
VLVLDTDYKKYLL
VPPFLQPEV
VPPFLQPEVM
VQVTSTAV
VRTPEVDD
VRTPEVDDE
VRTPEVDDEA
VRTPEVDDEAL
VRTPEVDDEALE
VRTPEVDDEALEK
VRTPEVDDEALEKF
VRTPEVDDEALEKFD
VRTPEVDDEALEKFDK
VRTPEVDDEALEKFDKA
VRTPEVDDEALEKFDKAL
VRTPEVDDEALEKFDKALK
VRTPEVDDEALEKFDKALKA
VRTPEVDDEALEKFDKALKALP
VRTPEVDDEALEKFDKALKALPM
VRTPEVDDEALEKFDKALKALPMH
VRTPEVDDEALEKFDKALKALPMHI

VRTPEVDDEALEKFDKALKALPMHIR
VRTPEVDDEALEKFDKALKALPMHIRL
VRTPEVDDEALEKFDKALKALPMHIRLS
VRTPEVDDEALEKFDKALKALPMHIRLSFNPT
VRTPEVDDEALEKFDKALKALPMHIRLSFNPTQ
VRTPEVDDEALEKFDKALKALPMHIRLSFNPTQL
VRTPEVDDEALEKFDKALKALPMHIRLSFNPTQLEEQCHI
VSKVKEAMAPK
VSKVKEAMAPKH
VTQTMKGLD
VTQTMKGLDIQKVAGT
VTQTMKGLDIQKVAGTW
VTQTMKGLDIQKVAGTWYSL
VVVPPFIQPEV
VYPFPGPIH
VYPFPGPIHN
VYPFPGPIHNS
VYPFPGPIHNSL
VYPFPGPIHNSLPQ
VYPFPGPIHNSLPQNIPPLT
VYPFPGPIHNSLPQNIPPLTQT
VYPFPGPIHNSLPQNIPPLTQTPVVVPPF
VYPFPGPIP
VYPFPGPIPN
VYPFPGPIPNS
VYPFPGPIPNSL
VYPFPGPIPNSLPQ
VYPFPGPIPNSLPQNIPPLT
VYPFPGPIPNSLPQNIPPLTQ
VYPFPGPIPNSLPQNIPPLTQT
VYPFPGPIPNSLPQNIPPLTQTPV
VYVEELKPTPEGDL
VYVEELKPTPEGDLE
VYVEELKPTPEGDLEI
VYVEELKPTPEGDLEIL
VYVEELKPTPEGDLEILL
VYVEELKPTPEGDLEILLQ
VYVEELKPTPEGDLEILLQK
VYVEELKPTPEGDLEILLQKWEND
WLAHKALCSEKLDQ
WMHQPHQPLPPTV
WMHQPHQPLPPTVM
WSVARLSQKFPKAEF
YPFPGPIHN
YPFPGPIHNSLPQ
YPFPGPIHNSLPQNIPPLTQT

YPFPGPIPN
YPFPGPIPNS
YPFPGPIPNSLPQNIPPLTQT
YQEPVLGPVRGPFPI
YQEPVLGPVRGPFPILV
YSLAMAASDI
YSLAMAASDIS
YSLAMAASDISL
YSLAMAASDISLL
YSLAMAASDISLLDAQ
YSLAMAASDISLLDAQSAPL
YSLAMAASDISLLDAQSAPLR
YSLAMAASDISLLDAQSAPLRV
YSLAMAASDISLLDAQSAPLRVYVEELKPTPEGDL
YSLAMAASDISLLDAQSAPLRVYVEELKPTPEGDLEIL
YVEELKPTPE
YVEELKPTPEG
YVEELKPTPEGD
YVEELKPTPEGDL
YVEELKPTPEGDLE
YVEELKPTPEGDLEI
YVEELKPTPEGDLEIL
YVEELKPTPEGDLEILL
YVEELKPTPEGDLEILLQ
YVEELKPTPEGDLEILLQK
YVEQLKPTPEGDL
YVPKAFDEK
YYANKYNGV
YYANKYNGVF

WPH.A.120min
AASDISLLDAQ
AASDISLLDAQSAPL
AASDISLLDAQSAPLRV
AASDISLLDAQSAPLRVY
AASDISLLDAQSAPLRVYVE
AASDISLLDAQSAPLRVYVEE
AASDISLLDAQSAPLRVYVEELKPTPEGD
AASDISLLDAQSAPLRVYVEELKPTPEGDL
AASDISLLDAQSAPLRVYVEELKPTPEGDLE
AASDISLLDAQSAPLRVYVEELKPTPEGDLEI
AASDISLLDAQSAPLRVYVEELKPTPEGDLEIL
AASDISLLDAQSAPLRVYVEQLKPTPEGDL

AASDISLLDAQSAPLRVYVEQLKPTPEGDLEIL
ADEKKFWG
AEKTKIPAV
AIPENLPPLTAD
AIPPKKNQD
AIPPKKNQDKTEIPTINT
AIVQNNDSTEYG
ALEKFDK
ALEKFDKA
ALEKFDKAL
ALEKFDKALK
ALEKFDKALKA
ALEKFDKALKALPM
ALEKFDKALKALPMH
ALEKFDKALKALPMHI
ALEKFDKALKALPMHIR
ALEKFDKALKALPMHIRL
ALKALPMHI
ALKALPMHIRL
ALNENKVLVL
ALNENKVLVLDTDY
ALNENKVLVLDTDYK
ALNENKVLVLDTDYKK
ALNENKVLVLDTDYKKYL
ALNENKVLVLDTDYKKYLL
ALPMHI
ALPMHIRL
ALPMHIRLSFNPT
ALPMHIRLSFNPTQL
AMAASDISLLDAQSAPL
AMAASDISLLDAQSAPLRV
AMAASDISLLDAQSAPLRVYVEELKPTPEGDL
AMAASDISLLDAQSAPLRVYVEELKPTPEGDLEIL
AQALIVTQTMK
AQSAPLRVYVEELKPTPEGDL
AQSAPLRVYVEELKPTPEGDLE
AQSAPLRVYVEELKPTPEGDLEI
AQSAPLRVYVEELKPTPEGDLEIL
ASDISLLDAQ
ASDISLLDAQSAPL
ASDISLLDAQSAPLRV
ASDISLLDAQSAPLRVYVEELKPTPEGD
ASDISLLDAQSAPLRVYVEELKPTPEGDL
ASDISLLDAQSAPLRVYVEELKPTPEGDLEI
ASDISLLDAQSAPLRVYVEELKPTPEGDLEIL
ASGEPTSTPTIE

ASGEPTSTPTIEA
ASPEVIESPPEINT
DAFLGSFLYEYSR
DAIPENLPPLTAD
DALNENKVLV
DALNENKVLVLDTDY
DAQSAPLRV
DAQSAPLRVY
DAQSAPLRVYVE
DAQSAPLRVYVEE
DAQSAPLRVYVEELKPTPEGD
DAQSAPLRVYVEELKPTPEGDL
DAQSAPLRVYVEELKPTPEGDLE
DAQSAPLRVYVEELKPTPEGDLEI
DAQSAPLRVYVEELKPTPEGDLEIL
DAQSAPLRVYVEQLKPTPEGD
DAQSAPLRVYVEQLKPTPEGDL
DDEALEKFDK
DDEALEKFDKA
DDEALEKFDKAL
DDEALEKFDKALKALPMHIRL
DEALEKFDK
DEALEKFDKA
DEALEKFDKAL
DEALEKFDKALK
DEALEKFDKALKALPM
DEALEKFDKALKALPMHI
DEALEKFDKALKALPMHIRL
DEALEKFDKALKALPMHIRLSFNPTQL
DELQDKIHP
DELQDKIHPF
DELQDKIHPFA
DELQDKIHPFAQT
DELQDKIHPFAQTQS
DELQDKIHPFAQTQSL
DISLLDAQSAPLRV
DKALKALPMHI
DKIHPFAQT
DKIHPFAQTQS
DKIHPFAQTQSL
DKLKHLVDEPQN
DKLKHLVDEPQNL
DKTEIPTINT
DKVGINYW
DLKGYGGVS
DLKGYGGVSL

DLKGYGGVSLPE
DLKGYGGVSLPEW
DLKGYGGVSLPEWV
DLSKEPSISRE
DLSKEPSISREDL
DSPEVIEGPPEINT
DSPEVIESPPEINT
DTDYKKYL
DTDYKKYLL
DTHKSEIAHRFKDLGEEHFKG
DVENLHLPLP
DVENLHLPLPL
EALEKFDK
EALEKFDKA
EALEKFDKAL
EALEKFDKALK
EALEKFDKALKALPM
EALEKFDKALKALPMHI
EALEKFDKALKALPMHIRL
EALEKFDKALKALPMHIRLSFNPTQL
EAMAPKHKEMPFPK
EAMAPKHKEMPFPKYPVE
EAMAPKHKEMPFPKYPVEPF
EAMAPKHKEMPFPKYPVEPFTESQS
EAQPTDASAQF
EASPEVIESPPEIN
EDSPEVIEGPPEINT
EDSPEVIESPPEINT
EELKPTPEGD
EELKPTPEGDL
EELKPTPEGDLE
EELKPTPEGDLEI
EELKPTPEGDLEIL
EKFDKAL
EKFDKALK
EKFDKALKA
EKFDKALKALP
EKFDKALKALPM
EKFDKALKALPMH
EKFDKALKALPMHI
EKFDKALKALPMHIR
EKFDKALKALPMHIRL
ELEELNVPGE
ELEELNVPGEIVE
ELGHKIM
ELKDLKG

ELKDLKGYGG
ELKDLKGYGGVS
ELKDLKGYGGVSL
ELKDLKGYGGVSLPEWV
ELKPTPEGD
ELKPTPEGDL
ELKPTPEGDLE
ELKPTPEGDLEI
ELKPTPEGDLEIL
ELQDKIHPF
ELQDKIHPFAQTQSL
EMPFPKYPVE
EMPFPKYPVEPF
EMPFPKYPVEPFTESQ
EMPFPKYPVEPFTESQS
ENSAEPEQSLA
EPVLGPVRGPFPIIV
ESPPEINT
EVDDEALEK
EVDDEALEKFDK
EVDDEALEKFDKA
EVIESPPEINT
FAQTQSLVYPFPGP
FDKALKALPM
FDKALKALPMHI
FDKLKHLVDEPQNL
FEVVKT
FHTSGYDTQA
FKDLGEEH
FKDLGEEHFKGLV
FKIDALNE
FKIDALNENKVLV
FMAIPPKKNQDKTEIPTINT
FPGPIPNSLPQNIPPLTQT
FQINNKIW
FQINNKIWCKDDQNPHSSNICNISCDKFLDDDLTDDIMCVKKILDKVGIN
FQSEEQQQTEDELQDKIHP
FVAPFPEV
FVAPFPEVFG
FVAPFPEVFGK
FVAPFPEVFGKE
FVAPFPEVFGKEK
FVAPFPEVFGKEKVNE
FVAPFPEVFGKEKVNEL
GEPTSTPTIE
GGVSLPEW

GGVSLPEWV
GLDIQK
GLDIQKV
GLDIQKVA
GLDIQKVAGT
GLDIQKVAGTW
GLDIQKVAGTWY
GLDIQKVAGTWYSLA
GLDIQKVAGTWYSLAM
GLDIQKVAGTWYSLAMA
GPIHNSLPQNIPPLTQT
GVSKVKEAM
GVSKVKEAMAP
GVSKVKEAMAPK
GVSKVKEAMAPKH
GVSKVKEAMAPKHK
GVSKVKEAMAPKHKEMPFPK
GVSKVKEAMAPKHKEMPFPKYPVE
GVSKVKEAMAPKHKEMPFPKYPVEPF
GVSKVKEAMAPKHKEMPFPKYPVEPFT
GVSKVKEAMAPKHKEMPFPKYPVEPFTES
GVSKVKEAMAPKHKEMPFPKYPVEPFTESQ
GVSKVKEAMAPKHKEMPFPKYPVEPFTESQS
GVSKVKEAMAPKHKEMPFPKYPVEPFTESQSL
GVSKVKEAMAPKHKEMPFPKYPVEPFTESQSLT
GVSKVKEAMAPKHKEMPFPKYPVQPFTESQS
GYGGVSLPEWV
HIQKEDVPSE
HIQKEDVPSER
HIQKEDVPSERYLG
HIRLSFNPT
HIRLSFNPTQ
HIRLSFNPTQL
HKEMPFPK
HKEMPFPKYPVE
HKEMPFPKYPVEP
HKEMPFPKYPVEPF
HKEMPFPKYPVEPFTES
HKEMPFPKYPVEPFTESQS
HLVDEPQN
HLVDEPQNL
HNSLPQNIPPLTQT
HPYFYAPEL
HQGLPQPFPEV
HQPHQPLPPT
HQPHQPLPPTVM

HVKLVNE
IAEKTKIPA
IAEKTKIPAV
IASGEPTSTPITE
IASGEPTSTPTIE
IASGEPTSTPTIEA
IASGEPTSTPTTE
IASGEPTSTPTTEA
ICNISCDKFLDDDLTDDIMCVKKILDKVGIN
IDALNENK
IDALNENKV
IDALNENKVL
IDALNENKVLV
IDALNENKVLVLDTDY
IDALNENKVLVLDTDYK
IDALNENKVLVLDTDYKK
IESPPEINT
IHPFAQTQS
IHPFAQTQSL
IIAEKTKIPA
IIAEKTKIPAV
IIAEKTKIPAVFKID
IIAEKTKIPAVFKIDA
IIAEKTKIPAVFKIDALNENKVLV
IIAEKTKIPAVFKIDALNENKVLVLDTDYK
IIVTQTM
IIVTQTMKG
IIVTQTMKGL
IIVTQTMKGLDI
IIVTQTMKGLDIQ
IIVTQTMKGLDIQK
IIVTQTMKGLDIQKV
IIVTQTMKGLDIQKVA
IIVTQTMKGLDIQKVAGT
IIVTQTMKGLDIQKVAGTW
IIVTQTMKGLDIQKVAGTWYSLAM
IIVTQTMKGLDIQKVAGTWYSLAMAA
IIVTQTMKGLDIQKVAGTWYSLAMAASD
IIVTQTMKGLDIQKVAGTWYSLAMAASDISLLD
IIVTQTMKGLDIQKVAGTWYSLAMAASDISLLDAQSAPLR
ILDKVGIN
ILDKVGINY
ILNKPEDET
ILNKPEDETH
ILNKPEDETHL
IMCVKKILDKVGIN

IPAVFKIDALNENKVLVLDTDYK
IPPKKNQDKTEIPT
IPPKKNQDKTEIPTINT
IQKEDVPSER
IQKVAGTW
IQKVAGTWYSLA
IRNLQISNE
IRNLQISNEDL
ISKEQIVI
ISKEQIVIRSS
ISLLDAQSAPLR
ISLLDAQSAPLRV
ISLLDAQSAPLRVYVE
ISLLDAQSAPLRVYVEELKPTPEGD
ISLLDAQSAPLRVYVEELKPTPEGDL
ISLLDAQSAPLRVYVEELKPTPEGDLE
ISLLDAQSAPLRVYVEELKPTPEGDLEIL
IVQNNDSTEY
IVQNNDSTEYG
IVQNNDSTEYGL
IVRYTRKVPQVSTPT
IVTQTMKG
IVTQTMKGL
IVTQTMKGLD
IVTQTMKGLDIQ
IVTQTMKGLDIQK
IVTQTMKGLDIQKVA
IVTQTMKGLDIQKVAGT
IVTQTMKGLDIQKVAGTW
IVTQTMKGLDIQKVAGTWYSL
IVTQTMKGLDIQKVAGTWYSLA
KADEKKFWG
KALKALPM
KALKALPMHI
KALKALPMHIRL
KALPMHI
KDLKGYGGVSL
KFDKALKALPM
KFDKALKALPMHI
KFLDDDL
KFLDDDLTDD
KGLDIQK
KGLDIQKVAGT
KGLDIQKVAGTW
KIDALNE
KIDALNEN

KIDALNENK
KIDALNENKV
KIDALNENKVL
KIDALNENKVLV
KIDALNENKVLVL
KIDALNENKVLVLD
KIDALNENKVLVLDT
KIDALNENKVLVLDTD
KIDALNENKVLVLDTDY
KIDALNENKVLVLDTDYK
KIDALNENKVLVLDTDYKK
KIDALNENKVLVLDTDYKKY
KIDALNENKVLVLDTDYKKYL
KIDALNENKVLVLDTDYKKYLL
KIDALNENKVLVLDTDYKKYLLF
KIDALNENKVLVLDTDYKKYLLFCMENSAEPEQSLVCQ
KIDALNENKVLVLDTDYKKYLLFCMENSAEPEQSLVCQCL
KIEKFQSEEQQQTEDEL
KIEKFQSEEQQQTEDELQDKIHP
KIEKFQSEEQQQTEDELQDKIHPF
KIEKFQSEEQQQTEDELQDKIHPFAQTQSL
KIHPFAQT
KIHPFAQTQ
KIHPFAQTQS
KIHPFAQTQSL
KIIAEKTKIPA
KIIAEKTKIPAV
KILDKVGIN
KNQDKTEIPT
KNQDKTEIPTINT
KPTPEGDLE
KPTPEGDLEI
KPTPEGDLEIL
KPTPEGDLEILL
KTEIPTINT
KTKIPAV
KVPQVSTPT
LDAQSAPLR
LDAQSAPLRV
LDAQSAPLRVY
LDAQSAPLRVYVE
LDAQSAPLRVYVEE
LDAQSAPLRVYVEELKPTPEGD
LDAQSAPLRVYVEELKPTPEGDL
LDAQSAPLRVYVEELKPTPEGDLE
LDAQSAPLRVYVEELKPTPEGDLEI

LDAQSAPLRVYVEELKPTPEGDLEIL
LDAQSAPLRVYVEQLKPTPEGDLEIL
LDIQKVAGT
LDIQKVAGTW
LDKVGIN
LEDSPEVIESPPEINT
LEKFDK
LEKFDKA
LEKFDKAL
LEKFDKALK
LEKFDKALKALPM
LEKFDKALKALPMH
LEKFDKALKALPMHI
LEKFDKALKALPMHIRL
LEKFDKALKALPMHIRLSFNPTQL
LIVTQTMK
LIVTQTMKGL
LIVTQTMKGLD
LIVTQTMKGLDI
LIVTQTMKGLDIQK
LIVTQTMKGLDIQKVAG
LIVTQTMKGLDIQKVAGTWY
LIVTQTMKGLDIQKVAGTWYS
LIVTQTMKGLDIQKVAGTWYSL
LIVTQTMKGLDIQKVAGTWYSLA
LIVTQTMKGLDIQKVAGTWYSLAM
LIVTQTMKGLDIQKVAGTWYSLAMA
LIVTQTMKGLDIQKVAGTWYSLAMAASD
LIVTQTMKGLDIQKVAGTWYSLAMAASDISL
LIVTQTMKGLDIQKVAGTWYSLAMAASDISLL
LKDLKGYGGVS
LKDLKGYGGVSL
LKPTPEGDL
LKPTPEGDLE
LKPTPEGDLEIL
LLDAQSAPLRV
LNENKVLV
LNENKVLVLDTDYKKYL
LNKPEDETHL
LPKLKPD
LPMHIRL
LPQNIPPLTQT
LQDKIHP
LQDKIHPF
LQDKIHPFAQ
LQDKIHPFAQT

LQDKIHPFAQTQ
LQDKIHPFAQTQS
LQDKIHPFAQTQSL
LQKWENGECAQKKIIAEKTKIPAV
LRLAKEYEAT
LSFNPTQL
LSFNPTQLEEQ
LSFNPTQLEEQC
LSFNPTQLEEQCH
LSFNPTQLEEQCHI
LSQKFPKAEF
LTDVENLHLPLPL
LTDVENLHLPLPLL
LVLDTDYK
LVLDTDYKK
LVLDTDYKKYLL
LVRTPEVDDEALEKFDKALKALPMHI
LVYPFPGPIHN
LVYPFPGPIHNSLPQNIPPLTQT
LVYPFPGPIPN
LVYPFPGPIPNS
LVYPFPGPIPNSLPQNIPPLTQT
LYQEPVLGPVRGPFPIIV
LYYANKYNGV
MAASDISLLDAQ
MAASDISLLDAQSAPL
MAASDISLLDAQSAPLRV
MAASDISLLDAQSAPLRVYVE
MAASDISLLDAQSAPLRVYVEELKPTPEGDL
MAASDISLLDAQSAPLRVYVEELKPTPEGDLE
MAASDISLLDAQSAPLRVYVEELKPTPEGDLEIL
MAIPPK
MAIPPKK
MAIPPKKNQD
MAIPPKKNQDK
MAIPPKKNQDKTEIPT
MAIPPKKNQDKTEIPTIN
MAIPPKKNQDKTEIPTINT
MELGHKIM
MELGHKIMRNLEN
MELGHKIMRNLENT
MELGHKIMRNLENTVKE
MELGHKIMRNLENTVKETIK
MELGHKIMRNLENTVKETIKY
MELGHKIMRNLENTVKETIKYLKS
MELGHKIMRNLENTVKETIKYLKSL

MENSAEPEQSL
MENSAEPEQSLA
MGVSKVKEAMAPK
MHQPHQPLPPT
MHQPHQPLPPTVM
NENKVLV
NENKVLVL
NENKVLVLD
NENKVLVLDTDY
NENKVLVLDTDYK
NENKVLVLDTDYKK
NENKVLVLDTDYKKY
NENKVLVLDTDYKKYL
NENKVLVLDTDYKKYLL
NIPPLTQT
NKPEDETHL
NKVLVLDTDY
NKVLVLDTDYK
NKVLVLDTDYKK
NKVLVLDTDYKKYL
NKVLVLDTDYKKYLL
NLENTVK
NLENTVKET
NLENTVKETIK
NLENTVKETIKYLKS
NLENTVKETIKYLKSL
NPKLPLSIL
NQDKTEIPTINT
NSAEPEQSL
NSAEPEQSLA
PEVIESPPEINT
PPKKNQDKTEIPTI
PPKKNQDKTEIPTINT
PPKKNQDKTEIPTINTI
PPKKNQDKTEIPTINTIA
PQNIPPLTQT
PQNIPPLTQTPVVVPPFIQPEV
PVLGPVRGPFPIIV
PVVVPPF
PVVVPPFIQPEVMG
PVVVPPFIQPEVMGV
PVVVPPFIQPEVMGVSK
PVVVPPFIQPEVMGVSKVK
PVVVPPFIQPEVMGVSKVKEAMAPKHK
PVVVPPFL
PVVVPPFLQ

PVVVPPFLQP
PVVVPPFLQPE
PVVVPPFLQPEV
PVVVPPFLQPEVM
PVVVPPFLQPEVMG
PVVVPPFLQPEVMGV
PVVVPPFLQPEVMGVS
PVVVPPFLQPEVMGVSKV
PVVVPPFLQPEVMGVSKVKE
PVVVPPFLQPEVMGVSKVKEAMAPK
PVVVPPFLQPEVMGVSKVKEAMAPKH
QDKIHPF
QDKIHPFA
QDKIHPFAQT
QDKIHPFAQTQ
QDKIHPFAQTQS
QDKIHPFAQTQSL
QDKTEIPTINT
QEPVLGPVRGPFPIIV
QEPVLGPVRGPFPILV
QINNKIWCKDDQNPHSSNICNISCDKFLDDDLTDDIMCVKKILDKVGIN
QINNKIWCKDDQNPHSSNICNISCDKFLDDDLTDDIMCVKKILDKVGINY
QLKPTPEGDL
QPQSQNPKLPL
QPQSQNPKLPLS
QPQSQNPKLPLSIL
QSAPLRV
QSLVYPFPGPIHNSLPQNIPPLTQT
QSLVYPFPGPIPN
QSLVYPFPGPIPNSLPQNIPPLTQT
QSWMHQPHQPLPPT
QSWMHQPHQPLPPTV
QSWMHQPHQPLPPTVM
QTMKGLDIQKVAGT
QTMKGLDIQKVAGTW
RELEELNVPG
RELEELNVPGE
RELEELNVPGEI
RELEELNVPGEIVE
RELKDLKG
RELKDLKGY
RELKDLKGYGG
RELKDLKGYGGVS
RELKDLKGYGGVSL
RELKDLKGYGGVSLPE
RELKDLKGYGGVSLPEW

RELKDLKGYGGVSLPEWVCTTFHTSGYDTQAIVQNN
RHPEYAV
RHPYFYAPEL
RLSFNPT
RLSFNPTQ
RLSFNPTQL
RPKHPIKHQGLPQEV
RPKHPIKHQGLPQEVL
RPKHPIKHQGLPQEVLNE
RPKHPIKHQGLPQPFPEV
RTPEVDDEA
RTPEVDDEALEKFDK
RTPEVDDEALEKFDKA
RVYVEELKPTPEGD
RVYVEELKPTPEGDL
RVYVEELKPTPEGDLE
RVYVEELKPTPEGDLEI
RVYVEELKPTPEGDLEIL
RVYVEELKPTPEGDLEILL
RVYVEELKPTPEGDLEILLQ
RVYVEELKPTPEGDLEILLQKWEND
SAEPEQSL
SAPLRV
SAPLRVYVEELKPTPEGDL
SAPLRVYVEELKPTPEGDLEIL
SDIPNPIGSENSEK
SDIPNPIGSENSEKT
SDIPNPIGSENSEKTTM
SDIPNPIGSENSEKTTMPL
SDISLLDAQ
SDISLLDAQSAPL
SDISLLDAQSAPLRV
SDISLLDAQSAPLRVY
SDISLLDAQSAPLRVYVEELKPTPEGD
SDISLLDAQSAPLRVYVEELKPTPEGDL
SDISLLDAQSAPLRVYVEELKPTPEGDLE
SDISLLDAQSAPLRVYVEELKPTPEGDLEIL
SEEQQQTEDELQDK
SFNPTQL
SFNPTQLEEQ
SFNPTQLEEQCHI
SGEPTSTPTIE
SGEPTSTPTIEA
SKEPSISREDL
SKVKEAMAPK
SLAMAASDISL

SLAMAASDISLL
SLAMAASDISLLD
SLAMAASDISLLDAQ
SLAMAASDISLLDAQSAPL
SLAMAASDISLLDAQSAPLRV
SLAMAASDISLLDAQSAPLRVY
SLAMAASDISLLDAQSAPLRVYVEELKPTPEGD
SLLDAQSAPLRV
SLPQNIPPLTQT
SLSQSKVLPVPEKAVPYPQ
SLSQSKVLPVPEKAVPYPQRD
SLSQSKVLPVPEKAVPYPQRDMPIQ
SLSQSKVLPVPEKAVPYPQRDMPIQA
SLSQSKVLPVPQ
SLSQSKVLPVPQK
SLSQSKVLPVPQKAVPYPQ
SLSQSKVLPVPQKAVPYPQRD
SLSQSKVLPVPQKAVPYPQRDMPIQ
SLSQSKVLPVPQKAVPYPQRDMPIQA
SLVYPFPGPIHNSLPQNIPPLTQT
SLVYPFPGPIPNSLPQNIPPLTQT
SPEVIEGPPEINT
SPEVIESPPEINT
SPPEINT
SQKFPKAEF
SRQPQSQNPKLPLSIL
SSRQPQSQNPKLPL
SSRQPQSQNPKLPLS
SSRQPQSQNPKLPLSI
SSRQPQSQNPKLPLSIL
TDVENLHLPLP
TDVENLHLPLPL
TDVENLHLPLPLL
TDVENLHLPLPLLQ
TDVENLHLPPL
TDYKKYL
TDYKKYLL
TEDELQDKIHPF
TEIPTIN
TEIPTINT
TFHTSGYDTQA
TIASGEPTSTPTIE
TIASGEPTSTPTTE
TKIPAV
TKIPAVFKID
TLEDSPEVIESPPEINT

TLTDVENLHLPLPL
TMKGLDIQK
TMKGLDIQKVAGT
TMKGLDIQKVAGTW
TPEGDLEIL
TPEVDDEAL
TPEVDDEALEK
TPEVDDEALEKF
TPEVDDEALEKFD
TPEVDDEALEKFDKA
TPEVDDEALEKFDKAL
TPEVDDEALEKFDKALK
TPEVDDEALEKFDKALKALPM
TPEVDDEALEKFDKALKALPMHI
TPVVVPPFIQPEVM
TQTMKGLDIQ
TQTMKGLDIQKVAGT
TRKVPQVSTPT
TVKETIKYL
TVKETIKYLKS
TVKETIKYLKSL
TVKETIKYLKSLFSHA
TVMENFVAFVDK
VATLEDSPEVIESPPEINT
VDDEALEKFDK
VDDEALEKFDKA
VEELKPTPE
VEELKPTPEG
VEELKPTPEGD
VEELKPTPEGDL
VEELKPTPEGDLE
VEELKPTPEGDLEI
VEELKPTPEGDLEIL
VEELKPTPEGDLEILL
VEELKPTPEGDLEILLQ
VEELKPTPEGDLEILLQK
VEELKPTPEGDLEILLQKWEND
VEELKPTPEGDLEILLQKWENDECAQKK
VEKDAIPENLPPLTADFAEDKD
VELLKHKPK
VELLKHKPKATEEQ
VELLKHKPKATEEQL
VELLKHKPKATEEQLKT
VELLKHKPKATEEQLKTVM
VENLHLPLPL
VKKILDKVGIN

VLDTDYK
VLDTDYKK
VLDTDYKKYL
VLDTDYKKYLLF
VLPVPQK
VLPVPQKAVPYPQRDMPIQA
VLVLDTDYK
VLVLDTDYKK
VLVLDTDYKKYL
VPPFLQPEV
VPPFLQPEVM
VQVTSTAV
VRTPEVDD
VRTPEVDDE
VRTPEVDDEA
VRTPEVDDEAL
VRTPEVDDEALE
VRTPEVDDEALEK
VRTPEVDDEALEKFD
VRTPEVDDEALEKFDK
VRTPEVDDEALEKFDKA
VRTPEVDDEALEKFDKAL
VRTPEVDDEALEKFDKALK
VRTPEVDDEALEKFDKALKA
VRTPEVDDEALEKFDKALKALP
VRTPEVDDEALEKFDKALKALPM
VRTPEVDDEALEKFDKALKALPMH
VRTPEVDDEALEKFDKALKALPMHI
VRTPEVDDEALEKFDKALKALPMHIR
VRTPEVDDEALEKFDKALKALPMHIRL
VRTPEVDDEALEKFDKALKALPMHIRLS
VRTPEVDDEALEKFDKALKALPMHIRLSFNPT
VRTPEVDDEALEKFDKALKALPMHIRLSFNPTQ
VRTPEVDDEALEKFDKALKALPMHIRLSFNPTQL
VRTPEVDDEALEKFDKALKALPMHIRLSFNPTQLEEQCHI
VSKVKEAMAPK
VSKVKEAMAPKH
VSKVKEAMAPKHK
VTQTMKGLD
VTQTMKGLDIQKVAGT
VTQTMKGLDIQKVAGTW
VTQTMKGLDIQKVAGTWYSL
VVVPPFIQPEV
VYPFPGPIH
VYPFPGPIHN
VYPFPGPIHNS

VYPFPGPIHNSL
VYPFPGPIHNSLPQ
VYPFPGPIHNSLPQNIPPLT
VYPFPGPIHNSLPQNIPPLTQT
VYPFPGPIHNSLPQNIPPLTQTPVVVPPF
VYPFPGPIP
VYPFPGPIPN
VYPFPGPIPNS
VYPFPGPIPNSL
VYPFPGPIPNSLPQ
VYPFPGPIPNSLPQNIPPLT
VYPFPGPIPNSLPQNIPPLTQ
VYPFPGPIPNSLPQNIPPLTQT
VYPFPGPIPNSLPQNIPPLTQTPV
VYVEELKPTPEGDL
VYVEELKPTPEGDLE
VYVEELKPTPEGDLEI
VYVEELKPTPEGDLEIL
VYVEELKPTPEGDLEILL
VYVEELKPTPEGDLEILLQ
VYVEELKPTPEGDLEILLQK
VYVEELKPTPEGDLEILLQKWEND
WLAHKALCSEKLDQ
WMHQPHQPLPPTV
WMHQPHQPLPPTVM
WSVARLSQKFPKAEF
YPFPGPIHN
YPFPGPIHNSLPQ
YPFPGPIHNSLPQNIPPLTQT
YPFPGPIPN
YPFPGPIPNS
YPFPGPIPNSLPQNIPPLTQT
YQEPVLGPVRGPFPI
YQEPVLGPVRGPFPILV
YSLAMAASDI
YSLAMAASDIS
YSLAMAASDISL
YSLAMAASDISLL
YSLAMAASDISLLDAQ
YSLAMAASDISLLDAQSAPL
YSLAMAASDISLLDAQSAPLR
YSLAMAASDISLLDAQSAPLRV
YVEELKPTPE
YVEELKPTPEG
YVEELKPTPEGD
YVEELKPTPEGDL

YVEELKPTPEGDLE
YVEELKPTPEGDLEI
YVEELKPTPEGDLEIL
YVEELKPTPEGDLEILL
YVEELKPTPEGDLEILLQ
YVEELKPTPEGDLEILLQK
YVEQLKPTPEGDL
YVPKAFDEK
YVPKAFDEKL
YYANKYNGV

WPH.B.120min
AASDISLLDAQSAPL
AASDISLLDAQSAPLRVYVEELKPTPEGD
AASDISLLDAQSAPLRVYVEELKPTPEGDLE
AASDISLLDAQSAPLRVYVEQLKPTPEGDL
AASDISLLDAQSAPLRVYVEQLKPTPEGDLEIL
AIPPKKNQDKTEIPTINT
AIVQNNDSTEYG
ALEKFDK
ALEKFDKA
ALEKFDKALKA
ALEKFDKALKALPMHI
ALNENKVLVLDTDYKKYL
ALPMHI
ALPMHIRLSFNPTQL
AMAASDISLLDAQSAPLRV
AMAASDISLLDAQSAPLRVYVEELKPTPEGDL
AQSAPLRVYVEELKPTPEGDL
AQSAPLRVYVEELKPTPEGDLE
AQSAPLRVYVEELKPTPEGDLEIL
ASDISLLDAQ
ASDISLLDAQSAPL
ASDISLLDAQSAPLRVYVEELKPTPEGDLEIL
ASGEPTSTPTIE
ASGEPTSTPTIEA
ASPEVIESPPEINT
DAFLGSFLYEYSR
DAIPENLPPLTAD
DALNENKVLV
DALNENKVLVLDTDY
DAQSAPLRVYVE
DAQSAPLRVYVEELKPTPEGD

DAQSAPLRVYVEELKPTPEGDL
DAQSAPLRVYVEELKPTPEGDLE
DAQSAPLRVYVEELKPTPEGDLEI
DAQSAPLRVYVEELKPTPEGDLEIL
DAQSAPLRVYVEQLKPTPEGDL
DDEALEKFDK
DEALEKFDK
DEALEKFDKA
DEALEKFDKAL
DEALEKFDKALK
DEALEKFDKALKALPMHI
DEALEKFDKALKALPMHIRLSFNPTQL
DELQDKIHP
DELQDKIHPF
DELQDKIHPFAQTQS
DISLLDAQSAPLRV
DKIHPFAQT
DKTEIPTINT
DLKGYGGVS
DLKGYGGVSLPE
DLKGYGGVSLPEWV
DSPEVIEGPPEINT
DSPEVIESPPEINT
DTHKSEIAHRFKDLGEEHFKG
DVENLHLPLP
DVENLHLPLPL
EALEKFDK
EALEKFDKALK
EALEKFDKALKALPM
EALEKFDKALKALPMHIRL
EAMAPKHKEMPFPK
EAMAPKHKEMPFPKYPVE
EAMAPKHKEMPFPKYPVEPF
EASPEVIESPPEIN
EDSPEVIEGPPEINT
EDSPEVIESPPEINT
EELKPTPEGD
EELKPTPEGDL
EELKPTPEGDLE
EELKPTPEGDLEI
EELKPTPEGDLEIL
EKFDKALKALPMHI
ELEELNVPGE
ELEELNVPGEIVE
ELKDLKG
ELKDLKGYGGVS

ELKPTPEGDL
ELKPTPEGDLE
ELKPTPEGDLEI
ELKPTPEGDLEIL
EMPFPKYPVEPF
EMPFPKYPVEPFTESQ
ESPPEINT
EVDDEALEK
EVDDEALEKFDK
EVDDEALEKFDKA
EVIESPPEINT
FMAIPPKKNQDKTEIPTINT
FVAPFPEV
GEPTSTPTIE
GLDIQK
GLDIQKV
GLDIQKVA
GLDIQKVAGT
GLDIQKVAGTWY
GLDIQKVAGTWYSLAMA
GVSKVKEAMAPK
GVSKVKEAMAPKHKEMPFPKYPVE
GVSKVKEAMAPKHKEMPFPKYPVEPFTESQS
GVSKVKEAMAPKHKEMPFPKYPVQPFTESQS
HKEMPFPKYPVEPF
HLVDEPQNL
IAEKTKIPA
IAEKTKIPAV
IASGEPTSTPITE
IASGEPTSTPTIE
IASGEPTSTPTIEA
IASGEPTSTPTTE
IASGEPTSTPTTEA
IDALNENK
IDALNENKV
IDALNENKVL
IDALNENKVLV
IDALNENKVLVLDTDY
IDALNENKVLVLDTDYK
IDALNENKVLVLDTDYKK
IESPPEINT
IIAEKTKIPA
IIAEKTKIPAV
IIAEKTKIPAVFKIDA
IIVTQTMKGLDIQ
IIVTQTMKGLDIQKVAGT

IIVTQTMKGLDIQKVAGTW
IIVTQTMKGLDIQKVAGTWYSLAM
IIVTQTMKGLDIQKVAGTWYSLAMAASDISLLD
IIVTQTMKGLDIQKVAGTWYSLAMAASDISLLDAQSAPLR
ILDKVGIN
ILNKPEDET
ILNKPEDETHL
IPPKKNQDKTEIPT
IPPKKNQDKTEIPTINT
IQKVAGTWYSL
ISKEQIVIRSS
ISLLDAQSAPLRVYVEELKPTPEGD
ISLLDAQSAPLRVYVEELKPTPEGDL
IVQNNDSTEYG
IVTQTMKGLDIQ
KADEKKFWGKY
KDLKGYGGVSL
KFLDDDLTDD
KGLDIQKVAGT
KIDALNENK
KIDALNENKV
KIDALNENKVL
KIDALNENKVLV
KIDALNENKVLVLDTD
KIDALNENKVLVLDTDY
KIDALNENKVLVLDTDYK
KIDALNENKVLVLDTDYKK
KIDALNENKVLVLDTDYKKYLLF
KIEKFQSEEQQQTEDEL
KIHPFAQTQSL
KIIAEKTKIPA
KILDKVGIN
KNQDKTEIPT
KNQDKTEIPTINT
KPTPEGDLEI
KTEIPTINT
KVPQVSTPT
LDAQSAPLRVYVEE
LDAQSAPLRVYVEELKPTPEGDL
LDAQSAPLRVYVEELKPTPEGDLE
LDAQSAPLRVYVEELKPTPEGDLEI
LDAQSAPLRVYVEELKPTPEGDLEIL
LDAQSAPLRVYVEQLKPTPEGDLEIL
LDIQKVAGT
LDKVGIN
LEDSPEVIESPPEINT

LEKFDKA
LEKFDKALKALPMHIR
LIVTQTMKGLD
LIVTQTMKGLDIQKVAGTWY
LIVTQTMKGLDIQKVAGTWYSL
LIVTQTMKGLDIQKVAGTWYSLAM
LIVTQTMKGLDIQKVAGTWYSLAMA
LIVTQTMKGLDIQKVAGTWYSLAMAASD
LIVTQTMKGLDIQKVAGTWYSLAMAASDISLL
LKALPMHIRL
LKDLKGYGGVS
LKPTPEGDL
LPMHIRL
LQDKIHP
LQDKIHPFAQT
LQDKIHPFAQTQ
LQDKIHPFAQTQS
LQDKIHPFAQTQSL
LSFNPTQLEEQCH
LSFNPTQLEEQCHI
LVYPFPGPIHNSLPQNIPPLTQT
LVYPFPGPIPN
LVYPFPGPIPNSLPQNIPPLTQT
MAASDISLLDAQSAPL
MAASDISLLDAQSAPLRVYVEELKPTPEGDL
MAASDISLLDAQSAPLRVYVEELKPTPEGDLE
MAIPPK
MAIPPKKNQD
MAIPPKKNQDKTEIPT
MAIPPKKNQDKTEIPTIN
MAIPPKKNQDKTEIPTINT
MELGHKIMRNLENTVKETIKYLKS
MELGHKIMRNLENTVKETIKYLKSL
MHQPHQPLPPTVM
NENKVLVL
NENKVLVLDTDY
NKVLVLDTDYKK
NKVLVLDTDYKKYL
NLENTVKET
NLENTVKETIK
NQDKTEIPTINT
PEVIESPPEINT
PPKKNQDKTEIPTI
PPKKNQDKTEIPTINT
PPKKNQDKTEIPTINTI
PPKKNQDKTEIPTINTIA

PPLTQTPVVVPPFLQPEVMGVSKVKEAMAPKHKEMPF
PPLTQTPVVVPPFLQPEVMGVSKVKEAMAPKHKEMPFP
PQNIPPLTQTPVVVPPFIQPEV
PVVVPPF
PVVVPPFIQPEVMG
PVVVPPFIQPEVMGV
PVVVPPFIQPEVMGVSK
PVVVPPFLQPE
PVVVPPFLQPEVMGV
PVVVPPFLQPEVMGVS
PVVVPPFLQPEVMGVSKVKE
PVVVPPFLQPEVMGVSKVKEAMAPK
PVVVPPFLQPEVMGVSKVKEAMAPKH
QDKIHPFAQT
QDKIHPFAQTQS
QDKIHPFAQTQSL
QDKTEIPTINT
QEPVLGPVRGPFPILV
QLKPTPEGDL
QPQSQNPKLPL
QPQSQNPKLPLS
QSLVYPFPGPIPNSLPQNIPPLTQT
QSWMHQPHQPLPPT
RELEELNVPGE
RELEELNVPGEIVE
RELKDLKG
RELKDLKGYGGVSLPE
RTPEVDDEALEKFDK
RTPEVDDEALEKFDKA
RVYVEELKPTPEGDL
RVYVEELKPTPEGDLE
RVYVEELKPTPEGDLEI
RVYVEELKPTPEGDLEIL
RVYVEELKPTPEGDLEILLQ
RVYVEELKPTPEGDLEILLQK
SAPLRVYVEELKPTPEGDLEIL
SDIPNPIGSENSEKT
SDISLLDAQSAPL
SDISLLDAQSAPLRVYVEELKPTPEGD
SDISLLDAQSAPLRVYVEELKPTPEGDLE
SEEQQQTEDELQDK
SFNPTQLEEQ
SGEPTSTPTIE
SKVKEAMAPK
SLAMAASDISLLDAQ
SLAMAASDISLLDAQSAPL

SLAMAASDISLLDAQSAPLRVY
SLPQNIPPLTQT
SLVYPFPGPIHNSLPQNIPPLTQT
SLVYPFPGPIPNSLPQNIPPLTQT
SPEVIEGPPEINT
SPEVIESPPEINT
SPPEINT
SQKFPKAEF
SSRQPQSQNPKLPL
TDVENLHLPLPL
TDVENLHLPLPLL
TDVENLHLPLPLLQ
TDVENLHLPPL
TEIPTIN
TEIPTINT
TIASGEPTSTPTTE
TKIPAV
TLEDSPEVIESPPEINT
TMKGLDIQKVAGT
TMKGLDIQKVAGTW
TPEVDDEALEKF
TPEVDDEALEKFD
TPEVDDEALEKFDKA
TPEVDDEALEKFDKAL
TPEVDDEALEKFDKALKALPMHI
TPVVVPPFIQPEVM
TQTMKGLDIQKVAGT
TRKVPQVSTPT
TVMENFVAFVDK
VEELKPTPEGD
VEELKPTPEGDL
VEELKPTPEGDLE
VEELKPTPEGDLEI
VEELKPTPEGDLEIL
VEELKPTPEGDLEILL
VEELKPTPEGDLEILLQKWEND
VEELKPTPEGDLEILLQKWENDECAQKK
VENLHLPLPL
VKKILDKVGIN
VLDTDYK
VLDTDYKKYL
VLVLDTDYK
VPPFLQPEV
VQVTSTAV
VRTPEVDDE
VRTPEVDDEA

VRTPEVDDEAL
VRTPEVDDEALE
VRTPEVDDEALEK
VRTPEVDDEALEKFD
VRTPEVDDEALEKFDK
VRTPEVDDEALEKFDKA
VRTPEVDDEALEKFDKAL
VRTPEVDDEALEKFDKALK
VRTPEVDDEALEKFDKALKA
VRTPEVDDEALEKFDKALKALP
VRTPEVDDEALEKFDKALKALPM
VRTPEVDDEALEKFDKALKALPMH
VRTPEVDDEALEKFDKALKALPMHI
VRTPEVDDEALEKFDKALKALPMHIRLS
VRTPEVDDEALEKFDKALKALPMHIRLSFNPTQ
VRTPEVDDEALEKFDKALKALPMHIRLSFNPTQL
VSKVKEAMAPK
VSKVKEAMAPKHK
VTQTMKGLDIQKVAGTWYSL
VVVPPFIQPEV
VYPFPGPIHN
VYPFPGPIHNS
VYPFPGPIHNSL
VYPFPGPIHNSLPQ
VYPFPGPIHNSLPQNIPPLT
VYPFPGPIHNSLPQNIPPLTQT
VYPFPGPIP
VYPFPGPIPNSL
VYPFPGPIPNSLPQ
VYPFPGPIPNSLPQNIPPLTQ
VYPFPGPIPNSLPQNIPPLTQT
VYVEELKPTPEGDL
VYVEELKPTPEGDLE
VYVEELKPTPEGDLEI
VYVEELKPTPEGDLEIL
VYVEELKPTPEGDLEILLQKWEND
WMHQPHQPLPPTV
WSVARLSQKFPKAEF
YPFPGPIHNSLPQ
YPFPGPIPN
YPFPGPIPNSLPQNIPPLTQT
YSLAMAASDISLL
YSLAMAASDISLLDAQ
YSLAMAASDISLLDAQSAPLR
YSLAMAASDISLLDAQSAPLRV
YSLAMAASDISLLDAQSAPLRVYVEELKPTPEGDL

YSLAMAASDISLLDAQSAPLRVYVEELKPTPEGDLEIL
YVEELKPTPEGD
YVEELKPTPEGDL
YVEELKPTPEGDLE
YVEELKPTPEGDLEI
YVEELKPTPEGDLEIL
YVEQLKPTPEGDL

ID
AASDISLLDAQSAPL
AASDISLLDAQSAPLRV
AASDISLLDAQSAPLRVY
AASDISLLDAQSAPLRVYVEE
AASDISLLDAQSAPLRVYVEELKPTPEGD
AASDISLLDAQSAPLRVYVEELKPTPEGDL
AASDISLLDAQSAPLRVYVEELKPTPEGDLE
AASDISLLDAQSAPLRVYVEELKPTPEGDLEI
AASDISLLDAQSAPLRVYVEELKPTPEGDLEIL
AASDISLLDAQSAPLRVYVEQLKPTPEGDL
AASDISLLDAQSAPLRVYVEQLKPTPEGDLEIL
ADEKKFWG
AIPENLPPLTAD
ALEKFDK
ALEKFDKALKALPMHIR
ALEKFDKALKALPMHIRL
ALEKFDKALKALPMHIRLSFNPTQL
ALNENKVLVLDTDYK
ALNENKVLVLDTDYKKYL
ALNENKVLVLDTDYKKYLL
ALNENKVLVLDTDYKKYLLF
AMAASDISLL
AMAASDISLLDAQSAPLRV
AMAASDISLLDAQSAPLRVYVEELKPTPEGDL
AMAASDISLLDAQSAPLRVYVEELKPTPEGDLEIL
AQSAPLRVYVEELKPTPEGDL
AQSAPLRVYVEELKPTPEGDLE
AQSAPLRVYVEELKPTPEGDLEIL
ASDISLLDAQ
ASDISLLDAQSAPLRVYVEELKPTPEGDLEI
ASPEVIESPPEINT
DAFLGSFLYEYSR
DAIPENLPPLT
DAIPENLPPLTA

DAIPENLPPLTAD
DALNENKVLVLDTDY
DAQSAPLRV
DAQSAPLRVY
DAQSAPLRVYVEELKPTPEGD
DAQSAPLRVYVEELKPTPEGDL
DAQSAPLRVYVEELKPTPEGDLE
DAQSAPLRVYVEELKPTPEGDLEIL
DAQSAPLRVYVEQLKPTPEGD
DAQSAPLRVYVEQLKPTPEGDL
DDEALEKFDKALKALPMHIRL
DEALEKFDKALKALPMHIRL
DEALEKFDKALKALPMHIRLSFNPTQL
DELQDKIHPFA
DELQDKIHPFAQTQS
DELQDKIHPFAQTQSL
DKIHPFAQTQS
DKTEIPTINT
DKVGINY
DKVGINYW
DLKGYGGVS
DLKGYGGVSL
DLKGYGGVSLPE
DLKGYGGVSLPEWV
DTDYKKYLL
DTDYKKYLLF
DTHKSEIAHRFKDLGEEHFKG
DTHKSEIAHRFKDLGEEHFKGLVL
EALEKFDKALKALPM
EALEKFDKALKALPMHI
EALEKFDKALKALPMHIR
EALEKFDKALKALPMHIRL
EALEKFDKALKALPMHIRLSFNPTQL
EAMAPKHKEMPFPK
EAQPTDASAQF
EASPEVIESPPEIN
EDSPEVIEGPPEINT
EDSPEVIESPPEINT
EELKPTPEGD
EELKPTPEGDL
EELKPTPEGDLE
EKFDKALKALPMHI
EKFDKALKALPMHIRL
EKTKIPAVF
ELEELNVPGE
ELEELNVPGEIVE

ELKDLKGYGGVSL
ELKPTPEGD
ELKPTPEGDL
ELKPTPEGDLE
ELKPTPEGDLEI
ELKPTPEGDLEIL
ELQDKIHPF
ELQDKIHPFAQTQSL
EMPFPKYPVE
ENSAEPEQSL
ENSAEPEQSLA
ENSAEPEQSLV
EQIVIR
ESPPEINT
EVDDEALEK
EVIESPPEINT
FDKALKALPMHI
FFVAPFPEVF
FPPQSVL
GGVSLPEW
GGVSLPEWV
GLDIQK
GLDIQKVAGTWY
GVSKVKEAMAPK
GVSKVKEAMAPKH
GVSKVKEAMAPKHK
GVSKVKEAMAPKHKEMPFPKYPVEPFTESQS
GVSKVKEAMAPKHKEMPFPKYPVEPFTESQSL
GVSKVKEAMAPKHKEMPFPKYPVQPFTESQS
HIRLSFNPTQL
HKEMPFPKYPVEPFTESQS
HLPLPLL
HLVDEPQNL
HQGLPQPFPEV
HQPHQPLPPTVM
IASGEPTSTPITE
IASGEPTSTPTIE
IASGEPTSTPTIEA
IASGEPTSTPTTE
IASGEPTSTPTTEA
ICNISCDKFLDDDLTDDIMCVKKILDKVGINY
IDALNENK
IDALNENKV
IDALNENKVL
IDALNENKVLVLDTDYK
IDALNENKVLVLDTDYKK

IESPPEINT
IHPFAQTQS
IIAEKTKIPA
IIAEKTKIPAVFKID
IIVTQTM
IIVTQTMKGLDIQKVA
IIVTQTMKGLDIQKVAGT
IIVTQTMKGLDIQKVAGTW
IIVTQTMKGLDIQKVAGTWYSL
IIVTQTMKGLDIQKVAGTWYSLAM
IIVTQTMKGLDIQKVAGTWYSLAMAASD
IIVTQTMKGLDIQKVAGTWYSLAMAASDISLLD
IIVTQTMKGLDIQKVAGTWYSLAMAASDISLLDAQSAPLR
ILNKPEDET
ILNKPEDETH
ILNKPEDETHL
IMCVKKILDKVGINY
IQKVAGTW
IQKVAGTWYSLAM
ISLLDAQSAPLRV
ISLLDAQSAPLRVY
ISLLDAQSAPLRVYVEELKPTPEGD
ISLLDAQSAPLRVYVEELKPTPEGDL
ISLLDAQSAPLRVYVEELKPTPEGDLE
ISLLDAQSAPLRVYVEELKPTPEGDLEIL
IVQNNDSTEY
IVQNNDSTEYGL
IVRYTRKVPQVSTPTL
IVTQTMKGLD
IVTQTMKGLDIQKVAGTW
KADEKKFWG
KALKALPMHIRL
KALPMHIRL
KDLGEEHF
KFDKALKALPMHI
KFDKALKALPMHIRL
KGLDIQKVAGTW
KIDALNE
KIDALNEN
KIDALNENK
KIDALNENKVLVL
KIDALNENKVLVLD
KIDALNENKVLVLDTD
KIDALNENKVLVLDTDYKK
KIDALNENKVLVLDTDYKKYL
KIDALNENKVLVLDTDYKKYLL

KIDALNENKVLVLDTDYKKYLLF
KIDALNENKVLVLDTDYKKYLLFCMENSAEPEQSLVCQCL
KIEKFQSEEQQQTEDELQDKIHPFAQTQSL
KIHPFAQT
KIHPFAQTQSL
KNQDKTEIPTINT
KPTPEGDLE
KPTPEGDLEI
KPTPEGDLEIL
KPTPEGDLEILL
KTEIPTINT
KVPQVSTPT
LDAQSAPLR
LDAQSAPLRV
LDAQSAPLRVYVEE
LDAQSAPLRVYVEELKPTPEGDL
LDAQSAPLRVYVEELKPTPEGDLE
LDAQSAPLRVYVEELKPTPEGDLEIL
LDAQSAPLRVYVEQLKPTPEGDLEIL
LDIQKVAGTW
LEDSPEVIESPPEINT
LEKFDKA
LEKFDKAL
LEKFDKALKALPMHI
LEKFDKALKALPMHIRL
LIVTQTMK
LIVTQTMKGL
LIVTQTMKGLD
LIVTQTMKGLDIQKVAG
LIVTQTMKGLDIQKVAGTWY
LIVTQTMKGLDIQKVAGTWYSL
LIVTQTMKGLDIQKVAGTWYSLA
LIVTQTMKGLDIQKVAGTWYSLAM
LIVTQTMKGLDIQKVAGTWYSLAMA
LIVTQTMKGLDIQKVAGTWYSLAMAASD
LIVTQTMKGLDIQKVAGTWYSLAMAASDISL
LIVTQTMKGLDIQKVAGTWYSLAMAASDISLL
LKALPMHIRL
LKDLKGYGGVSL
LKPTPEGDL
LKPTPEGDLE
LKPTPEGDLEIL
LNENKVLVLDTDYKKYL
LNKPEDETHL
LPMHIRL
LPQNIPPLTQT

LQDKIHPFAQ
LQDKIHPFAQT
LQDKIHPFAQTQ
LQDKIHPFAQTQSL
LRLKKYKVPQL
LSFNPTQL
LSFNPTQLEEQCH
LSFNPTQLEEQCHI
LVLDTDYK
LVLDTDYKKYLL
LVYPFPGPIHN
LVYPFPGPIHNSLPQNIPPLTQT
LVYPFPGPIPN
LVYPFPGPIPNSL
LVYPFPGPIPNSLPQNIPPLTQT
LYQEPVLGPVRGPFPIIV
LYYANKYNGV
MAASDISLL
MAASDISLLDAQSAPLRV
MAASDISLLDAQSAPLRVYVE
MAASDISLLDAQSAPLRVYVEELKPTPEGDL
MAASDISLLDAQSAPLRVYVEELKPTPEGDLE
MAASDISLLDAQSAPLRVYVEELKPTPEGDLEIL
MAIPPK
MAIPPKKNQDKTEIPT
MAIPPKKNQDKTEIPTINT
MELGHKIM
MELGHKIMRNLENTVKE
MELGHKIMRNLENTVKETIKY
MELGHKIMRNLENTVKETIKYLKS
MELGHKIMRNLENTVKETIKYLKSL
MENSAEPEQSL
MENSAEPEQSLA
MENSAEPEQSLV
MHQPHQPLPPTVM
NENKVLVLD
NIPPLTQT
NKPEDETHL
NLENTVK
NLENTVKET
NQDKTEIPTINT
NSAEPEQSL
NSAEPEQSLA
PEVIESPPEINT
PFPGPIHN
PFPGPIPN

PPKKNQDKTEIPTINT
PPKKNQDKTEIPTINTIA
PQNIPPLTQT
PQNIPPLTQTPVVVPPFIQPEV
PTPEGDLEIL
PVLGPVRGPFPIIV
PVVVPPF
PVVVPPFIQPEVMG
PVVVPPFIQPEVMGV
PVVVPPFIQPEVMGVSK
PVVVPPFL
PVVVPPFLQ
PVVVPPFLQP
PVVVPPFLQPE
PVVVPPFLQPEV
PVVVPPFLQPEVM
PVVVPPFLQPEVMG
PVVVPPFLQPEVMGV
PVVVPPFLQPEVMGVS
PVVVPPFLQPEVMGVSKVKE
PVVVPPFLQPEVMGVSKVKEAMAPK
QDKIHPF
QDKIHPFAQTQS
QDKIHPFAQTQSL
QDKTEIPTINT
QEPVLGPVRGPFPILV
QEQNQEQPIR
QLKPTPEGDL
QSLVYPFPGPIHNSLPQNIPPLTQT
QSLVYPFPGPIPNSLPQNIPPLTQT
QSWMHQPHQPLPPT
QTMKGLDIQKVAGTW
RELEELNVPG
RELEELNVPGE
RELEELNVPGEI
RELEELNVPGEIVE
RELKDLKGYGGVSL
RELKDLKGYGGVSLPEW
RLSFNPTQL
RPKHPIKHQGLPQEVL
RPKHPIKHQGLPQPFPEV
RTPEVDDEALEKFDKA
RVYVEELKPTPEGDLEILL
SAEPEQSL
SAEPEQSLA
SAEPEQSLV

SDIPNPIGSENSEK
SDISLLDAQ
SDISLLDAQSAPL
SDISLLDAQSAPLRVY
SDISLLDAQSAPLRVYVEELKPTPEGD
SDISLLDAQSAPLRVYVEELKPTPEGDLE
SEEQQQTEDELQDK
SFNPTQL
SFNPTQLEEQCHI
SGEPTSTPTIE
SGEPTSTPTIEA
SLAMAASDISL
SLAMAASDISLL
SLAMAASDISLLD
SLAMAASDISLLDAQ
SLPQNIPPLTQT
SLSQSKVLPVPQKAVPYPQ
SLSQSKVLPVPQKAVPYPQRDMPIQA
SLVYPFPGPIHNSLPQNIPPLTQT
SPEVIEGPPEINT
SPEVIESPPEINT
SPPEINT
SQKFPKAEF
SSRQPQSQNPKLPLSI
SSRQPQSQNPKLPLSIL
TDVENLHLPLP
TDVENLHLPLPL
TDVENLHLPLPLL
TDVENLHLPPL
TDYKKYLL
TEIPTIN
TEIPTINT
TFHTSGYDTQA
TIASGEPTSTPTIE
TIASGEPTSTPTTE
TIKYLKSLFSHA
TKIPAV
TLEDSPEVIESPPEINT
TPEVDDEAL
TPEVDDEALE
TPEVDDEALEK
TPEVDDEALEKF
TPEVDDEALEKFDKAL
TPVVVPPFIQPEVM
TQTMKGLDIQKVAGTW
TVKETIKYLKSL

VDDEALEKFDK
VEELKPTPE
VEELKPTPEG
VEELKPTPEGD
VEELKPTPEGDL
VEELKPTPEGDLE
VEELKPTPEGDLEI
VEELKPTPEGDLEIL
VEELKPTPEGDLEILL
VEELKPTPEGDLEILLQ
VEELKPTPEGDLEILLQK
VELLKHKPKATEEQLKT
VENLHLPLPL
VENLHLPLPLL
VKKILDKVGIN
VLDTDYK
VLDTDYKK
VLDTDYKKYL
VLDTDYKKYLL
VLDTDYKKYLLF
VLPVPQK
VLVLDTDYK
VPPFLQPEV
VPPFLQPEVM
VQVTSTAV
VRTPEVDD
VRTPEVDDE
VRTPEVDDEA
VRTPEVDDEAL
VRTPEVDDEALEKFD
VRTPEVDDEALEKFDK
VRTPEVDDEALEKFDKA
VRTPEVDDEALEKFDKAL
VRTPEVDDEALEKFDKALK
VRTPEVDDEALEKFDKALKALP
VRTPEVDDEALEKFDKALKALPM
VRTPEVDDEALEKFDKALKALPMH
VRTPEVDDEALEKFDKALKALPMHIR
VRTPEVDDEALEKFDKALKALPMHIRL
VRTPEVDDEALEKFDKALKALPMHIRLSFNPTQ
VRTPEVDDEALEKFDKALKALPMHIRLSFNPTQL
VRTPEVDDEALEKFDKALKALPMHIRLSFNPTQLEEQCHI
VSKVKEAMAPK
VSKVKEAMAPKHK
VVVPPFIQPEV
VYPFPGPIH

VYPFPGPIHN
VYPFPGPIHNS
VYPFPGPIHNSL
VYPFPGPIHNSLPQ
VYPFPGPIHNSLPQNIPPLTQT
VYPFPGPIP
VYPFPGPIPN
VYPFPGPIPNS
VYPFPGPIPNSL
VYPFPGPIPNSLPQ
VYPFPGPIPNSLPQNIPPLT
VYPFPGPIPNSLPQNIPPLTQ
VYPFPGPIPNSLPQNIPPLTQT
VYPFPGPIPNSLPQNIPPLTQTPV
VYVEELKPTPEGDL
VYVEELKPTPEGDLE
VYVEELKPTPEGDLEIL
VYVEELKPTPEGDLEILL
VYVEELKPTPEGDLEILLQ
VYVEELKPTPEGDLEILLQK
WLAHKALCSEKLDQ
WMHQPHQPLPPTVM
WSVARLSQKFPKAEF
YKKYLLF
YPFPGPIHN
YPFPGPIPN
YPFPGPIPNSLPQNIPPLTQT
YQEPVLGPVRGPFPILV
YSLAMAASDIS
YSLAMAASDISL
YSLAMAASDISLL
YSLAMAASDISLLDAQSAPLRV
YVEELKPTPEGD
YVEELKPTPEGDL
YVEELKPTPEGDLE
YVEELKPTPEGDLEI
YVEELKPTPEGDLEIL
YVEELKPTPEGDLEILL
YVEELKPTPEGDLEILLQ
YVEQLKPTPEGDL

ID.A.10min
AASDISLLDAQSAPL
AASDISLLDAQSAPLRVYVEE

AASDISLLDAQSAPLRVYVEELKPTPEGDLEIL
AASDISLLDAQSAPLRVYVEQLKPTPEGDL
AASDISLLDAQSAPLRVYVEQLKPTPEGDLEIL
ADEKKFWG
AIPENLPPLTAD
ALEKFDKAL
ALEKFDKALKALPMHIR
ALPMHI
AMAASDISLLDAQSAPLRV
AQALIVTQTMK
AQSAPLRVYVEELKPTPEGDLE
ASDISLLDAQ
ASDISLLDAQSAPLR
ASDISLLDAQSAPLRVYVEELKPTPEGDLEIL
ASGEPTSTPTIE
ASPEVIESPPEINT
DAFLGSFLYEYSR
DAIPENLPPLT
DAIPENLPPLTA
DAIPENLPPLTAD
DALNENKVLVLDTDY
DAQSAPLRVY
DAQSAPLRVYVE
DAQSAPLRVYVEELKPTPEGD
DAQSAPLRVYVEELKPTPEGDLE
DAQSAPLRVYVEELKPTPEGDLEI
DAQSAPLRVYVEQLKPTPEGD
DDEALEKFDKA
DDEALEKFDKALKALPMHIRL
DEALEKFDK
DEALEKFDKALKALPM
DELQDKIHP
DELQDKIHPF
DELQDKIHPFA
DELQDKIHPFAQT
DELQDKIHPFAQTQS
DELQDKIHPFAQTQSL
DKALKALPMHI
DKIHPFAQT
DKIHPFAQTQS
DKIHPFAQTQSL
DKTEIPTINT
DKVGINY
DLKGYGGVS
DLKGYGGVSL

DLKGYGGVSLPE
DLKGYGGVSLPEWV
DLSKEPSISREDL
DSPEVIEGPPEINT
DSPEVIESPPEINT
DTHKSEIAHRFKDLGEEHFKG
EALEKFDKALKALPM
EALEKFDKALKALPMHI
EALEKFDKALKALPMHIR
EAQPTDASAQF
EASPEVIESPPEIN
EDSPEVIEGPPEINT
EDSPEVIESPPEINT
EELKPTPEGD
EELKPTPEGDL
EELKPTPEGDLE
EELKPTPEGDLEI
EELKPTPEGDLEIL
EKTKIPAVF
ELEELNVPGE
ELEELNVPGEIVE
ELGHKIM
ELKDLKGYGGVSL
ELKPTPEGD
ELKPTPEGDL
ELKPTPEGDLE
ELKPTPEGDLEI
ELKPTPEGDLEIL
EMPFPKYPVE
EMPFPKYPVEPFTESQ
ENSAEPEQSL
ENSAEPEQSLA
ENSAEPEQSLV
EQIVIR
ESPPEINT
EVDDEALEK
EVDDEALEKFDKA
EVIESPPEINT
FAQTQSLVYPFPGP
FHTSGYDTQA
FPPQSVL
FVAPFPEV
FVAPFPEVFGK
FVAPFPEVFGKE
FVAPFPEVFGKEKVNE
FVAPFPEVFGKEKVNEL

GEPTSTPTIE
GGVSLPEW
GGVSLPEWV
GLDIQK
GLDIQKVA
GLDIQKVAGT
GLDIQKVAGTWY
GLDIQKVAGTWYSLAMA
GVSKVKEAMAPKHKEMPFPKYPVEPFTESQS
GVSKVKEAMAPKHKEMPFPKYPVEPFTESQSL
GVSKVKEAMAPKHKEMPFPKYPVQPFTESQS
GYGGVSLPEWV
HIRLSFNPTQ
HKEMPFPKYPVEPF
HLPLPLL
HLVDEPQN
HLVDEPQNL
HQGLPQPFPEV
HQPHQPLPPT
HQPHQPLPPTVM
IASGEPTSTPITE
IASGEPTSTPTIE
IASGEPTSTPTIEA
IASGEPTSTPTTE
IASGEPTSTPTTEA
IDALNENK
IDALNENKV
IDALNENKVLVLDTDYKK
IESPPEINT
IHPFAQTQS
IIVTQTM
IIVTQTMKGLDIQKVA
IIVTQTMKGLDIQKVAGTWYSLAMAA
IIVTQTMKGLDIQKVAGTWYSLAMAASDISLLD
IIVTQTMKGLDIQKVAGTWYSLAMAASDISLLDAQSAPLR
ILNKPEDET
ILNKPEDETH
ILNKPEDETHL
IPAVFKIDALNENKVLVLDTDYK
ISLLDAQSAPLRVYVEELKPTPEGDLE
IVQNNDSTEY
IVQNNDSTEYG
IVQNNDSTEYGL
IVTQTMKG
IVTQTMKGLD
IVTQTMKGLDIQK

IVTQTMKGLDIQKVAGT
IVTQTMKGLDIQKVAGTW
KADEKKFWG
KDLGEEHF
KFDKALKALPMHI
KGLDIQK
KGLDIQKVAGTW
KIDALNE
KIDALNEN
KIDALNENK
KIDALNENKVLVLD
KIDALNENKVLVLDTDYKK
KIEKFQSEEQQQTEDELQDKIHPFAQTQSL
KIHPFAQTQ
KIHPFAQTQS
KIHPFAQTQSL
KILDKVGIN
KNQDKTEIPTINT
KPTPEGDLE
KPTPEGDLEI
KPTPEGDLEIL
KPTPEGDLEILL
KTEIPTINT
KVPQVSTPT
LDAQSAPLRVYVEE
LDAQSAPLRVYVEELKPTPEGDL
LDIQKVAGTW
LEDSPEVIESPPEINT
LEKFDKALKALPMHI
LIVTQTMKGL
LIVTQTMKGLDIQKVAGTWYSLAM
LIVTQTMKGLDIQKVAGTWYSLAMAASD
LIVTQTMKGLDIQKVAGTWYSLAMAASDISLL
LKPTPEGDL
LKPTPEGDLE
LKPTPEGDLEIL
LNENKVLVLDTDYKKYL
LNKPEDETHL
LPQNIPPLTQT
LQDKIHPFAQ
LQDKIHPFAQTQSL
LSFNPTQL
LSFNPTQLEEQ
LSFNPTQLEEQCH
LSFNPTQLEEQCHI
LVLDTDYK

LVYPFPGPIHN
LVYPFPGPIHNSLPQNIPPLTQT
LVYPFPGPIPN
LVYPFPGPIPNS
LVYPFPGPIPNSLPQNIPPLTQT
LYYANKYNGV
MAASDISLL
MAASDISLLDAQSAPLRV
MAASDISLLDAQSAPLRVYVEELKPTPEGDLEIL
MAIPPK
MAIPPKKNQDKTEIPTINT
MELGHKIMRNLENTVKE
MELGHKIMRNLENTVKETIK
MELGHKIMRNLENTVKETIKYLKS
MELGHKIMRNLENTVKETIKYLKSL
MENSAEPEQSL
MENSAEPEQSLA
MENSAEPEQSLV
MGVSKVKEAMAPK
MHQPHQPLPPT
MHQPHQPLPPTVM
NIPPLTQT
NKPEDETHL
NKVLVLDTDY
NKVLVLDTDYKK
NKVLVLDTDYKKYL
NLENTVK
NLENTVKET
NQDKTEIPTINT
NSAEPEQSL
NSAEPEQSLA
PEVIESPPEINT
PFPGPIHN
PFPGPIPN
PPKKNQDKTEIPTINTIA
PPLTQTPVVVPPFLQPEVMGVSKVKEAMAPKHKEMPF
PPLTQTPVVVPPFLQPEVMGVSKVKEAMAPKHKEMPFP
PQNIPPLTQT
PQNIPPLTQTPVVVPPFIQPEV
PTPEGDLEIL
PVVVPPF
PVVVPPFIQPEVMG
PVVVPPFIQPEVMGV
PVVVPPFL
PVVVPPFLQ
PVVVPPFLQP

PVVVPPFLQPE
PVVVPPFLQPEV
PVVVPPFLQPEVM
PVVVPPFLQPEVMG
PVVVPPFLQPEVMGV
PVVVPPFLQPEVMGVS
QDKIHPF
QDKIHPFAQTQS
QDKIHPFAQTQSL
QDKTEIPTINT
QEPVLGPVRGPFPILV
QEQNQEQPIR
QLKPTPEGDL
QSLVYPFPGPIHNSLPQNIPPLTQT
QSLVYPFPGPIPNSLPQNIPPLTQT
QSWMHQPHQPLPPT
QSWMHQPHQPLPPTV
QSWMHQPHQPLPPTVM
QTMKGLDIQKVAGTW
RELEELNVPG
RELEELNVPGE
RELEELNVPGEIVE
RELKDLKGYGGVSLPEW
RHPYFYAPEL
RLSFNPTQ
RPKHPIKHQGLPQPFPEV
RTPEVDDEALEKFDK
RTPEVDDEALEKFDKA
SAEPEQSL
SAEPEQSLA
SAEPEQSLV
SDIPNPIGSENSEK
SDIPNPIGSENSEKT
SDISLLDAQ
SDISLLDAQSAPL
SDISLLDAQSAPLRV
SDISLLDAQSAPLRVYVEELKPTPEGDLEIL
SEEQQQTEDELQDK
SFNPTQL
SFNPTQLEEQ
SFNPTQLEEQCHI
SGEPTSTPTIE
SGEPTSTPTIEA
SKEPSISREDL
SLAMAASDISLL
SLAMAASDISLLDAQSAPLRVY

SLPQNIPPLTQT
SLSQSKVLPVPQKAVPYPQ
SLVYPFPGPIHNSLPQNIPPLTQT
SLVYPFPGPIPNSLPQNIPPLTQT
SPEVIEGPPEINT
SPEVIESPPEINT
SPPEINT
SQKFPKAEF
SSRQPQSQNPKLPLSI
TEIPTIN
TEIPTINT
TIASGEPTSTPTIE
TIASGEPTSTPTTE
TIKYLKSLFSHA
TKIPAV
TLEDSPEVIESPPEINT
TPEGDLEIL
TPEVDDEAL
TPEVDDEALE
TPEVDDEALEK
TPEVDDEALEKF
TPEVDDEALEKFDKAL
TPVVVPPFIQPEVM
TQTMKGLDIQKVAGTW
VEELKPTPE
VEELKPTPEG
VEELKPTPEGD
VEELKPTPEGDL
VEELKPTPEGDLE
VEELKPTPEGDLEI
VEELKPTPEGDLEIL
VEELKPTPEGDLEILL
VEELKPTPEGDLEILLQ
VEELKPTPEGDLEILLQK
VLDTDYK
VLDTDYKK
VLDTDYKKYL
VLDTDYKKYLL
VLDTDYKKYLLF
VLPVPQK
VLVLDTDYK
VLVLDTDYKK
VLVLDTDYKKYL
VPPFLQPEV
VPPFLQPEVM
VQVTSTAV

VRTPEVDD
VRTPEVDDE
VRTPEVDDEA
VRTPEVDDEAL
VRTPEVDDEALEKF
VRTPEVDDEALEKFD
VRTPEVDDEALEKFDK
VRTPEVDDEALEKFDKA
VRTPEVDDEALEKFDKALKA
VRTPEVDDEALEKFDKALKALPM
VRTPEVDDEALEKFDKALKALPMHI
VRTPEVDDEALEKFDKALKALPMHIRL
VSKVKEAMAPKHK
VTQTMKGLD
VTQTMKGLDIQKVAGTW
VTQTMKGLDIQKVAGTWYSL
VVVPPFIQPEV
VYPFPGPIH
VYPFPGPIHN
VYPFPGPIHNS
VYPFPGPIHNSL
VYPFPGPIHNSLPQ
VYPFPGPIHNSLPQNIPPLT
VYPFPGPIHNSLPQNIPPLTQTPVVVPPF
VYPFPGPIP
VYPFPGPIPN
VYPFPGPIPNS
VYPFPGPIPNSL
VYPFPGPIPNSLPQ
VYPFPGPIPNSLPQNIPPLT
VYPFPGPIPNSLPQNIPPLTQ
VYVEELKPTPEGDL
VYVEELKPTPEGDLE
VYVEELKPTPEGDLEI
VYVEELKPTPEGDLEIL
VYVEELKPTPEGDLEILL
VYVEELKPTPEGDLEILLQ
WMHQPHQPLPPTVM
WSVARLSQKFPKAEF
YKKYLLF
YPFPGPIHN
YPFPGPIHNSLPQNIPPLTQT
YPFPGPIPN
YPFPGPIPNSLPQNIPPLTQT
YSLAMAASDI
YSLAMAASDIS

YSLAMAASDISLLDAQSAPLRVYVEELKPTPEGDLEIL
YVEELKPTPE
YVEELKPTPEG
YVEELKPTPEGD
YVEELKPTPEGDL
YVEELKPTPEGDLE
YVEELKPTPEGDLEI
YVEELKPTPEGDLEIL
YVEELKPTPEGDLEILL
YVEQLKPTPEGDL
YVPKAFDEKL

ID.A.60min
AASDISLLDAQ
AASDISLLDAQSAPL
AASDISLLDAQSAPLRV
AASDISLLDAQSAPLRVYVEELKPTPEGDLEIL
AASDISLLDAQSAPLRVYVEQLKPTPEGDLEIL
ADEKKFWG
AIPENLPPLTAD
ALPMHI
AMAASDISLL
AQALIVTQTMK
ASDISLLDAQSAPLR
ASDISLLDAQSAPLRVYVEELKPTPEGDLEIL
ASGEPTSTPTIE
ASGEPTSTPTIEA
ASPEVIESPPEINT
DAIPENLPPLT
DAIPENLPPLTA
DAIPENLPPLTAD
DALNENKVLV
DALNENKVLVLDTDY
DAQSAPLRVY
DAQSAPLRVYVE
DAQSAPLRVYVEELKPTPEGDLE
DAQSAPLRVYVEELKPTPEGDLEI
DDEALEKFDKA
DEALEKFDK
DEALEKFDKALKALPM
DEALEKFDKALKALPMHIRL
DELQDKIHPF
DELQDKIHPFA

DELQDKIHPFAQTQS
DELQDKIHPFAQTQSL
DKALKALPMHI
DKIHPFAQT
DKIHPFAQTQS
DKIHPFAQTQSL
DKTEIPTINT
DKVGINYW
DLKGYGGVS
DLKGYGGVSL
DLKGYGGVSLPE
DLSKEPSISREDL
DSPEVIEGPPEINT
DSPEVIESPPEINT
EALEKFDKALKALPM
EALEKFDKALKALPMHI
EAQPTDASAQF
EASPEVIESPPEIN
EDSPEVIEGPPEINT
EDSPEVIESPPEINT
EELKPTPEGD
EELKPTPEGDL
EELKPTPEGDLE
EELKPTPEGDLEI
EELKPTPEGDLEIL
EKTKIPAVF
ELEELNVPGEIVE
ELKPTPEGD
ELKPTPEGDL
ELKPTPEGDLE
ELKPTPEGDLEI
ELKPTPEGDLEIL
EMPFPKYPVE
EMPFPKYPVEPFTESQ
ENSAEPEQSL
ENSAEPEQSLA
ENSAEPEQSLV
ESPPEINT
EVDDEALEK
EVDDEALEKFDKA
EVIESPPEINT
FAQTQSLVYPFPGP
FKDLGEEHFKGLV
FVAPFPEVFGKE
FVAPFPEVFGKEKVNEL
GEPTSTPTIE

GGVSLPEW
GGVSLPEWV
GLDIQK
GLDIQKVA
GLDIQKVAGT
GLDIQKVAGTWY
GVSKVKEAMAPKHKEMPFPKYPVEPFTESQS
GVSKVKEAMAPKHKEMPFPKYPVEPFTESQSL
GYGGVSLPEWV
HIRLSFNPTQ
HKEMPFPKYPVEPF
HLPLPLL
HLVDEPQN
HLVDEPQNL
HQGLPQPFPEV
HQPHQPLPPT
HQPHQPLPPTVM
IASGEPTSTPITE
IASGEPTSTPTIE
IASGEPTSTPTIEA
IASGEPTSTPTTE
IASGEPTSTPTTEA
IDALNENK
IDALNENKV
IDALNENKVLVLDTDYK
IDALNENKVLVLDTDYKK
IESPPEINT
IHPFAQTQSL
IIAEKTKIPAV
IIVTQTM
IIVTQTMKGLDIQKVA
IIVTQTMKGLDIQKVAGTWYSLAMAA
IIVTQTMKGLDIQKVAGTWYSLAMAASDISLLD
IIVTQTMKGLDIQKVAGTWYSLAMAASDISLLDAQSAPLR
ILNKPEDET
ILNKPEDETH
ILNKPEDETHL
IPAVFKIDALNENKVLVLDTDYK
IVQNNDSTEY
IVQNNDSTEYGL
IVTQTMKG
IVTQTMKGLD
IVTQTMKGLDIQK
IVTQTMKGLDIQKVAGT
KADEKKFWG
KFDKALKALPM

KFDKALKALPMHI
KGLDIQK
KGLDIQKVAGTW
KIDALNE
KIDALNEN
KIDALNENKVLVLDTDYK
KIDALNENKVLVLDTDYKK
KIEKFQSEEQQQTEDELQDKIHPFAQTQSL
KIHPFAQTQ
KIHPFAQTQS
KIHPFAQTQSL
KILDKVGIN
KNQDKTEIPT
KNQDKTEIPTINT
KPTPEGDLE
KPTPEGDLEI
KPTPEGDLEIL
KPTPEGDLEILL
KTEIPTINT
KVPQVSTPT
LDAQSAPLRVYVEE
LDAQSAPLRVYVEELKPTPEGDL
LDAQSAPLRVYVEELKPTPEGDLEIL
LDAQSAPLRVYVEQLKPTPEGDLEIL
LEDSPEVIESPPEINT
LIVTQTMKGL
LIVTQTMKGLDI
LIVTQTMKGLDIQKVAGTWYSLAM
LIVTQTMKGLDIQKVAGTWYSLAMAASDISLL
LKDLKGYGGVSL
LKPTPEGDL
LKPTPEGDLE
LKPTPEGDLEIL
LNENKVLV
LNENKVLVLDTDYKKYL
LNKPEDETHL
LPQNIPPLTQT
LQDKIHPF
LQDKIHPFAQ
LQDKIHPFAQTQSL
LSFNPTQL
LSFNPTQLEEQ
LVLDTDYK
LVYPFPGPIHN
LVYPFPGPIHNSLPQNIPPLTQT
LVYPFPGPIPN

LVYPFPGPIPNS
LVYPFPGPIPNSLPQNIPPLTQT
LYYANKYNGV
LYYANKYNGVF
MAASDISLL
MAASDISLLDAQ
MAASDISLLDAQSAPLRV
MAASDISLLDAQSAPLRVYVEELKPTPEGDLEIL
MAIPPK
MAIPPKKNQDKTEIPTINT
MELGHKIMRNLENTVKE
MELGHKIMRNLENTVKETIK
MELGHKIMRNLENTVKETIKYLKS
MELGHKIMRNLENTVKETIKYLKSL
MENSAEPEQSL
MENSAEPEQSLA
MENSAEPEQSLV
MGVSKVKEAMAPK
MHQPHQPLPPT
MHQPHQPLPPTVM
NIPPLTQT
NKPEDETHL
NKVLVLDTDYKK
NLENTVK
NLENTVKET
NQDKTEIPTINT
NSAEPEQSL
NSAEPEQSLA
PEVIESPPEINT
PFPGPIHN
PFPGPIPN
PPKKNQDKTEIPTINTI
PPKKNQDKTEIPTINTIA
PPLTQTPVVVPPFLQPEVMGVSKVKEAMAPKHKEMPFP
PQNIPPLTQT
PTPEGDLEIL
PVVVPPF
PVVVPPFIQPEVMG
PVVVPPFL
PVVVPPFLQ
PVVVPPFLQP
PVVVPPFLQPE
PVVVPPFLQPEV
PVVVPPFLQPEVM
PVVVPPFLQPEVMG
PVVVPPFLQPEVMGV

PVVVPPFLQPEVMGVSKVKEAMAPK
QDKIHPF
QDKIHPFAQTQ
QDKIHPFAQTQS
QDKIHPFAQTQSL
QDKTEIPTINT
QEQNQEQPIR
QLKPTPEGDL
QPQSQNPKLPLS
QSWMHQPHQPLPPT
QSWMHQPHQPLPPTVM
QTMKGLDIQKVAGTW
RELEELNVPGE
RHPYFYAPEL
RLSFNPTQ
RTPEVDDEALEKFDK
RTPEVDDEALEKFDKA
SAEPEQSL
SAEPEQSLA
SAEPEQSLV
SDIPNPIGSENSEK
SDIPNPIGSENSEKT
SDISLLDAQ
SDISLLDAQSAPL
SDISLLDAQSAPLRV
SEEQQQTEDELQDK
SFNPTQL
SFNPTQLEEQ
SFNPTQLEEQCHI
SGEPTSTPTIE
SGEPTSTPTIEA
SKEPSISREDL
SLAMAASDISLL
SLAMAASDISLLDAQSAPLRVY
SLPQNIPPLTQT
SLVYPFPGPIPNSLPQNIPPLTQT
SPEVIEGPPEINT
SPEVIESPPEINT
SPPEINT
SQKFPKAEF
TDVENLHLPLPL
TEIPTIN
TEIPTINT
TIASGEPTSTPTIE
TIASGEPTSTPTTE
TLEDSPEVIESPPEINT

TPEGDLEIL
TPEVDDEAL
TPEVDDEALE
TPEVDDEALEK
TPEVDDEALEKF
TPEVDDEALEKFDKAL
TPVVVPPFIQPEVM
TQTMKGLDIQKVAGTW
VEELKPTPE
VEELKPTPEG
VEELKPTPEGD
VEELKPTPEGDL
VEELKPTPEGDLE
VEELKPTPEGDLEI
VEELKPTPEGDLEIL
VEELKPTPEGDLEILL
VEELKPTPEGDLEILLQ
VLDTDYK
VLDTDYKK
VLDTDYKKYL
VLDTDYKKYLL
VLDTDYKKYLLF
VLPVPQK
VLVLDTDYK
VLVLDTDYKK
VLVLDTDYKKYL
VPPFLQPEV
VPPFLQPEVM
VRTPEVDDE
VRTPEVDDEA
VRTPEVDDEAL
VRTPEVDDEALE
VRTPEVDDEALEKF
VRTPEVDDEALEKFD
VRTPEVDDEALEKFDKA
VRTPEVDDEALEKFDKAL
VRTPEVDDEALEKFDKALK
VRTPEVDDEALEKFDKALKA
VRTPEVDDEALEKFDKALKALPM
VSKVKEAMAPKHK
VTQTMKGLD
VVVPPFIQPEV
VYPFPGPIH
VYPFPGPIHN
VYPFPGPIHNS
VYPFPGPIHNSL

VYPFPGPIHNSLPQNIPPLTQTPVVVPPF
VYPFPGPIP
VYPFPGPIPN
VYPFPGPIPNS
VYPFPGPIPNSL
VYPFPGPIPNSLPQ
VYPFPGPIPNSLPQNIPPLT
VYPFPGPIPNSLPQNIPPLTQ
VYPFPGPIPNSLPQNIPPLTQTPV
VYVEELKPTPEGDL
VYVEELKPTPEGDLE
VYVEELKPTPEGDLEI
VYVEELKPTPEGDLEIL
VYVEELKPTPEGDLEILL
WMHQPHQPLPPTVM
WSVARLSQKFPKAEF
YPFPGPIHN
YPFPGPIPN
YPFPGPIPNSLPQNIPPLTQT
YSLAMAASDIS
YSLAMAASDISLLDAQSAPL
YSLAMAASDISLLDAQSAPLRVYVEELKPTPEGDL
YVEELKPTPE
YVEELKPTPEG
YVEELKPTPEGD
YVEELKPTPEGDL
YVEELKPTPEGDLE
YVEELKPTPEGDLEI
YVEELKPTPEGDLEIL
YVEELKPTPEGDLEILL
YVEQLKPTPEGDL

ID.A.120min
AASDISLLDAQ
AASDISLLDAQSAPLRV
AASDISLLDAQSAPLRVYVEELKPTPEGD
AASDISLLDAQSAPLRVYVEELKPTPEGDLEIL
AASDISLLDAQSAPLRVYVEQLKPTPEGDLEIL
ADEKKFWG
AIPENLPPLTAD
ALEKFDKALKALPMHI
ALPMHI
AMAASDISLL
AMAASDISLLDAQSAPLRV

AMAASDISLLDAQSAPLRVYVEELKPTPEGDLEIL
AQALIVTQTMK
AQSAPLRVYVEELKPTPEGDLEIL
ASDISLLDAQSAPLRV
ASDISLLDAQSAPLRVYVEELKPTPEGDLEIL
ASGEPTSTPTIE
ASGEPTSTPTIEA
ASPEVIESPPEINT
DAIPENLPPLT
DAIPENLPPLTA
DAIPENLPPLTAD
DALNENKVLV
DALNENKVLVLDTDY
DAQSAPLRV
DAQSAPLRVY
DAQSAPLRVYVE
DAQSAPLRVYVEELKPTPEGDLE
DAQSAPLRVYVEELKPTPEGDLEIL
DDEALEKFDKA
DEALEKFDK
DELQDKIHPF
DELQDKIHPFAQTQS
DELQDKIHPFAQTQSL
DKALKALPMHI
DKIHPFAQT
DKIHPFAQTQSL
DKTEIPTINT
DLKGYGGVSL
DLSKEPSISREDL
DSPEVIEGPPEINT
DSPEVIESPPEINT
DTHKSEIAHRFKDLGEEHFKG
EALEKFDKALKALPM
EALEKFDKALKALPMHI
EAQPTDASAQF
EASPEVIESPPEIN
EDSPEVIEGPPEINT
EDSPEVIESPPEINT
EELKPTPEGD
EELKPTPEGDL
EELKPTPEGDLE
EELKPTPEGDLEI
EELKPTPEGDLEIL
EKFDKALKALPMHI
EKFDKALKALPMHIR
EKFDKALKALPMHIRL

EKTKIPAVF
ELEELNVPGEIVE
ELKPTPEGD
ELKPTPEGDL
ELKPTPEGDLE
ELKPTPEGDLEI
ELKPTPEGDLEIL
ENSAEPEQSL
ENSAEPEQSLA
ENSAEPEQSLV
ESPPEINT
EVDDEALEK
EVDDEALEKFDKA
EVIESPPEINT
FAQTQSLVYPFPGP
FKDLGEEHFKGLV
FQINNKIW
FVAPFPEV
FVAPFPEVFGKEKVNE
GEPTSTPTIE
GGVSLPEW
GGVSLPEWV
GLDIQK
GLDIQKVA
GLDIQKVAGT
GLDIQKVAGTWY
GVSKVKEAMAPKHKEMPFPKYPVEPFTESQS
HIRLSFNPTQ
HKEMPFPKYPVEPF
HLVDEPQN
HLVDEPQNL
HQGLPQPFPEV
HQPHQPLPPT
HQPHQPLPPTVM
IASGEPTSTPITE
IASGEPTSTPTIE
IASGEPTSTPTIEA
IASGEPTSTPTTE
IASGEPTSTPTTEA
IDALNENK
IDALNENKVLV
IDALNENKVLVLDTDY
IDALNENKVLVLDTDYK
IESPPEINT
IHPFAQTQS
IHPFAQTQSL

IIVTQTMKGLDIQKVA
IIVTQTMKGLDIQKVAGTWYSLAMAASDISLLD
IIVTQTMKGLDIQKVAGTWYSLAMAASDISLLDAQSAPLR
ILNKPEDET
ILNKPEDETH
ILNKPEDETHL
IPAVFKIDALNENKVLVLDTDYK
ISLLDAQSAPLRVYVEELKPTPEGDLEIL
IVQNNDSTEY
IVQNNDSTEYG
IVTQTMKGLD
IVTQTMKGLDIQKVAGT
KADEKKFWG
KFDKALKALPM
KFDKALKALPMHI
KFLDDDLTDD
KGLDIQKVAGTW
KIDALNE
KIDALNEN
KIDALNENKVLVLDTD
KIDALNENKVLVLDTDYK
KIDALNENKVLVLDTDYKK
KIDALNENKVLVLDTDYKKY
KIHPFAQTQ
KIHPFAQTQS
KIHPFAQTQSL
KILDKVGIN
KNQDKTEIPTINT
KPTPEGDLE
KPTPEGDLEI
KPTPEGDLEIL
KPTPEGDLEILL
KTEIPTINT
KVPQVSTPT
LDAQSAPLRVYVEE
LDAQSAPLRVYVEELKPTPEGDL
LDAQSAPLRVYVEELKPTPEGDLE
LEDSPEVIESPPEINT
LIVTQTMKGL
LIVTQTMKGLDI
LIVTQTMKGLDIQKVAGTWYSLAM
LIVTQTMKGLDIQKVAGTWYSLAMAASDISL
LKDLKGYGGVSL
LKGYGGVSLPEW
LKPTPEGDL
LKPTPEGDLE

LKPTPEGDLEIL
LNENKVLV
LNENKVLVLDTDYKKYL
LNKPEDETHL
LPQNIPPLTQT
LQDKIHPF
LQDKIHPFAQ
LQDKIHPFAQTQSL
LSFNPTQL
LSFNPTQLEEQ
LTDVENLHLPLPLL
LVLDTDYK
LVYPFPGPIHN
LVYPFPGPIHNSLPQNIPPLTQT
LVYPFPGPIPN
LVYPFPGPIPNSLPQNIPPLTQT
LYQEPVLGPVRGPFPIIV
LYYANKYNGV
MAASDISLLDAQ
MAASDISLLDAQSAPLRV
MAIPPK
MAIPPKKNQDKTEIPTINT
MELGHKIMRNLENTVKE
MELGHKIMRNLENTVKETIK
MELGHKIMRNLENTVKETIKYLKS
MENSAEPEQSL
MENSAEPEQSLA
MENSAEPEQSLV
MGVSKVKEAMAPK
MHQPHQPLPPT
MHQPHQPLPPTVM
NIPPLTQT
NKPEDETHL
NKVLVLDTDYKK
NLENTVK
NLENTVKET
NLENTVKETIK
NQDKTEIPTINT
NSAEPEQSL
NSAEPEQSLA
PEVIESPPEINT
PFPGPIHN
PFPGPIPN
PPKKNQDKTEIPTINTI
PPKKNQDKTEIPTINTIA
PPLTQTPVVVPPFLQPEVMGVSKVKEAMAPKHKEMPF

PPLTQTPVVVPPFLQPEVMGVSKVKEAMAPKHKEMPFP
PQNIPPLTQT
PQNIPPLTQTPVVVPPFIQPEV
PTPEGDLEIL
PVVVPPF
PVVVPPFIQPEVMG
PVVVPPFL
PVVVPPFLQ
PVVVPPFLQP
PVVVPPFLQPE
PVVVPPFLQPEV
PVVVPPFLQPEVM
PVVVPPFLQPEVMG
PVVVPPFLQPEVMGVSKVKEAMAPK
QDKIHPF
QDKIHPFAQTQ
QDKIHPFAQTQS
QDKIHPFAQTQSL
QDKTEIPTINT
QLKPTPEGDL
QSWMHQPHQPLPPT
QSWMHQPHQPLPPTVM
QTMKGLDIQKVAGTW
RLSFNPTQ
RTPEVDDEALEKFDK
RTPEVDDEALEKFDKA
SAEPEQSL
SAEPEQSLA
SAEPEQSLV
SDIPNPIGSENSEK
SDISLLDAQ
SDISLLDAQSAPL
SDISLLDAQSAPLRV
SEEQQQTEDELQDK
SFNPTQL
SFNPTQLEEQ
SFNPTQLEEQCHI
SGEPTSTPTIE
SGEPTSTPTIEA
SKEPSISREDL
SLAMAASDISLL
SLAMAASDISLLDAQSAPLRVY
SLPQNIPPLTQT
SLVYPFPGPIPNSLPQNIPPLTQT
SPEVIEGPPEINT
SPEVIESPPEINT

SPPEINT
SQKFPKAEF
TDVENLHLPLPL
TEIPTIN
TEIPTINT
TIASGEPTSTPTIE
TIASGEPTSTPTTE
TLEDSPEVIESPPEINT
TPEGDLEIL
TPEVDDEAL
TPEVDDEALE
TPEVDDEALEK
TPEVDDEALEKF
TPEVDDEALEKFD
TPEVDDEALEKFDKAL
TPVVVPPFIQPEVM
TQTMKGLDIQKVAGTW
VDDEALEKFDK
VDDEALEKFDKA
VEELKPTPE
VEELKPTPEG
VEELKPTPEGD
VEELKPTPEGDL
VEELKPTPEGDLE
VEELKPTPEGDLEI
VEELKPTPEGDLEIL
VEELKPTPEGDLEILL
VLDTDYK
VLDTDYKKYL
VLDTDYKKYLL
VLDTDYKKYLLF
VLPVPQK
VLVLDTDYK
VLVLDTDYKK
VLVLDTDYKKYL
VPPFLQPEV
VPPFLQPEVM
VRTPEVDDE
VRTPEVDDEA
VRTPEVDDEAL
VRTPEVDDEALEKF
VRTPEVDDEALEKFD
VRTPEVDDEALEKFDK
VRTPEVDDEALEKFDKA
VRTPEVDDEALEKFDKALKA
VRTPEVDDEALEKFDKALKALPM

VRTPEVDDEALEKFDKALKALPMHI
VRTPEVDDEALEKFDKALKALPMHIRL
VSKVKEAMAPK
VSKVKEAMAPKHK
VTQTMKGLD
VVVPPFIQPEV
VYPFPGPIH
VYPFPGPIHN
VYPFPGPIHNS
VYPFPGPIHNSL
VYPFPGPIHNSLPQNIPPLT
VYPFPGPIP
VYPFPGPIPN
VYPFPGPIPNS
VYPFPGPIPNSLPQ
VYPFPGPIPNSLPQNIPPLTQ
VYPFPGPIPNSLPQNIPPLTQTPV
VYVEELKPTPEGDL
VYVEELKPTPEGDLE
VYVEELKPTPEGDLEI
VYVEELKPTPEGDLEIL
VYVEELKPTPEGDLEILL
WMHQPHQPLPPTV
WMHQPHQPLPPTVM
WSVARLSQKFPKAEF
YPFPGPIHN
YPFPGPIPN
YPFPGPIPNS
YPFPGPIPNSLPQNIPPLTQT
YSLAMAASDIS
YSLAMAASDISLLDAQSAPL
YSLAMAASDISLLDAQSAPLRVYVEELKPTPEGDLEIL
YVEELKPTPE
YVEELKPTPEG
YVEELKPTPEGD
YVEELKPTPEGDL
YVEELKPTPEGDLE
YVEELKPTPEGDLEI
YVEELKPTPEGDLEIL
YVEQLKPTPEGDL

ID.B.120min
AASDISLLDAQSAPL
AASDISLLDAQSAPLRV

AASDISLLDAQSAPLRVY
AASDISLLDAQSAPLRVYVE
AASDISLLDAQSAPLRVYVEELKPTPEGDL
AASDISLLDAQSAPLRVYVEELKPTPEGDLE
AASDISLLDAQSAPLRVYVEELKPTPEGDLEIL
AASDISLLDAQSAPLRVYVEQLKPTPEGDL
AIVQNNDSTEYG
ALEKFDKALKA
ALNENKVLVLDTDYK
ALNENKVLVLDTDYKKYLL
ALNENKVLVLDTDYKKYLLF
ALPMHIRLSFNPTQL
AMAASDISLLDAQSAPL
AMAASDISLLDAQSAPLRVYVEELKPTPEGDLEIL
AQSAPLRVYVEELKPTPEGDLE
AQSAPLRVYVEELKPTPEGDLEI
ASDISLLDAQSAPLRVYVEELKPTPEGDLEI
ASDISLLDAQSAPLRVYVEELKPTPEGDLEIL
ASPEVIESPPEINT
DAIPENLPPLTAD
DALNENKVLV
DALNENKVLVLDTDY
DAQSAPLRVYVE
DAQSAPLRVYVEELKPTPEGD
DAQSAPLRVYVEELKPTPEGDL
DAQSAPLRVYVEELKPTPEGDLE
DAQSAPLRVYVEELKPTPEGDLEI
DAQSAPLRVYVEELKPTPEGDLEIL
DAQSAPLRVYVEQLKPTPEGDL
DDEALEKFDKAL
DEALEKFDKALKALPMHIRLSFNPTQL
DELQDKIHPF
DKTEIPTINT
DKVGINYW
DLKGYGGVSLPEW
DLSKEPSISRE
DLSKEPSISREDL
DSPEVIEGPPEINT
DSPEVIESPPEINT
DTHKSEIAHRFKDLGEEHFKG
EALEKFDKALKALPM
EALEKFDKALKALPMHI
EAMAPKHKEMPFPK
EAMAPKHKEMPFPKYPVE
EASPEVIESPPEIN

EDSPEVIEGPPEINT
EELKPTPEGD
EELKPTPEGDL
EELKPTPEGDLE
EELKPTPEGDLEI
EELKPTPEGDLEIL
EKFDKALKALPMHI
EKFDKALKALPMHIRL
ELEELNVPGEIVE
ELKPTPEGDL
ELKPTPEGDLE
ELKPTPEGDLEI
ELKPTPEGDLEIL
EMPFPKYPVE
EMPFPKYPVEPF
ENSAEPEQSLV
EPVLGPVRGPFPIIV
ESPPEINT
EVDDEALEK
EVIESPPEINT
FAQTQSLVYPFPGP
FVAPFPEV
FVAPFPEVFGKEK
FVAPFPEVFGKEKVNE
GLDIQK
GLDIQKVAGTWYSLA
GLDIQKVAGTWYSLAM
GLDIQKVAGTWYSLAMA
GVSKVKEAMAPKHKEMPFPKYPVE
HIRLSFNPTQ
HKEMPFPKYPVEPF
HLVDEPQNL
HNSLPQNIPPLTQT
IASGEPTSTPITE
IASGEPTSTPTIE
IASGEPTSTPTIEA
IASGEPTSTPTTE
IASGEPTSTPTTEA
IDALNENK
IDALNENKVLVLDTDY
IDALNENKVLVLDTDYKK
IESPPEINT
IIAEKTKIPAV
IIAEKTKIPAVFKIDA
IIAEKTKIPAVFKIDALNENKVLVLDTDYK
IIVTQTMKGLDIQ

IIVTQTMKGLDIQKV
IIVTQTMKGLDIQKVAGT
IIVTQTMKGLDIQKVAGTW
IIVTQTMKGLDIQKVAGTWYSLAM
IIVTQTMKGLDIQKVAGTWYSLAMAASD
IIVTQTMKGLDIQKVAGTWYSLAMAASDISLLD
ILNKPEDET
IQKVAGTWYSL
IQKVAGTWYSLA
ISLLDAQSAPLRVYVEELKPTPEGD
ISLLDAQSAPLRVYVEELKPTPEGDL
ISLLDAQSAPLRVYVEELKPTPEGDLE
IVQNNDSTEYG
IVTQTMKGLDIQ
KALKALPMHIRL
KFDKALKALPMHI
KGLDIQKVAGTW
KIDALNEN
KIDALNENKVL
KIDALNENKVLVL
KIDALNENKVLVLDTD
KIDALNENKVLVLDTDYK
KIDALNENKVLVLDTDYKKY
KIHPFAQTQSL
KNQDKTEIPTINT
KPTPEGDLEI
KPTPEGDLEIL
LDAQSAPLRVYVE
LDAQSAPLRVYVEE
LDAQSAPLRVYVEELKPTPEGD
LDAQSAPLRVYVEELKPTPEGDL
LDAQSAPLRVYVEELKPTPEGDLEI
LDAQSAPLRVYVEELKPTPEGDLEIL
LDAQSAPLRVYVEQLKPTPEGDLEIL
LDIQKVAGTW
LEDSPEVIESPPEINT
LEKFDKALKALPMHIRLSFNPTQL
LIVTQTMKGLD
LIVTQTMKGLDIQKVAGTWYSL
LIVTQTMKGLDIQKVAGTWYSLA
LIVTQTMKGLDIQKVAGTWYSLAM
LIVTQTMKGLDIQKVAGTWYSLAMA
LIVTQTMKGLDIQKVAGTWYSLAMAASD
LIVTQTMKGLDIQKVAGTWYSLAMAASDISL
LIVTQTMKGLDIQKVAGTWYSLAMAASDISLL
LKALPMHIRL

LKGYGGVSLPEW
LNENKVLV
LQDKIHPFAQTQSL
LSFNPTQLEEQC
LSFNPTQLEEQCHI
LTDVENLHLPLPLL
LVYPFPGPIPN
LVYPFPGPIPNSL
LVYPFPGPIPNSLPQNIPPLTQT
MAASDISLLDAQSAPL
MAASDISLLDAQSAPLRVYVEELKPTPEGDL
MAASDISLLDAQSAPLRVYVEELKPTPEGDLE
MAASDISLLDAQSAPLRVYVEELKPTPEGDLEIL
MAIPPK
MAIPPKKNQDKTEIPTINT
MELGHKIMRNLENTVKE
MELGHKIMRNLENTVKETIK
MELGHKIMRNLENTVKETIKYLKS
MELGHKIMRNLENTVKETIKYLKSL
MHQPHQPLPPTVM
NENKVLVL
NENKVLVLDTDYKK
NKVLVLDTDYKKYL
NQDKTEIPTINT
PPKKNQDKTEIPTINTI
PPLTQTPVVVPPFLQPEVMGVSKVKEAMAPKHKEMPF
PQNIPPLTQT
PQNIPPLTQTPVVVPPFIQPEV
PVLGPVRGPFPIIV
PVVVPPFIQPEVMG
PVVVPPFIQPEVMGV
PVVVPPFLQPEVMGV
PVVVPPFLQPEVMGVS
PVVVPPFLQPEVMGVSKVKE
PVVVPPFLQPEVMGVSKVKEAMAPK
PVVVPPFLQPEVMGVSKVKEAMAPKH
QDKIHPFAQTQSL
QEPVLGPVRGPFPILV
QINNKIWCKDDQNPHSSNICNISCDKFLDDDLTDDIMCVKKILDKVGINY
QLKPTPEGDL
QPQSQNPKLPLS
QSLVYPFPGPIHNSLPQNIPPLTQT
QSLVYPFPGPIPN
QSLVYPFPGPIPNSLPQNIPPLTQT
QTMKGLDIQKVAGTW
RELEELNVPGE

RELEELNVPGEIVE
RELKDLKGYGGVSLPEW
RTPEVDDEALEKFDKA
RVYVEELKPTPEGDLE
RVYVEELKPTPEGDLEILLQK
SAPLRVYVEELKPTPEGDL
SAPLRVYVEELKPTPEGDLEIL
SDIPNPIGSENSEK
SDISLLDAQSAPL
SDISLLDAQSAPLRV
SDISLLDAQSAPLRVYVEELKPTPEGD
SDISLLDAQSAPLRVYVEELKPTPEGDL
SDISLLDAQSAPLRVYVEELKPTPEGDLE
SDISLLDAQSAPLRVYVEELKPTPEGDLEIL
SFNPTQLEEQCHI
SLAMAASDISLL
SLAMAASDISLLD
SLAMAASDISLLDAQSAPL
SLPQNIPPLTQT
SLSQSKVLPVPEKAVPYPQRDMPIQA
SLVYPFPGPIHNSLPQNIPPLTQT
SLVYPFPGPIPNSLPQNIPPLTQT
SPEVIESPPEINT
SPPEINT
SQKFPKAEF
SSRQPQSQNPKLPL
SSRQPQSQNPKLPLSI
TDVENLHLPLPL
TDVENLHLPLPLL
TDVENLHLPPL
TEIPTIN
TEIPTINT
TLTDVENLHLPLPL
TMKGLDIQKVAGTW
TPEVDDEAL
TPEVDDEALE
TPEVDDEALEK
TPEVDDEALEKFD
TPEVDDEALEKFDKALKALPMHI
TPVVVPPFIQPEVM
TQTMKGLDIQKVAGT
TQTMKGLDIQKVAGTW
TVMENFVAFVDK
VATLEDSPEVIESPPEINT
VEELKPTPEGD
VEELKPTPEGDL

VEELKPTPEGDLE
VEELKPTPEGDLEI
VEELKPTPEGDLEIL
VEELKPTPEGDLEILL
VEELKPTPEGDLEILLQKWEND
VEELKPTPEGDLEILLQKWENDECAQKK
VKKILDKVGIN
VLDTDYKKYL
VLPVPQKAVPYPQRDMPIQA
VRTPEVDDE
VRTPEVDDEA
VRTPEVDDEAL
VRTPEVDDEALEKFD
VRTPEVDDEALEKFDKALKA
VRTPEVDDEALEKFDKALKALPM
VRTPEVDDEALEKFDKALKALPMH
VRTPEVDDEALEKFDKALKALPMHI
VRTPEVDDEALEKFDKALKALPMHIRL
VRTPEVDDEALEKFDKALKALPMHIRLSFNPTQ
VRTPEVDDEALEKFDKALKALPMHIRLSFNPTQL
VSKVKEAMAPK
VSKVKEAMAPKHK
VTQTMKGLDIQKVAGT
VTQTMKGLDIQKVAGTW
VYPFPGPIHNS
VYPFPGPIHNSL
VYPFPGPIHNSLPQ
VYPFPGPIHNSLPQNIPPLT
VYPFPGPIHNSLPQNIPPLTQTPVVVPPF
VYPFPGPIP
VYPFPGPIPN
VYPFPGPIPNS
VYPFPGPIPNSL
VYPFPGPIPNSLPQ
VYPFPGPIPNSLPQNIPPLTQ
VYPFPGPIPNSLPQNIPPLTQT
VYPFPGPIPNSLPQNIPPLTQTPV
VYVEELKPTPEGDLE
VYVEELKPTPEGDLEIL
VYVEELKPTPEGDLEILLQ
VYVEELKPTPEGDLEILLQKWEND
WMHQPHQPLPPTVM
WSVARLSQKFPKAEF
YPFPGPIHN
YPFPGPIPNSLPQNIPPLTQT
YSLAMAASDISLL

YSLAMAASDISLLDAQSAPL
YSLAMAASDISLLDAQSAPLR
YSLAMAASDISLLDAQSAPLRV
YSLAMAASDISLLDAQSAPLRVYVEELKPTPEGDL
YSLAMAASDISLLDAQSAPLRVYVEELKPTPEGDLEIL
YVEELKPTPEGDLE
YVEELKPTPEGDLEI
YVEELKPTPEGDLEILLQK
YVEQLKPTPEGDL
